# The evolution of euhermaphroditism in caridean shrimps: a molecular perspective of sexual systems and systematics

**DOI:** 10.1186/1471-2148-10-297

**Published:** 2010-09-29

**Authors:** G Curt Fiedler, Andrew L Rhyne, Ryoko Segawa, Tadashi Aotsuka, Nikolaos V Schizas

**Affiliations:** 1 University of Maryland University College, Asia Division USAG-J Unit 45013, Box 2786, Zama-shi, Kanagawa 228-0827, Japan; 2 Edgerton Research Laboratory, New England Aquarium, Central Wharf, Boston, MA 02101, USA; 3 Department of Biology and Marine Biology, Roger Williams University One Old Ferry Road, Bristol, RI 02809, USA; 4 Department of Biological Science, Graduate School of Science and Engineering, Tokyo Metropolitan University, 1-1 Minamiohsawa, Hachioji, Tokyo 192-0397, Japan; 5 Department of Marine Sciences, University of Puerto Rico, Mayagüez, Isla Magueyes Laboratories, CALL BOX 9000, Mayagüez, PR 00681-9000, USA

## Abstract

**Background:**

The hippolytid genus *Lysmata *is characterized by simultaneous hermaphroditism, a very rare sexual system among Decapoda. Specialized cleaning behavior is reported in a few pair-living species; these life history traits vary within the genus. Unfortunately, the systematics of *Lysmata *and the Hippolytidae itself are in contention, making it difficult to examine these taxa for trends in life history traits. A phylogeny of *Lysmata *and related taxa is needed, to clarify their evolutionary relationships and the origin of their unique sexual pattern. In this study, we present a molecular phylogenetic analysis among species of *Lysmata*, related genera, and several putative hippolytids. The analysis is based upon DNA sequences of two genes, 16S mtDNA and nuclear 28S rRNA. Phylogenetic trees were estimated using Bayesian Inference, Maximum Likelihood, and Maximum Parsimony.

**Results:**

Phylogenetic analysis of 29 species of *Lysmata*, eight genera of Hippolytidae and two genera of Barbouriidae based on a single (16S, 28S) and combined gene approach (16S+28S) indicates that three groups of *Lysmata *differentiate according to antennular morphology: (1) *Lysmata*, having a multi-segmented accessory branch, (2) *Hippolysmata *(prior to Chace 1972), with a one-segmented accessory branch, and (3) a third group of *Lysmata *outliers, with one-segmented unguiform accessory branch, and close affinity to the genera *Exhippolysmata *and *Lysmatella*. The monophyly of the clade bearing a multi-segmented accessory branch is robust. Within the short accessory branch clade, species with specialized cleaning behaviors form a monophyletic clade, however, the integrity of the clade was sensitive to alignment criteria. Other hippolytid and barbouriid genera used in the analysis are basal to these three groups, including one displaying simultaneous hermaphroditism (*Parhippolyte*). The two barbouriid species occur in a separate clade, but among hippolytid taxa.

**Conclusions:**

The data support the historical morphological division of *Lysmata *into clades based on accessory branch morphology. The position of the "cleaner" shrimps, indicates that specialized cleaning behavior is a derived trait. The topologies of the cladograms support the monophyly of the barbouriids, but do not support their elevation to familial status. Taxa ancestral to the genus *Lysmata *display simultaneous hermaphroditism, suggesting that this life history trait evolved outside the genus *Lysmata*.

## Background

The hippolytid shrimp genus, *Lysmata *(Risso, 1816), has attracted the attention of biologists for several decades. Members of this genus are small caridean shrimp and occur in tropical to warm temperate marine coastal waters worldwide. They are popular marine aquarium pets, with some species engaging in cleaning behavior of fishes. One species (*L. seticaudata*) was used as a model organism for ground breaking studies on sexual differentiation in Crustacea [1-3], with the mistaken impression that this species underwent male-to-female sex change, or protandric hermaphroditism (PH). For many years, PH was thought to be the only form of hermaphroditism in the decapod crustacea, albeit uncommon. This perception changed in the last decade with the discovery that the reproductive system of two *Lysmata *species was a form of simultaneous hermaphroditism, or euhermaphroditism [4,5]. This system has been confirmed in every *Lysmata *species examined (e.g. *L. amboinensis *[5], *L. wurdemanni *[4], *L. nilita *[6], *L. seticaudata *[6], *L. californica *[7], *L. bahia *[8], *L. intermedia *[8], *L. rafa *[9] and *L. holthuisi *[10]). Among confirmed euhermaphroditic *Lysmata *species, all individuals pass through a functional male phase early in life [4-7]. This is the impetus of the term "protandric simultaneous hermaphroditism" or "PSH" (e.g. [11]) to describe the system. The early male phase also contributed to the mistaken impression that *Lysmata *species were protandric hermaphrodites [1-3].

Bauer [12,13] postulated that the evolution of PSH in *Lysmata *was related to social systems and/or behavioral characteristics among members of the genus. He divided *Lysmata *into two informal, non-taxonomic ecological groupings: 1) low density, pair living, specialized "cleaner shrimps", with bright and contrasting coloration, including yellow and red colors and long white antenna, and famous for their ability to actively "clean" fish (e.g. *L. amboinensis*, *L. grabhami, L. debelius*, and *L. splendida*); 2) high density, group living, "peppermint shrimps", with color patterns consisting of semi-translucent bodies with longitudinal and lateral red bands (e.g. *L. wurdemanni*, *L. californica*, and *L. seticaudata*). Bauer [12,13] hypothesized that PSH must have evolved from a paired, cleaning ancestor species living at low densities with few opportunities to find mates and further suggested that group-living species diverged once or multiple times from these paired species. However, this explanation was made without phylogenetic inference for the genus *Lysmata *and its socioethological patterns. Furthermore, there are indications that PSH may have evolved outside the genus. Recent studies have shown that PSH occurs in the hippolytid genera *Exhippolysmata *Stebbing, 1916 [14,15], *Lysmatella *Borradaile, 1915 (Rhyne unpub.), and one barbouriid genus (previously a hippolytid) *Parhippolyte *Borradaile, 1899 (Onaga & Fiedler, unpub.). The presence of PSH within these few taxa suggests an opportunity to examine the evolution of this unique system via a molecular phylogenetic approach.

Unfortunately, the systematics of both genus *Lysmata *and the family Hippolytidae are still unsettled. Recent revisions in the caridean genus *Lysmata *have increased the number of species to nearly 40, an expansion of 33% over the last 10 years, and this has not abated [9,10,16,17]. Members of the genus *Lysmata *were originally split into two genera: *Hippolysmata *Stimpson, 1860 and *Lysmata*. These two genera were previously differentiated by the presence of a multi-segmented accessory antennal branch in *Lysmata *species, and the lack thereof in *Hippolysmata *species (see [18] for an example). Chace [19] placed *Hippolysmata *in synonymy of *Lysmata*, based upon a perceived wide intraspecific variation in the accessory branch morphology. However, Chace may have failed to properly delineate several species based on this character, which directly led to his misinterpretation (c.f. [20]). Furthermore, both generic names were in use two decades later by Holthuis [18].

The family Hippolytidae has also been under recent scrutiny. Christoffersen [21] concluded that the Hippolytidae are a polyphyletic group, based upon a detailed manual cladistic analysis of morphological characters. He went so far as to rearrange member genera between the superfamilies Alpheoidea Rafinesque, 1815 and Crangonoidea Haworth, 1825. He placed the genus *Lysmata *with the closely related *Lysmatella *and *Exhippolysmata *in its own family, the Lysmatidae Dana, 1852. Chace [22] did not agree with Christoffersen's rearrangement of taxa into new superfamilies. He performed a non-cladistic analysis of the 40 genera originally assigned to the Hippolytidae, examining 107 separate characters [22]. He concluded that the family was "reasonably homogenous", but agreed with Christoffersen's [21] suggestion to move the genera *Barbouria *Rathbun, 1912, *Janicea *Manning and Hart, 1984, and *Parhippolyte *from the Hippolytidae to a new family, Barbouriidae Christoffersen, 1987. Martin and Davis [23] recognized some of the inconsistencies detailed by Christoffersen [21], and use Barbouriidae in their classification of recent Crustacea. However, they kept the Barbouriidae within the superfamily Alpheoidea, because of similarities to hippolytids. Furthermore, Martin and Davis [23] kept the rest of the hippolytids intact, not recognizing any of Christoffersen's [21] other new families. More recently, in a phylogenetic analysis of the Infraorder Caridea based on 16S and 18S sequence data, the genus *Lysmata *formed a distinct clade, well separated from the other hippolytids [24], supporting Christoffersen's view of a paraphyletic Hippolytidae. Hence, the accepted phylogeny of the Hippolytidae and related taxa is as yet unresolved.

In this paper, we present a phylogeny of 29 *Lysmata *species and eight genera of related hippolytids and two barbouriids, based upon sequences from both mitochondrial and nuclear ribosomal gene sequences. Our use of two genes from independently evolving genomes, a thorough taxonomic coverage of the *Lysmata *and related genera, and a robust analysis in terms of alignment strategies improves upon a very recent preliminary phylogeny of the genus *Lysmata *[25]. We demonstrate that PSH evolved outside the genus *Lysmata*, as it is present in at least one ancestral taxon. Our phylogenetic analyses support the past division of *Lysmata *and *Hippolysmata *species based on the morphology of the antennular accessory flagellum, and the need for revision of both past and present Hippolytidae.

## Methods

### Taxon sampling

We obtained specimens from *Lysmata *and other hippolytid genera, from all over the world (Table 1). Hereafter, when discussing phylogenetic relationships we refer to the historical *Hippolysmata*/*Lysmata *taxonomic nomenclature (prior to [19]) based on the presence or absence of a multi-segmented accessory branch on the dorsolateral flagellum of the antennule. We differentiate *Lysmata *as ornamented with a short, one-segmented accessory branch, a long multi-segmented accessory branch, or unguiform accessory branch (newly described here). Most of the Indo-Pacific specimens were collected by the first author in Hawaii, Japan, and other Pacific locations; the majority of West Atlantic specimens were provided by AR. Other specimens were kindly provided by individuals from a variety of locations, including Indonesia, the Mediterranean, and Brazil. Many specimens were photographed prior to fixation, as color information is critical in the ultimate determination of species identity [16]. Species identities were determined using published descriptions (e.g. [16]), the most recent morphological keys (e.g. [16,22]), and descriptions of several new species [26] Specimens or portions of specimens were fixed in 80-100% ethanol by their respective sources. A small number of specimens were frozen for mitochondrial separation procedures (see below). Where possible, we included replicate specimens for each species, including confirmed specimens from different geographical regions. For example, *Lysmata wurdemanni *was sampled from two locations in Florida and one location in Texas.

**Table 1:** List of species, authorities, location of collections and GenBank Accession numbers used in the phylogenetic analyses for both 16S mtDNA and the 28S rDNA

16S Tree Identifier	Scientific Name	Authority	Location	16S	28S	Sexual System	Social System
**Family**	**Hippolytidae**	**Bate, 1888**					
	***Short branch***						

1	*Lysmata bahia*	Rhyne and Lin, 2006	Salvador, Brazil	**HQ315557**	-	PSH	Group
1	*Lysmata bahia*	Rhyne and Lin, 2006	Salvador, Brazil	**HQ315558**	-		
2	*Lysmata bahia*	Rhyne and Lin, 2006	Bocas Del Toro, Panama	EU861503	-		
3	*Lysmata ankeri*	Rhyne and Lin, 2006	Haiti	**HQ315597 (2)**	-	PSH	Group
3	*Lysmata ankeri*	Rhyne and Lin, 2006	SMEE (Haiti)	EU861501	-		
3	*Lysmata ankeri*	Rhyne and Lin, 2006	Haiti	**HQ315598**	-		
4	*Lysmata ankeri*	Rhyne and Lin, 2006	Bahia, Brazil	**HQ315599**	-		
4	*Lysmata ankeri*	Rhyne and Lin, 2006	Bahia, Brazil	**HQ315600 (2**)	-		
5	*Lysmata pederseni*	Rhyne and Lin, 2006	Florida Keys, FL, USA	EU135832	-	PSH	Pair?/Low
6	*Lysmata pederseni*	Rhyne and Lin, 2006	Carrie Bow, Belize	EU861504	-		
7	*Lysmata pederseni*	Rhyne and Lin, 2006	Florida Keys, FL, USA	**HQ315601**	-		
8	*Lysmata pederseni*	Rhyne and Lin, 2006	Florida Keys, FL, USA	**HQ315602**	-		
9	*Lysmata boggessi*	Rhyne and Lin, 2006	Hernando Beach, FL, USA	**HQ315603 (2)**	-	PSH	Group
9	*Lysmata boggessi*	Rhyne and Lin, 2006	St. Petersburg, FL, USA	EU861505	-		
9	*Lysmata boggessi*	Rhyne and Lin, 2006	Unknown	DQ079719	DQ079794		
10	*Lysmata rafa*	Rhyne and Anker, 2008	Florida Keys, FL, USA	**HQ315604**	-	PSH	Pair?/Low
11	*Lysmata rafa*	Rhyne and Anker, 2008	Aquarium store, FL, USA (Haiti)	EU861495	-		
12	*Lysmata wurdemanni*	(Gibbes, 1850)	St. Petersburg, FL, USA	EU861497	-	PSH	Group
12	*Lysmata wurdemanni*	(Gibbes, 1850)	Florida Keys, FL, USA	EU135811	-		
12	*Lysmata wurdemanni*	(Gibbes, 1850)	Port Aransas, TX, USA	EU861496	-		
12	*Lysmata wurdemanni*	(Gibbes, 1850)	Florida Keys, FL, USA	**HQ315605**	**HQ315624**		
13	*Lysmata wurdemanni*	(Gibbes, 1850)	Fort Pierce, FL, USA	EU861500	-		
13	*Lysmata wurdemanni*	(Gibbes, 1850)	Sebastian Inlet, FL, USA	EU135831	-		
14	*Lysmata wurdemanni*	(Gibbes, 1850)	Port Aransas, TX, USA	EU135796	-		
15	*Lysmata gracilirostris*	Wicksten 2000	Venao, Panama (Pacific)	EU861502	-	PSH?	?
16	*Lysmata nayaritensis*	Wicksten 2000	Chumical, Panama	EU861506	-	PSH	Group
17	*Lysmata amboinensis*	(De Man, 1888)	Bali	**HQ315589 (2)**	**HQ315622**	PSH	Pair/Low
17	*Lysmata amboinensis*	(De Man, 1888)	Philippines	EU861488	-		
18	*Lysmata amboinensis*	(De Man, 1888)	Java	EU861487	-		
19	*Lysmata grabhami*	(Gordon, 1935)	Florida, USA	**HQ315590**	**HQ315621**	PSH	Pair/Low
19	*Lysmata grabhami*	(Gordon, 1935)	Brazil	**HQ315591**	-		
19	*Lysmata grabhami*	(Gordon, 1935)	Florida, USA	**HQ315592**	-		
19	*Lysmata grabhami*	(Gordon, 1935)	Haiti	EU861489	-		
20	*Lysmata grabhami*	(Gordon, 1935)	Brazil	**HQ315593**	-		
21	*Lysmata grabhami*	(Gordon, 1935)	Madeira, Portugal	EU861490	-		
22	*Lysmata debelius*	Bruce, 1983	Indo-Pacific	**HQ315594 (2)**	-	PSH	Pair/Low
22	*Lysmata debelius*	Bruce, 1983	Sri Lanka	**HQ315595**	-		
22	*Lysmata debelius*	Bruce, 1983	Philippines	EU861492	-		
22	*Lysmata debelius*	Bruce, 1983	Indo-Pacific	EU861491	-		
22	*Lysmata debelius*	Bruce, 1983	Unknown	DQ079718	DQ079793		
23	*Lysmata debelius*	Bruce, 1983	Java	EU861493	-		
24	*Lysmata californica*	(Stimpson, 1866)	La Jolla, CA, USA	**HQ315596 (2)**	-	PSH	Group
24	*Lysmata californica*	(Stimpson, 1866)	La Jolla, CA, USA	EU861498	-		
25	*Lysmata olavoi*	Fransen, 1991	Azores, Portugal	EU861494	-	PSH?	?

	***Long branch***						
26	*Lysmata cf. acicula †*	(Rathbun, 1906)	Lahi lahi Point, Oahu, HI, USA	**HQ315575**	-	PSH	Group
27	*Lysmata *cf. *trisetacea*	(Heller, 1861)	Kapapa Island, Oahu, HI, USA	**HQ315576**	**HQ315609**	PSH	Group
27	*Lysmata *cf. *trisetacea*	(Heller, 1861)	Kapapa Island, Oahu, HI, USA	**HQ315586**	-	PSH	Group
27	*Lysmata *cf. *trisetacea*	(Heller, 1861)	Kapapa Island, Oahu, HI, USA	**HQ315587**	-	PSH	Group
27	*Lysmata *cf. *trisetacea*	(Heller, 1861)	Kapapa Island, Oahu, HI, USA	**HQ315588**	-	PSH	Group
28	*Lysmata galapagensis*	Schmitt 1924	Nicaragua	**HQ315577 (2)**	**HQ315611**	PSH	Group
28	*Lysmata galapagensis*	Schmitt 1924	Islas Secas, Panama	EU861480	-		
29	*Lysmata moorei*	(Rathbun, 1901)	Bahia, Brazil	**HQ315578 (2)**	-	PSH	Group
30	*Lysmata moorei*	(Rathbun, 1901)	Galeta, Panama	EU861481	-		
31	*Lysmata nilita*	Dohrn and Holthuis, 1950	Giglio, Italy	EU861482	-	PSH	?
32	*Lysmata intermedia*	(Kingsley, 1879)	Sebastian Inlet, FL, USA	**HQ315579**	-	PSH	Group
32	*Lysmata intermedia*	(Kingsley, 1879)	Sebastian Inlet, FL, USA	**HQ315580**	-		
33	*Lysmata intermedia*	(Kingsley, 1879)	Bocas Del Toro, Panama	EU861484	-		
34	*Lysmata *cf. intermedia*	(Kingsley, 1879)	Bahia, Brazil	**HQ315581**	-	PSH	Group
35	*Lysmata *cf. intermedia*	(Kingsley, 1879)	Puerto Rico	**HQ315582 (2)**	-		
36	*Lysmata holthuisi*	Anker et al., 2009	Chumical, Panama	EU861483	-	PSH	Group
37	*Lysmata seticaudata*	(Risso, 1816)	Cabo Raso, Cascais, Portugal	**HQ315583 (3)**	**HQ315612**	PSH	Group
37	*Lysmata seticaudata*	(Risso, 1816)	Cabo Raso, Cascais, Portugal	EU861486	-		
37	*Lysmata seticaudata*	(Risso, 1816)	Corsica, France	EU861485	-		
38	*Lysmata ternatensis*	De Man, 1902	Akajima, Keramas, Japan	**HQ315584**	**HQ315610**	PSH	Group
38	*Lysmata ternatensis*	De Man, 1902	Akajima, Keramas, Japan	**HQ315585**	-		

	***Short branch***						
39	*Exhippolysmata ophloporoides*	(Holthuis, 1948)	Espirito Santo, Brazil	**HQ315566 (2)**	**HQ315616**	PSH	Group
39	*Exhippolysmata ophloporoides*	(Holthuis, 1948)	Ubatuba Bay, Brazil	EU861510	-		
39	*Exhippolysmata ophloporoides*	(Holthuis, 1948)	Espirito Santo, Brazil	**HQ315567**	-		
39	*Exhippolysmata ophloporoides*	(Holthuis, 1948)	Espirito Santo, Brazil	**HQ315568**	-		
40	*Lysmatella prima*	Borradaile, 1915	Sulawesi, Indonesia	**HQ315569 (2)**	**HQ315614**	PSH	Group

	***Unguiform branch***						
41	*Lysmata Iipkei*	Okuno and Fiedler, 2010	Sesoko Island, Okinawa, Japan	**HQ315574 (2)**	**HQ315608**	PSH	Group
42	*Lysmata *cf. *anchisteus*	Chace, 1972	Kapapa Island, Oahu, HI, USA	**HQ315606 (2)**	**HQ315607**	PSH	Group
43	*Lysmata hochi*	Bazea and Anker, 2008	Long Key, FL, USA	EU861507	-	PSH	Group

	***No branch info***						

46	*Merguia rhizophorae*	(Rathbun, 1900)	Bocas Del Toro, Panama	EU861508	-	PH	Group
48	*Merguia oligodon*	(De Man, 1888)	Iriomote Island, Japan	**HQ315570**	**HQ315617**	PH	Group
49	*Alope orientalis*	(De Man, 1890)	Camp Cove, Sydney, Australia	**HQ315559**	**HQ315613**	?	?
50	*Hippolyte acuta*	(Stimpson, 1860)	Aburatsubo, Kanagawa, Japan	**HQ315561**	**HQ315618**		Group
51	*Hippolyte williamsi*	Schmitt 1924	Puerto Aldea, Chile	EU861512	-		Group
52	*Hippolyte inermis*	Leach, 1815	Venice Lagoon, Italy	EU861511	-	PH?	Group
53	*Tozeuma carolinense*	Kingsley, 1878	St. Petersburg, FL, USA	EU861513	-		Group
54	*Heptacarpus futilirostris*	(Bate, 1888)	Aburatsubo, Kanagawa, Japan	**HQ315562**	**HQ315619**		Group
55	*Heptacarpus geniculatus*	(Stimpson, 1860)	Hayama, Kanagawa, Japan	**HQ315563**	**HQ315620**		Group
56	*Heptacarpus palpator*	(Owen, 1839)	La Jolla, CA, USA	EU861509	-		Group
57	*Thor amboinensis*	(De Man, 1888)	Bise Point, Okinawa, Japan	**HQ315571**	-		Group
57	*Thor amboinensis*	(De Man, 1888)	Iriomote Island, Japan	**HQ315572**	-		Group
58	*Thor *cf. *manningi*	Chace, 1972	Puerto Rico	**HQ315573**	-	PPH	Group
**Family**	**Barbouriidae**	**Christoffersen, 1987**					
44	*Parhippolyte mistica*	(Clark, 1989)	Odo Point, Okinawa, Japan	**HQ315560**	**HQ315615**	PSH	Group
45	*Barbouria cubensis*	(von Martens, 1872)	San Salvador, Bahamas	**HQ315565**	**HQ315627**	PSH?	Group
**Family**	**Alpheidae**	**Rafinesque, 1815**					
59	*Synalpheus brevicarpus*	(Herrick, 1891)	Puerto Rico	**HQ315564**	**HQ315626**		Pair

Accessory antennal branch types are indicated for *Lysmata *and closely allied taxa. Numbers of specimens (#) sharing the same sequence are indicated after the Genbank Accession Number. Accession numbers in bold face type represent new sequences. (*) denotes new putative species. (†)We use *L*. cf. *acicula*, as *L. acicula *(Rathbun) is the prior synonym used for Hawaii *L. ternatensis*. Body coloration and our 16S data and indicate that Hawaii *L. ternatensis *differs from *L. ternatensis *from Okinawa, Japan. The assignment of species to families is based on [64] and the assignment of sexual systems is based on [65] or where sufficient information is present. Hermaphroditism types: PH = protandric, PPH = partial protandric, PSH = protandric simultaneous, blank cells = no information or gonochoristic.

We have also included representatives of the hippolytid genera *Alope*, *Exhippolysmata*, *Heptacarpus, Tozeuma*, and *Thor*, as well as two barbouriids (*Barbouria*, *Parhippolyte*) to explore their phylogenetic relationship with *Lysmata*. The snapping shrimp *Synalpheus brevicarpus *from the closely related Alpheidae [24] was selected as the designated outgroup (Table 1). The final data sets consist of a combination of our novel sequences with published sequences obtained from GenBank. The sources of the GenBank sequences are recent papers by Porter et al. [27], Baeza et al. [25], and Rhyne et al. [28]. Samples including taxonomic authority, location, and GenBank accession numbers are given in Table 1.

### Molecular Methods

DNA was isolated from individual specimens using one (or more) of three techniques, dependent upon sample condition, fixation method, and laboratory location. Total DNA extractions from EtOH-fixed specimens were performed in one of two ways: a) using the PureGene DNA isolation kit (Gentra) for fixed-tissue or b) via SDS & phenol/chloroform extraction [29,30]. When available, frozen samples were also subjected to preferential mtDNA extraction using the alkaline lysis procedure [31]. This procedure was used because of concerns that mtDNA sequences (i.e., 16S) were confounded by the presence of putative mitochondrial pseudogenes (numt) in several species [32,33].

The 16S region was amplified with the 1471-1472 primers [34]. The 28S region was amplified with "28S01" 5'-GACTACCCCCTGAATTTAAGCAT-3' and "28SP19F" 5'-GAGATTACCCGCCTAATTTAAGCAT-3' as forward primers paired with the reverse primer "28SR-02" 5'-CTCCTTGGTCCGTGTTTC-3'. PCR conditions were optimized for each gene-species combination via gradient PCR procedures. PCR products were assessed via electrophoresis of 2.5-5 μl of amplicon on a 0.7-1% agarose gel. Amplified bands were visualized under UV light and stored digitally. PCR products were cleaned of excess dNTPs, primers, and other impurities with one of two methods: a) enzymatic treatment with EXOSAP or b) silica gel extraction and wash [35]. All successful PCR products were processed for sequencing using the Big Dye 3.1 Terminator Cycle Sequencing Kit and the ethanol precipitate products were loaded into either an ABI 3130xl 16-capillary Genetic Analyzer or an ABI 377 DNA sequencer. DNA products were sequenced from both directions. Sequence traces were viewed and processed with Phrap/Phred/Consed software [36-38] or 4Peaks software [39] and the chromatographs were cross-checked during contig building. Identical sequences were collapsed in MacClade [40] and represented as one taxon in the analysis. We reconstructed phylogenies based on both the 16S and 28S data sets separately, and combined. Preliminary analysis of the 28S region in several species showed that there was either no variation or very little variation among closely related species, so representative species of each of the 3 main clades of the 16S tree were chosen for sequencing. DNA sequences were aligned in ClustalX [41] using the default parameters. The resulting alignments of 16S and 28S consisted of conserved and highly variable regions. Some of highly variable regions could not be aligned unequivocally and those regions were removed by Gblocks v 0.91b [42]. The Gblocks parameters for the 16S and 28S data sets were: minimum number of sequences for a conserved position (11/32), minimum number of sequences for a flanking position (11/32), maximum number of contiguous non-conserved positions (8/8), minimum length of a block (5/5), and allowed gap positions (with half/with half). We explored further the robustness of the phylogenetic signal of the datasets against a) the alignment deriving after highly variable regions were removed with even more stringent criteria by Gblocks and b) the alignment deriving from the default settings in ClustalX. All alignments are available as supplementary data (Additional File 1: Table S1).

We analyzed the phylogenetic relationships of the sequences by using MCMC-based Bayesian inference (BI) as implemented in MrBayes v. 3.2 [43] and maximum parsimony (MP) and maximum likelihood (ML) in PAUP* [44]. Data specific models of nucleotide evolution were evaluated with ModelTest [45] by the AIC criterion. In the BI of the combined data set (16S+28 S), each data partition was assigned a different model of substitution. The conditions for the Bayesian analysis were: three million generations, four simultaneous independent runs, and tree sampling every 1000th generation. For the Bayesian analysis of the concatenated data, different nucleotide substitution models were applied to each data partition. Graphs of ln(L) against number of generations were inspected to determine the burn-in factor. A consensus tree was calculated after discarding the first 10% trees as burn-in, which ensured that non-optimal trees were not included. Searches for the MP tree(s) run using the full heuristic option with 10 random replicates and for ML trees the fast stepwise addition option was used. The robustness of each clade was assessed with 100 replicates for ML and 1000 for MP of the non-parametric bootstrap procedure [46]. For each bootstrap replicate, in MP a heuristic search was performed with 10 random taxon addition sequences and TBR branch swapping and in ML, a heuristic search was performed with the stepwise-addition option and TBR branch swapping. The Bayesian trees are presented and important topological discrepancies among the three phylogenetic methods are discussed. Posterior probabilities (p*P*) and bootstrap support (bp) values are used to indicate clade support.

## Results

We obtained 16S sequences from more than 100 specimens belonging to 29 species of *Lysmata *(27 named, 2 new), in addition to 16 species belonging to 8 other hippolytid genera and two genera of Barbouriidae, from all over the world (Table 1). In addition, we obtained partial sequences of the 28S ribosomal gene from 11 species of *Lysmata *and 9 species (eight genera) of other hippolytids and barbouriids (Table 1). The TrN + I + Γ and the GTR + Γ models of substitution were selected as the appropriate models for the 16S and 28S data sets, respectively.

**Figure 1:**
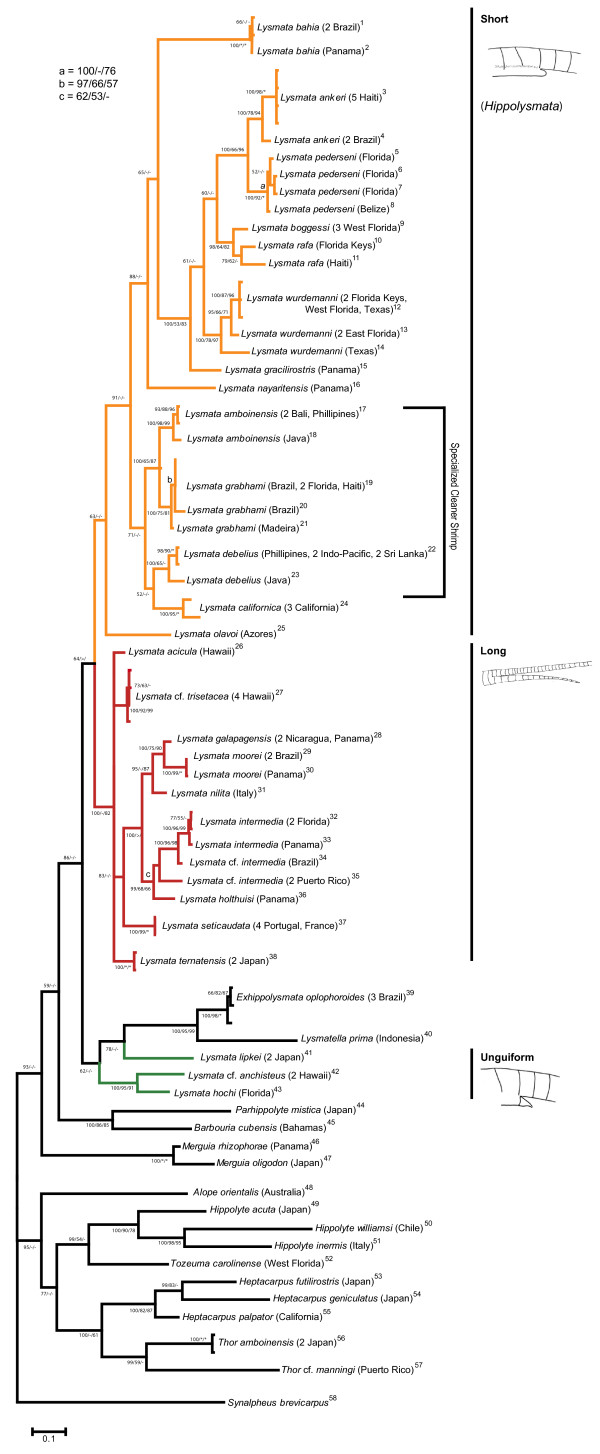
**Bayesian phylogeny of *Lysmata *and other related genera based on mitochondrial 16S sequences**. Highly variable alignment regions have been removed by GBlocks using less stringent criteria. Clade support values are shown along the corresponding branches (Bayesian Inference/Maximum Likelihood/Maximum Parsimony). Asterisks indicate 100% clade support for all three phylogenetic methods. Numbers before sample locations represent the number of specimens sequenced. Superscript numbers indicate which sequences/taxa are represented on the tree (see Tree Identifier in Table 1). Colored lines indicate *Lysmata *species. The orange clade represents those with a one-segmented (short) accessory branch, the red clade represents those with a multisegmented (long) accessory branch and the green clade represents those with a one-segmented unguiform (unguiform) branch. We define specialized cleaner shrimp as species with white legs and antennae and bright body coloration. The *Hippolysmata *correspond to the short accessory branch species. Species with an unguiform accessory branch were described after the synonymy of *Hippolysmata *with the genus *Lysmata*.

**Figure 2:**
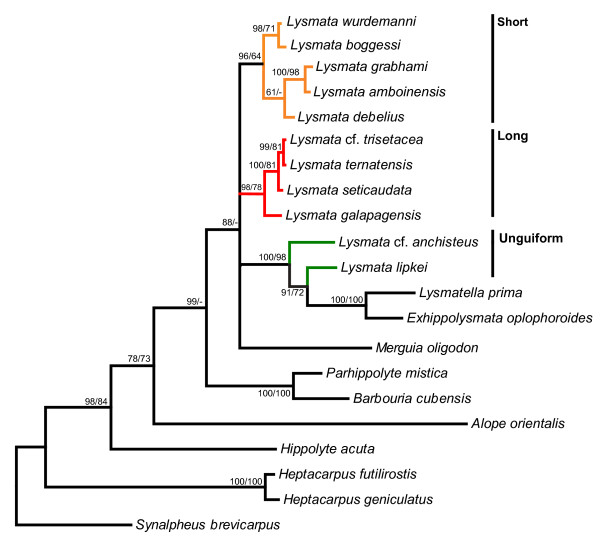
**Bayesian phylogeny of *Lysmata *and other related genera based on nuclear 28S sequences**. Highly variable alignment regions have been removed by GBlocks using less stringent criteria. Clade support values are shown along the corresponding branches (Bayesian Inference/Maximum Likelihood). Colored lines indicate *Lysmata *species. The orange clade represents those with a one-segmented (short) accessory branch, the red clade represents those with a multisegmented (long) accessory branch and the green clade represents those with a one-segmented unguiform (unguiform) branch.

**Figure 3:**
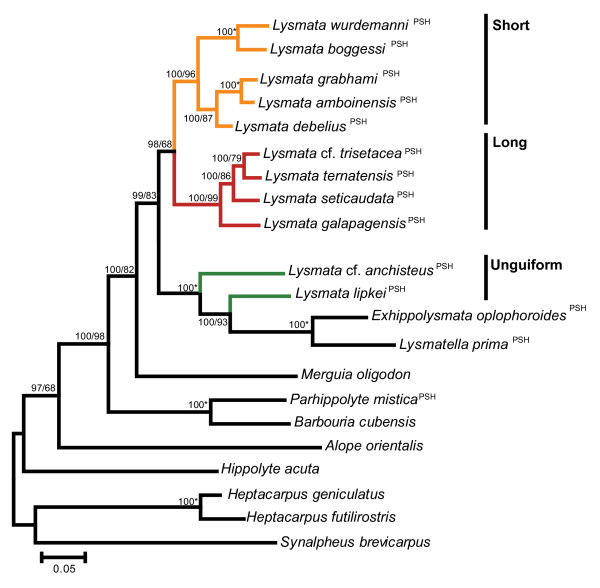
**Bayesian phylogeny of *Lysmata *and other related genera based on concatenated sequences of 16S/28S genes**. Highly variable alignment regions have been removed by GBlocks using less stringent criteria. Clade support values are shown along the corresponding branches (Bayesian Inference/Maximum Likelihood). Asterisks indicate 100% clade support for both phylogenetic methods. Colored lines indicate *Lysmata *species. The orange clade represents those with a one-segmented (short) accessory branch, the red clade represents those with a multisegmented (long) accessory branch and the green clade represents those with a one-segmented unguiform (unguiform) branch. PSH = protandric simultaneous hermaphroditism.

Phylogenetic analyses of 16S (Figure 1), 28S (Figure 2) and the concatenated data sets (16S+28 S; Figure 3) overall supported the historical division of the *Lysmata *based on antennular accessory branch morphology. The short accessory branch group includes *L. bahia*, *L. ankeri*, *L. pederseni, L. bogessi*, *L. rafa*, *L. wurdemanni, L. gracilorostris*, *L. nayaritensis*, *L. amboinensis*, *L. grabhami*, *L. debelius*, *L. californica*, and *L. olavoi *(Figure 1, p*P *= 63). The low clade values (p*P *< 50) supporting the basal position of *L. olavoi *with respect to species possessing a short accessory branch reflect the uncertainty of its placement in the phylogenetic tree (Figure 1). The *Lysmata *group ornamented with a long accessory branch consists of *L. galapagensis*, *L. moorei*, *L. nilita*, *L. intermedia*, *L. seticaudata*, *L ternatensis*, *L. trisetacea, L. acicula *and was recovered as a highly supported monophyletic group (Figure 1, p*P *= 100). The three species (*Lysmata hochi*, *L*. cf. *anchisteus*, and *L. lipkei*) that are ornamented with an unguiform branch are recovered outside *Lysmata *and clustered with *Exhippolysmata *and *Lysmatella *(Figure 1), but this topological arrangement is not consistent with all alignment strategies.

The analyses also support a behavioral split within the short accessory branch clade *- *the so-called "cleaner" *vs. "*peppermint" shrimps. The specialized cleaner shrimps are defined as species with white antennae and legs, and bright body coloration, where peppermint shrimps lack white antennae and legs, and bright body coloration [47]. The "peppermint" shrimps, which are represented in the 16S tree by *L. wurdemanni, L. boggessi, L. pederseni, L. ankeri, L. rafa*, *L. bahia, L. gracilirostris, L. nayaritensis *are differentiated from the "cleaners" *L. debelius, L. amboinensis *and *L. grabhami *as separate clades (p*P *= 88 and p*P *= 71, respectively). The placement of *L. californica*, which is considered a peppermint shrimp, is contingent to the alignment strategy of the 16S data. Different alignments placed this species ancestral to cleaners or ancestral to non-cleaners (Additional File 2: Figure S1) or nested within the cleaner clade, sister taxon to *L. debelius *(Figure 1). The phylogenetic divisions between short and long accessory branch clades and between behavioral groups within the short accessory branch clade are supported mainly by BI and not by the ML or the MP method.

The clade including *Exhippolysmata oplophoroides*, *Lysmatella prima*, and the three unguiform *Lysmata *is positioned outside of *Lysmata *(short and long accessory branch) but the placement is either weakly supported (p*P *= 62; Figure 1), or in different positions in alternative alignments (Additional File 2: Figure S1). This group of species, along with *Parhippolyte mistica*, *Barbouria cubensis*, and *Merguia *Kemp 1914, are basal to all *Lysmata*, except those ornamented with an unguiform accessory branch (Figure 1). Finally, the 16S topology indicates two pairs of sister taxa, the genera *Hippolyte *with *Tozeuma *Stimpson, 1860, and *Heptacarpus *Holmes, 1900 with *Thor *Kingsley, 1878.

The resulting phylogeny from the 28S data alone (Figure 2) displays a general topology similar to that observed from the 16S (Figure 1) and the concatenated 16S+28S data set (Figure 3). The same major clades are apparent among the *Lysmata*, *Lysmatella*, and *Exhippolysmata *taxa. The only exception is the relative ancestral/derived positions of these clades, which are not resolved. This loss of resolution may simply be due to the relatively more conserved 28S region. The concatenated data strongly support the behavioral division within the shrimps possessing a short accessory branch: cleaners (p*P *= 100, bp = 87) vs. peppermint shrimps (p*P *and bp = 100). These data also support the historical division (prior to [19]) between *Lysmata *(p*P *= 100, bp = 99) and *Hippolysmata *(p*P *= 100, bp = 96), though neither *L californica*, *L. olavoi *or *L. nayaritensis*) are included. *Lysmata *cf. *anchisteus *and *Lysmata lipkei *are clustered outside *Lysmata *and *Hippolysmata *forming a clade with *Exhippolysmata *and *Lysmatella*, an association observed in the 16S analysis. Similar to the 16S tree, *Merguia, Parhippolyte*, and *Barbouria *are basal to *Lysmata*.

## Discussion

### I. *Lysmata *taxonomy & phylogeny

#### A. Historical division between *Lysmata *&*Hippolysmata*

Our data generally support the historical division of *Lysmata *based on accessory flagellum morphology. It also partly supports Rhyne's [48] further division of *Lysmata *according to morphology and/or color pattern: (1) *Lysmata*, having a long accessory branch, (2) *Hippolysmata *(prior to [19]), having a short accessory branch and displaying typical peppermint color patterns, and (3) cleaner shrimps, within *Hippolysmata*, with a short accessory branch and displaying bright coloration with white antenna. However, support for these groupings is contingent upon analysis method and alignment strategy (Figure 1, Additional File 2: Figure S1). Specifically, the positions of three peppermint shrimp species are problematic. The inconsistent placement of *L. californica *and *L. nayaritensis *challenges the monophyly of the peppermint shrimps. Furthermore, the support for the monophyly of the species with short accessory branch is weakened by the variable topological position of *L. olavoi*. Regardless, the discovery of a putative third group with unguiform branch (see below), renders the genus *Lysmata *paraphyletic. The BI recovers the different groups more consistently than both ML and MP, especially in the combined dataset (Figure 3). However the absence of *L. californica*, *L. nayaritensis *and *L. olavoi *from the combined data set weakens the comparison between the 16S and the 16S/28S trees. For comparison, the Baeza et al. [25] analysis recovers *Exhippolysmata *and *L. hochi *within the clade with the short accessory branch. Another problematic taxon is *L. olavoi *which is placed (p*P *= 63) in a basal position in the group with short accessory branch (Figure 1). Unlike *L. californica*, there is no behavioral data for *L. olavoi*, which has only been collected with traps from >125 m depth in Azores [49]. *Lysmata olavoi *is placed ancestrally to all other *Lysmata *in [25]. Any interpretation of the current results and those of Baeza et al. [25] should be made cautiously, as the evolutionary nature of the ribosomal datasets (i.e. excessive indel events) may limit the phylogenetic information they can convey.

The "cleaners" (*L. amboinensis*, *L. debelius*, and *L. grabhami*) may form a monophyletic group [25], except for the inclusion of *L. californica *within the cleaner clade. The placement of *L. californica *is unresolved, because it is strongly influenced by the alignment strategy. *Lysmata californica *has "peppermint shrimp" characteristics, lacking white legs and antenna, having translucent body with red stripes and living in groups. The monophyly of the remaining peppermint shrimps (i.e., *L. wurdemanni*, *L. rafa*, *L. boggessi*, *L. pederseni*, *L. ankeri*, *L. bahia*, *L. gracilirostris*, *L. nayaritensis*) was strongly supported (p*P *= 88; Figure 1). In contrast, the support for a monophyletic clade with species bearing a long accessory branch was very robust (p*P *= 100) and insensitive to the alignment conditions and the dataset used.

The topologies based on ribosomal data are also sensitive to the inclusion of particular taxa. By including *Lysmatella prima *in the analysis, *L. hochi *is no longer the sister taxon of *Exhippolysmata*, as indicated in [25]. Rather, *Lysmatella *is the "new" sister taxon, whereas *L. hochi *is consistently grouped with *L. anchisteus *(Figure 1). There are arguments supporting that a denser phylogenetic sampling of taxa will generally improve the phylogenetic accuracy [50,51], but others highlight the importance of longer sequences rather than denser taxon sampling [52]. However, the addition of more sequence data without concomitantly increasing the sampled taxa can lead to strong systematic biases, producing highly supported, but incorrect or misleading topologies [53]. Without a doubt, more species and more genes will be added in the future and should better resolve the systematic inconsistencies of *Lysmata *and related genera. Besides the potential problem of limited taxa and gene sampling, the tree topology may be more influenced by the final alignment itself than by the phylogenetic reconstruction method [54,55]. There are several possible ways to resolve the problem of alignment uncertainty: 1) explore the effect of different alignment strategies, 2) removal of uncertain regions and/or 3) include protein coding genes where homologous alignment may be more objective by using the more conserved amino acid sequences. The alignment uncertainty of ribosomal data sets caused by the excessive indel events of the *Lysmata *phylogeny will be ameliorated when nuclear protein-coding genes are included in the analysis.

#### B. A possible third clade of *Lysmata*

Three *Lysmata *species (*L. anchisteus*, *L. hochi*, *L. lipkei*) are robustly placed outside the *Lysmata *+ *Hippolysmata *clade in the 16S phylogeny. Similarly, the 16S/28S concatenated phylogeny places *L*. cf. *anchisteus *and *L. lipkei *in the same clade with *Lysmatella *and *Exhippolysmata *outside of their traditional taxonomic boundaries (Figure 3); this grouping suggests that an additional clade might be formed by species with a highly reduced antennular accessory branch. *Lysmata anchisteus*, *L. hochi*, and *L. lipkei *possess a vestigial antennal flagella, at most one segment in length with an unguiform shape [[19,20,26,49], respectively]. The position of these three species suggests they are basal to the other clades of *Lysmata*. Data from morphologically similar species (e.g. *L. uncicornis *and *L. kuekenthali*) may help clarify the occurrence of this clade. These "outlier" *Lysmata *also present a challenge to any revision of the nomenclature of the genus. If one proposes to resurrect *Hippolysmata *and *Lysmata *to their previous status based upon phylogenetic data, the outliers could not be placed into either genus. A new genus may have to be erected, once their relationship with *Exhippolysmata *and *Lysmatella *is clarified. Alernatively, the presence of *Exhippolysmata *and *Lysmatella *in the putative third clade of *Lysmata*, may indicate that the generic definitions of these two genera based on raised basal crest (*Exhippolysmata*) and lack of epipods (*Lysmatella*) may be insufficient to raise these species to the genus level. Unlike the *Lysmata *species of the third clade that are ornamented with an unguiform antennal flagella, *Exhippolysmata *and *Lysmatella *have a short blunt flagella.

#### C. *Exhippolysmata *&*Lysmatella*

One of the surprising findings in [25] is the position of the genus *Exhippolysmata *within *Lysmata*, rendering the genus *Lysmata *paraphyletic. We have shown that the position of *Exhippolysmata *depends on the alignment strategy for 16S and the taxon sampling. Additionally, single gene approaches of closely related species should be interpreted cautiously as they often represent the phylogeny of the genes [56-58] or the organelles [59] and not the "true" organismal phylogeny. It is obvious that more genes and additional taxon sampling are needed to resolve the phylogenetic issues of *Lysmata *and other closely related genera. When the morphology of the two genera is taken in to consideration (raised basal crest in *Exhippolysmata*; lack of epipods on the first four pereiopods in *Lysmatella*) it seems highly improbable that species with vastly different morphological characters would be nested within a clade of *Lysmata*. Even though we present a phylogeny based on a denser taxon sampling and an additional gene from the independently evolved nuclear genome, our approach is still limited. We have proceeded by concatenating the two gene sequences prior to the phylogenetic analysis (i.e. total evidence approach), because it has been demonstrated empirically that concatenation of multiple genes often results in a single well-supported topology [60]. Theoretical work, however, has shown that especially when the coalescent process is highly variable from gene-to-gene [61], concatenation of data sets can produce inconsistent phylogenetic estimates [62].

### II. Hermaphroditism & Life History Patterns

Our data do not support any relationship between cleaning symbiosis or social system and the origin of PSH or the genus itself. Results from our phylogenetic analyses suggest that fish cleaning is a derived behavior within the short accessory branch clade. *Lysmata californica*, a peppermint shrimp that commonly associates with moray eels is placed within the clade that includes species living in pairs and bright coloration indicating strong specialized behavior (Figure 1). For comparison, *L. californica *is basal to the cleaner clade in the study of Baeza et al. [25]. The different placement of this taxon is an alignment artifact as both studies used different alignment criteria. There is also evidence of moray eel interactions with species bearing long accessory branch [63]. Since well-developed cleaning behavior evolved once within *Lysmata*, there seems to be no obvious connection of the so-called "paired cleaning species" with the origin of PSH; PSH is ubiquitous within *Lysmata *and likely evolved ancestrally to the genus. Furthermore, most of the *Lysmata *species examined would be classified as group living species, including taxa basal to the "paired cleaner" clade. The assumption that group size = mating system in nature should be substantiated with supporting observations of behavior under natural conditions.

PSH has been recently been reported in *Exhippolysmata *[15], *Lysmatella prima *(Rhyne, unpublished) and *Parhippolyte *(Onaga & Fiedler, unpub.), a genus placed ancestrally to *Lysmata*, regardless of the alignment strategy. Clearly, studies attempting to determine the origins of PSH must focus on related genera that are ancestral to *Lysmata*, a point that has been highlighted also in [25]. Christoffersen [21] subdivided the hippolytids into superfamily and families based on morphological comparisons. The placement of *Parhippolyte *and *Lysmata *in different families (Barbouriidae and Lysmatidae, respectively) would further support that PSH evolved well outside of *Lysmata *and could be far more common than previously considered. The cave dwelling, group-living shrimp genus *Parhippolyte *possesses PSH and all phylogenetic analyses support the ancestral position of this group relative to all *Lysmata *and *Exhippolysmata*. When Bauer [12,13,47] postulated the evolution of PSH within *Lysmata *and why there are two distinct ecological clades, he was unaware that PSH is secondary to the divisions within the genera. The evolution of PSH likely predates the diversification of *Lysmata *and may have little or no bearing on the evolution of different ecological groups within the genus. For *Lysmata*, the question is not how PSH is related to socio-ecological systems, but rather why pair living and specialized fish cleaning behavior evolved from a group living ancestor.

### III. Phylogenetic issues in the Hippolytidae and related taxa

Based on cladistic analysis, Christoffersen [21] split the hippolytid genera into several different families. Notably, *Lysmata*, *Calliasmata *Holthuis 1973, *Exhippolysmata*, and *Mimocaris *Vereshchaka 1997 were assigned to the family Lysmatidae, while *Barbouria*, *Parhippolyte*, among other genera were assigned to the Barbouriidae. These two families were included with the Processidae Ortmann, 1890 and Crangonidae, and the genera *Merguia *and *Glyphocrangon *Milne-Edwards 1881, in the superfamily Crangonoidea. The rest of the hippolytids are assigned by Christoffersen to various families within the superfamily Alpheoidea. Chace [22] rejected this wholesale rearrangement, though agreed with the erection of the Barbouriidae. This assertion was re-affirmed by Martin and Davis [23]. We consistently recover the branch of *Barbouria *+ *Parhippolyte *in all trees, therefore we cannot reject Christoffersen's suggestion for separating the Barbouriidae. However, it is not clear whether or not the level of differentiation from *Lysmata, Exhippolysmata, Lysmatella*, and *Merguia *is sufficient to propose a separate family. Although the relative placement of *Merguia *and the two barbouriids is susceptible to alignment strategies, they are clearly more closely related to *Lysmata *than the other hippolytid genera in our phylogenies. The ancestral relationship of *Merguia *to *Lysmata *and its basal position to the barbouriids could invalidate the Crangonoidea *sensu *Christoffersen. A denser sampling of taxa from Christoffersen's proposed groups may help to clarify the level of differentiation present among these taxa and others that have traditionally been a part of the Hippolytidae. Until more convincing conclusions can be drawn, the current delineations proposed by Martin and Davis [23] should be maintained.

## Conclusions

Our mitochondrial and nuclear ribosomal data generally support the historic morphological division of *Lysmata *based on accessory branch morphology. Shrimps within the short accessory branch clade differentiate according to behavior and color pattern. The monophyly of the *Lysmata *group which is bearing a multi-segmented accessory branch is strongly supported, underlying the taxonomic importance of this character. *Lysmata *with an unguiform accessory branch are part of a third clade which includes *Lysmatella *and *Exhippolysmata*. The third clade does not conform to the historic division between *Lysmata *and *Hippolysmata*. PSH is ubiquitous within *Lysmata *and occurs in Barbouriidae, suggesting that this rare reproductive system that evolved ancestrally to the genera *Lysmata, Exhippolysmata and Lysmatella*. The two representative species of barbouriids form a monophyletic group and are consistently placed within the Hyppolytidae, therefore not providing support for the family Barbouriidae. The ribosomal data provides a unique view of the phylogeny of *Lysmata *and life history traits, however, the position of some taxa is sensitive to alignment strategies.

## Authors' contributions

GCF and AR conceived the project and collected the specimens. RS, TA and NVS provided monetary support, facilities and contributed to the manuscript. GCF, RS and NVS generated the molecular data. GCF, AR and NVS carried out the analyses and wrote the manuscript. All authors read and approved the final manuscript.

## Supplementary Material

Additional file 1 **Table S1: Sequence alignment data for the phylogenies presented in the paper**. Includes 16S, 28S and 16S/28S concatenated data sets as a single MSWord file. Click here for file

Additional file 2 **Figure S1: Bayesian phylogenies of *Lysmata *and other related genera based on alternative alignment strategies of mitochondrial 16S sequences**. Tree A was constructed after the removal of highly variable alignment regions via GBlocks using the most stringent criteria. Tree B was constructed using the alignment resulting from the default settings in ClustalX. Clade support values are shown along the corresponding branches (Bayesian Inference/Maximum Likelihood/Maximum Parsimony). Numbers before sample locations represent the number of specimens sequenced. Superscript numbers indicate which sequences/taxa are represented on the tree (see Tree Identifier in Table 1). Click here for file
