# Supplementary material for: The evolution of euhermaphroditism in caridean shrimps: a molecular perspective of sexual systems and systematics
Source: BMC Evol Biol. 2010 Sep 29;10:297. doi: 10.1186/1471-2148-10-297 (PMC2958167; doi:10.1186/1471-2148-10-297)
Supplement: Additional file 1 — Table S1: Sequence alignment data for the phylogenies presented in the paper. Includes 16S, 28S and 16S/28S concatenated data sets as a single MSWord file. [file 1471-2148-10-297-S1.DOC]

**Note: Numbers before the species name denote the Tree Identifiers (See Table 1 and Figure 1).**

**16S Figure 1**

1_Lysmata_bahia GGGCCGCGGTATACTGACCGTGCAAAGGTAGCATAATAATTAGTTCTTTAATTGAGGGCTGGAATGAAAGGTTTACGAGAAGGTAGCTGTCTCTTTTATAAAGTTTGAATTTTACATTTAAGTGAGAAGGCTTAAATAAGATAAAGGGACGAGAAGACCCTATAAAACTTTACAGCTTTCCCTTTTGTAG--------------TTGTTTCGTTGGGGCAATGGGCATAAAATAAATTAACTGTGTCTGTAATATAAATATAATTAGATTATTTGATCCCTTAGTAGGGATTTAGAGATTAAGTTACTTTAGGGATAACAGCGTAATTTCTCTTGAGAGTCCAAATCGACAGAGTTAGTTGCGACCTCGATGTTGAATTAAGATGTTAGCTAGGTGCAGGAGCCTAGATAGTAGGTCTGTTCGACCTTTAAAATCTT

1_Lysmata_bahia GGGCCGCGGTATACTGACCGTGCAAAGGTAGCATAATAATTAGTTCTTTAATTGAGGGCTGGAATGAAAGGTTTACGAGAAGGTAGCTGTCTCTTTTATAAAGTTTGAATTTTACATTTAAGTGAGAAGGCTTAAATAAGATAAAGGGACGAGAAGACCCTATAAAACTTTACAGCTTTCCCTTTTGTAG--------------TTGTTTCGTTGGGGCAATGGGCATAAAATAAATTAACTGTGTCTGTAATATAAATATAATTAGATTATTTGATCCCTTAGTAGGGATTTAGAGATTAAGTTACTTTAGGGATAACAGCGTAATTTCTCTTGAGAGTCCAAATCGACAGAGTTAGTTGCGACCTCGATGTTGAATTAAGATGTTAGCTAGGTGCAGGAGCCTAGATAGTAGGTCTGTTCGACCTTTAAAATCTT

2_Lysmata_bahia GGGCCGCGGTATACTGACCGTGCAAAGGTAGCATAATAATTAGTTCTTTAATTGAGGGCTGGAATGAAAGGTTTACGAGAAGGTAGCTGTCTCTTTTATAAAGTTTGAATTTTACATTTAAGTGAGAAGGCTTAAATAAGATAAAGGGACGAGAAGACCCTATAAAACTTTACAGCTTTCCCTTTTGTAG--------------CTGTTTCGTTGGGGCAATGGGCATAAAATAAATTAACTGTGTCTGTAATATAAATATAATTAGATTATTTGATCCCTTAGTAGGGATTTAGAGATTAAGTTACTTTAGGGATAACAGCGTAATTTCTCTTGAGAGTCCAAATCGACAGAGTTAGTTGCGACCTCGATGTTGAATTAAGATGTTAGCTAGGTGCAGGAGCCTAGATAGTAGGTCTGTTCGACCTTTAAAATCTT

49_Alope_orientalis GGATCGCGGTATTCTGACCGTGCGAAGGTAGCATCATCAATAGTCTTTTAATTGGAGTCTGGAATGAACGATGGACAAAAAGTAATCTGTCTTGAGAGTAAAAATTGAAATTCACTTTTAAGTGAGAAGGCTTAAATAAGTTAGAGGGACGATAAGACCCTATAAAACTTTATGA---CCTATCTAATTTTAATATTAAGATAGTCGTTTTATTGGGGCGATAAAAATATAATCAATTAACTGTTTGTAATAAA-T---ATATTTAAA-ATTGTGATCCTGTATTATAGATTAGTAGATAAAGTTCCTTTAGGGATAACAGCGTTATTCTTTCTGAGAGTTCTTATCGACGAAGGTAGTTGCGACCTCGATGTTGAATTAAAATTCCTTTTAGGTNNNNNNNNNNNNNNNNNNNNNNNNNNNNNNNNNNNNNNNNNN

44_Parhippolyte_mistica GGACCGCGGTACAATAACCGTGCGAAGGTAGCATCATCATTTGTCTTTTAATTGAAGGCTGGAATGAATGGTGAACGAGAAGTAAGCTGTCTCTTTAATGTATACTGAATTTCACCTTTAAGTGAAAAGGCTTAAATGTCACGGGGGGACGATAAGACCCTATAAAGCTTTACGA-----TTACTACTGTCTAATT--TGAGGTTCGTTTCGTTGGGGCGACGAGAATATAA-ACAGTAACTGT-TCTAATAAAACGGTATCGGTTGAG--TG-GACCCTTTATTAAGGATCATTAGATTAAGTTCCTTTAGGGATAACAGCGTTATTTCCCTTGAGAGTTCATATCGACAGGGTAAGTTGCGACCTCGATGTTGAATTAAGGTTCCATTCAGGTNNNNNNNNNNNNNNNNNNNNNNNNNNNNNNNNNNNNNNNNNN

50_Hippolyte_acuta GGGCTGCGGTAACTTGACCGTGCTAAGGTAGCATNATCAGTAGTCTTTTAATTGATGACTGGAATGAATGGCTTACGAGAAATAAGCTGTCTTAAAAATAAAAATTGAATTTNACCTTCAAGTGAAAAGGCTTGAATACTACTAAGGGACGATAAGACCCTATAAAACTTAA------CTTTTCTGGTTAAAAATTAAGAATGGTTGTTTGGTTGGGGCGACCTAGATATAA-TNAGTAACTGTCTGA-TAAAAATAATATAATTTG-GTTCATGAACCTTTAATAAGGATTAGAAGAAAAAGTTNCTTTAGGGATAACAGCGTAATTTTTTCAGAGAGTTCTTATCGAAGAAAGTAGTTGCGACCTCGATGTTGAATTAAAGTTNCTCTTAAGCNNNNNNNNNNNNNNNNNNNNNNNNNNNNNNNNNNNNNNNNNN

54_Heptacarpus_futilirostris CGACCGCGGTAATCTGACCGTGCGAAGGTAGCATCATCAGTAGTCTCTTAATTGGAGGCTGGAATGAATGGTGCACGAGAAGAAAGCTGTTTCTTCTTCAAGAATTGAATTTCACTTTTAAGTGAAAAGGCTTAAATAAATTGAAGGGACGATAAGACCCTATAAAACTTTA------ATAAATTAGTATAA-CTTGGGTTAGTTTGTTGAGTTGGGGCGACTATTATAAAA-TAA-TAACTGTATTCATTAATATT--CTGTTTAGTTAATT-GATCCTTTTTTAAAGATTAAAAGATTAAGTTCCTTTAGGGATAACAGCGTGATTTTTTTAAAGAGTTCTTATCGATGAAATTAGTTGCGACCTCGATGTTGAATTAAAATTCCTATAAAGTNNNNNNNNNNNNNNNNNNNNNNNNNNNNNNNNNNNNNNNNNN

56_Heptacarpus_palpator GGGCCGCGGTATCTTAACCGTGCGAAGGTAGCATAATCAATAGTCTTTTAATTGGAGGCTGGAATGAATGGCGCACGAGAAGTTAGCTGTCTCTTTTTTAGAGATTGAATTTTACTTTTAAGTGAAAAGGCTTAAATAAACTAAAGGGACGATAAGACCCTATAAAACTTTACAATTTACAAATTGGTATAA-CTAGGTAAGATTTGTTGGGTTGGGGCGACTATTATATAATTAA-TAACTGTAATTATTAATAGAT-TTATTTAGTTTA-ATGATCCTTTT-TAAAGATTAAAAGATTAAGTTACTTTAGGGATAACAGCGTAATTTTTTCTTAGAGTTCCTATCGACGAAATTAGTTGCGACCTCGATGTTGAATTAAAATTTCTATAAAGTGTAGCAGCTTACTTAGTTGGTCTGTTCGACCATTAAAATTTT

55_Heptacarpus_geniculatus cgacCGCGGTATTTTGACCGTGCGAAGGTACAcMBATCAATAGTCTCTTAATTAGAGGCTGGAATGAATGGTGCACGAGAAAGAAGCTGTCTCTTCTTTAAGAATTGACAcMBACTTTTAAGTGAAAAGGCTTAAATAATCTAAGGGGACGATAAGACCCTATAAAACTTTACAATTTATGAATTCGTCTAA--TAAGTGAAATTTGTTAGGTTGGGGCGACTATTATATAG-TAAC-AACTGTAATTAGAAATAGTT--TATTTAGTTAA-TTGATCCTTTAATAAAGATTATAAGATTACAcMBCTTTAGGGATAACAGCGTAATTTTTTCTGAGAGTTCTTATCGATGAAATTAGTTGCGACCTCGATGTTGAATTAACAcMBCTTTTNNNNNNNNNNNNNNNNNNNNNNNNNNNNNNNNNNNNNNNNNNNNNN

59_Synalpheus_brevicarpus GGACTGCGGTAATTTGACCGTGCAAAGGTAGCATAATCAATAGTCTTTTAATTGGAGGCTTGGATGAAAGGTTGACGGATGAGGAGCTGTCTCTTTAGTTGTGTT-GAACTTAACGTTTGTGTGAAAAGGCATTAATGGTTTAGGGGGACGATAAGACCCTATAAAACTTAAC--------ATGTGGTGTTA-TTGGAGTTGTT-TGTTTTGCTGGGGCGGCACGAATATAATTTG-TAACTGT-TTTAAAAAAATA--TTGATTAATAGATTTGGTCCTTTATTAGGGAGTTAA-GAGTAAGTTACTTTAGGGATAACAGCGTAATTTTTCTTGAGAGTTCTTATCGAAGGAAGTAGTTGTGACCTCGATGTTGAATTAAATTTTCCCTTTGGTGNNNNNNNNNNNNNNNNNNNNNNNNNNNNNNNNNNNNNNNNN

45_Barbouria_cubensis GGGCCGCGGTAACCTGACCGTGCGAAGGTAGCATAATAATTTGTCTTTTAATTGAAGGCTCGTATGAATGGTGGACAAGAAGAAGGCTGTCTTATTAATGAAAGCTGAATTTTACTTTTAAGTGAGAAGGCTTAAATAAACCAAGGGGACGATGAGACCCTATAAAACTTTA------CTTAGGTCCTGTGA-----ATTAGAGTTGTTTTGTTGGGGCGACAGGAATAAAA-AAGGTAACTGTTCTTATGAAA-TGGTATGACCAG-GTAGATGATCTTTTTTTAAGGATTAAAAGAGTAAGTTACTTTAGGGATAACAGCGTTATTTCCCCTGAGAGGTCTTATCGACGGGGTAAGTTGCGACCTCGATGTTGAATTAAGGTTTCACCCAGACGNNNNNNNNNNNNNNNNNNNNNNNNNNNNNNNNNNNNNNNNN

39_Exhippolysmata_oplophoroides GGGCCGCGGTACTTTGACCGTGCAAAGGTAGCATAATCAGTAGTTCTTTAATTGGGAACTTGTATGAAGGGCGGACAAGAAGGAACCTGTCTCTAAAATAAAATTTGAATTTCACTTTTAAGTGAAAAGGCTTAAATAAAATAAGGGGACGATAAGACCCTATAAAACTTGA------ACTAACTAGTTCTA-TTAAAGGAGGGTTGTTTTACTGGGGCGGTAGTTATATAGTA-GA-AACTGTAAAGA-TAAA-TAATATAATTAGAGAATATGATCCCTTCCTGGGGATTAAAAGATAAAGTTACTTTAGGGATAACAGCGTTATTTCTCTTGAGAGTCCAAATCGACAGAGTAAGTTGCGACCTCGATGTTGAATCAAGATGTTATTCAGGTGCAGAAGCCTGAATTGTGGGTCTGTTCGACCTTTAAAATCTT

39_Exhippolysmata_oplophoroides GGGCCGCGGTACTTTGACCGTGCAAAGGTAGCATAATCAGTAGTTCTTTAATTGGGAACTTGTATGAAGGGCGGACAAGAAGGAACCTGTCTCTAAAATAAAATTTGAATTTCACTTTTAAGTGAAAAGGCTTAAATAGAATAAGGGGACGATAAGACCCTATAAAACTTGA------ACTAACTAGTTCTA-TTAAAGGAGGGTTGTTTTACTGGGGCGGTAGTTATATAGTA-GA-AACTGTAAAGA-TAAA-TAATATAATTAGAGAATATGATCCCTTCCTGGGGATTAAAAGATAAAGTTACTTTAGGGATAACAGCGTTATTTCTCTTGAGAGTCCAAATCGACAGAGTAAGTTGCGACCTCGATGTTGAATCAAGATGTTATTCAGGTGCAGAAGCCTGAATTGTGGGTCTGTTCGACCTTTAAAATCTT

39_Exhippolysmata_oplophoroides GGGCCGCGGTACTTTGACCGTGCAAAGGTAGCATAATCAGTAGTTCTTTAATTGGGAACTTGTATGAAGGGCGGACAAGAAGGAACCTGTCTCTAAAATAAAATTTGAATTTCACTTTTAAGTGAAAAGGCTTAAATAAAATAAGGGGACGATAAGACCCTATAAAACTTGA------ACTAACTAGTTCTA-CTAAAGGAGGGTTGTTTTACTGGGGCGGTAGTTATATAGTA-GA-AACTGTAAAGA-TAAA-TAATATAATTAGAAAATATGATCCCTTCTTGGGGATTAAAAGATAAAGTTACTTTAGGGATAACAGCGTTATTTCTCTTGAGAGTCCAAATCGACAGAGTAAGTTGCGACCTCGATGTTGAATCAAGATGTTATTCAGGTGCAGAAGCCTGAATTGTGGGTCTGTTCGACCTTTAAAATCTT

40_Lysmatella_prima GGGCCGCGGTATTATGACCGTGCAAAGGTAGCATAGTCACTAGTTCTTTAATTGGGTTCTGGAATGAATGGTGGACAAGAAGAAAACTGTCTAGAATATAAAATTAGAATTTTACTTTTAAGTGAGAAGGCTTAAATAAAATAAGGGGACGATAAGACCCTATAAAACTTGT------ATAAAGTACTCCTCAATT-ATGAAGGATGTTGTTCTGGGGCGGTGCTTATATAA-ATATAAACTATAAAA-TTACAATAGTATAATTAGATTATTAGACCCTTGTATAAGGATTTTGAGATAAAGTTACTTTAGGGATAACAGCGTTATTTCTCTTGAGAGTCCAAATCGACAGAGTAAGTTGCGACCTCGATGTTGAATCAAGATGTTATTCAGGTGGAGGAGCCTGAATTGTAGGTCTGTTCGACCTTTAAAATCTT

46_Merguia_rhizophorae AGGCCGCGGTATTTTGACCGTGCGAAGGTAGCATAATCAATAGTCTTTTAATTGAAGGCTGGAATGAATGGTGGACGAGATGGAGGCTGTCTCTATTATAAATTTTGAATTTTACTTTTAAGTGAAAAGGCTTAAATAATTTAGTGGGACGATAAGACCCTGTAAAGCTTTATAATTT------TTGAGTTA-CAAAATTAGTATTGGTTATTTGGGGTGACTAAGATATAATAAATTGAGAAACTGTATAAAAATAATATAATTAGTTTAAGTGATCCTTAATTTAGGATTATAAGATAAAGTTACTTCAGGGATAACAGCGTAATTTCTCTTGAGAGCACATATCGACAGAGTTAGTTGCGACCTCGATGTTGAATTAAGGTAATTGTTAGGCGCAGCAGTTTATAAAATAGGTCTGTTCGACCTTTAAAACCTT

48_Merguia_oligodon tggcCGCGGTATTTTGACCGTGCGAAGGTAGCACAATCAATAGTCTTTTAATTGAGGGCTGGAATGAATGGTGGACGAGATGGAGGCTGTCTCTAGCGTAAATCTTGAATTCAACTTTTAAGTGAAAAGGCTTAAATGATTTAGTGGGACGATAAGACCCTGTAAAGCTTCATAATTT------TTTACACA-CAAAATTAGTATCGATTATTTGGGGTGACTTAGATATAATCAATTGAGAAACTGTACAAAAATAATATAATTAGCTTAGGTGATCCTTAATTTAGGATTAAAAGATAAAGTCACTTCAGGGATAACAGCGTAATTTCTCTTGAGAGCACATATCGACAGAGTTAGTTGCGACCTCGATGTTGAATTAAGGTCATTTTTAGGNNNNNNNNNNNNNNNNNNNNNNNNNNNNNNNNNNNNNNNNNNN

57_Thor_amboinensis TGGCCGCGGTATTTTGACCGTGCGAAGGTAGCATAATCAGTTGTCTTTTAATTGAAGGCTGGAATGAAAGGTGCACGAGAAAAAATCTGTATTAATTATAAGTTTTGAATTTTACTTTTAAGTGAAAAGGCTTAAATATTTTAAAGGGACGATAAGACCCTATAAAGCTTTACAATTT-------AATTTTAAAATGATTTTCATAATTTTGTTGGGGTGATTATTATATATTAAA-TAACTGTAAATA-TAAA-TAGTATAATTAGTCTA-TTGATCCTTTTTTAAAGATTAAAAGATAAAGTTACTTTAGGGATAACAGCGTAATTTTTTCAGAGAGTTCTTATCGAAGAAAGTAGTTGCGACCTCGATGTTGAATTAAAATTTCTATTAAATGTAGCAGTTTAAGTAGTTGGTCTGTTCGACCATTAAAGTTTT

57_Thor_amboinensis TGGCCGCGGTATTTTGACCGTGCGAAGGTAGCATAATCAGTTGTCTTTTAATTGAAGGCTGGAATGAAAGGTGCACGAGAAAAAATCTGTATTAATTATAAGTTTTGAATTTT-CTTTTAAGTGAAAAGGCTTAAATATTTTAAAGGGACGATAAGACCCTATAAAGCTTTACAATTT-------AATTTTAAAATGATTTTCATAATTTTGTTGGGGTGATTATTATATATTAAA-TAACTGTAAATA-TAAA-TAGTATAATTAGTCTA-TTGATCCTTTTTTAAAGATTAAAAGATAAAGTTACTTTAGGGATAACAGCGTAATTTTTTCAGAGAGTTCTTATCGAAGAAAGTAGTTGCGACCTCGATGTTGAATTAAAATTTCTATTAAATGTAGCAGTTTAAGTAGTTGGTCTGTTCGACCATTAAAGTTTT

58_Thor_cf._manningi TGGCCGCGGTATTTTGACTGTGCAAAGGTAGCATAATCAGTTGTCTTTTAATTGGAGGCTGGAATGAAAGGTAAACGAGAAAAAAACTGTCTCAGCTTAAAGACTTGAATTTCCCCTTTAAGTGAAAAGGCTTAAATAATTTAAAGGGACGATAAGACCCTATAAAACTTTTATAATTATGATTTAGTTTAAAAGTTAA-AAGGTTATTTTATTGGGGTGATTATTATATAATATAATAACTGTAGTTATTAATATT--ATTATTAGAAAA-ATGATCCTTTAATAAGGATTAAAAGATAAAGTTACTTTAGGGATAACAGCGTTATTTTCTTGGAGAGTTCATATTGATAAGATAAGTTGCGACCTCGATGTTGAATTAAGGTGTCTTGTAAATGTAGCAGTTTATTAAGTTGGTCTGTTCGACCATTAAAACCTT

53_Tozeuma_carolinense GGGCCGCGGTAATTTGACCGTGCGAAGGTAGCATAATCAGTAGTCTCTTAATTGGGGGCTTGTATGAAAGGTGGACGAGAGGAAAGCTGTCTTTGTGATGGATCTTGAATTTTACTTTTAAGTGAAAAGTCTTAAATATACTTAAGGGACGATAAGACCCTGTAAAACTTAATAAGGC----TCTTGTTTAAAAAATATGAGTTTTATTTGGTTGGGGCGACTGGAATATAATATAGTAACTGTTCATAATAAA-TAATATAATTTGTAAACATGAGCCTTTATTAAGGATTAAAAGATAAAGTTACTTCAGGGATAACAGCGTGATTTTTTTTGAGAGTCCTTATCGACAAAAGTAGTTGCGACCTCGATGTTGAATTAAAATTTCTTTCAAATGCAGCAGTTTGATTAGTGGGTCTGTTCGACCTTTAAAATTTT

51_Hippolyte_williamsi TGGCTGCGGTAATTTGACCGTGCTAAGGTAGCATAATCAATAGTTTTCTAATTTAAAACTGGAATGAATGGTTGACGAAAAGCAAGCTTTTTTTAAAATATAAATTGAATTTTACTTCTGAGTGAAAAGGCTTAGATTCAGATAAGGGACGATAAGACCCTATAAAACTTAATAATAC-------TGTTTATAAATTATAAAGTTTATTTAGTTGGGGTGACTAAGATATAATAA--TAGCTGTCTCAAATAAA-----ATATTTTGTTATTAAATCCTTTTTTAAAGATTTAAA--ACTAAGTTACTTTAGGGATAACAGCGTAATTTTTTCTGAGAGTTCTTATCGAAGAAAATAGTTGCGACCTCGATGTTGAATTAAAATTTTTGATTAACGCAGATGTTAAAGAAATAGGTCTGTTCGACCTTTAAAATTTT

52_Hippolyte_inermis GGGCTGCGGTACTTTGACCGTGCTAAGGTAGCATAATCAATAGTCTCCTAACTAGGGACCGGAATGAACGGTTGACGAAAAGAAAGCTGTCTCTGAGGCATAGATTGAATTTTACTCTTAAGTGAAAAGGCTTAAATATTGTTAAGGGACGATAAGACCCTGTGAAACTTAATACTAT------------CA-TTTTATTATGGTTCTATTCATGGGGCGAC-----TAAAATAAAGTAGCTGTTTGAAGAAAAATAATGTAGTTTGTTATAAAAAGCTTTAAGTAAAGATTAGA--ACAAAGTTACTTCAGGGATAACAGCGTAATTTTTTCTGAGAGTTCATATCGAAGAAAGTAGTTGCGACCTCGATGTTGAATTAAAGTTTTTAGTAAGCGCAGCGGCTTAAAAAATAGGTCTGTTCGACCTTAAAAATTTT

41_Lysmata_lipkei GGGCCGCGGTATTTTGACCGTGCAAAGGTAGCATAATCAATAGTTTTTTAATTGAAAACTGGAATGAAAGGTGGACAAGAAGTAGACTGTCTCTTTTATAAGACTTGAATTTTACTTTTAAGTGAAAAGGCTTAAATGGTTTAGGGGGACGATAAGACCCTATAAAACTTAACAGATT-TTATTCAGGATAA--TTAATGTTTACTGTTTTACTGGGGCGGTACGGATAAAATAAAATAACTGTCTAT-TTATAATATTATAAATAGTAAATTTGATCCTTTACTAGGGATTAGGAGATAAAGTTACTTTAGGGATAACAGCGTAATCTCTCTTGAGAGTCCTAATCGACAGAGTAAGTTGCGACCTCGATGTTGAATTAAGGTGTTATCTAGGCGCAGAAGTCTAGACGGTAGGTCTGTTCGACCTTTAAAACCTT

26_Lysmata_acicula GGGCCGCGGTATCCTGACCGTGCGAAGGTAGCATAATCAATAGTGTTTTAATTGAAGACTGGAATGAAGGGTGGACAAGGGGTAAGCTGTCTCTAAAATAAATCTTGAAGTTTACCTTTAAGTGAAAAGGCTTAAATAAAGTAAAGGGACGATAAGACCCTATAAAACTTAACAAGTTATTAACTAGTGTAAACTTGATGGGTGTTGTTTTGTTGGGGCGACAGAGATAAAATGAATTAACTGTCTTTATTGTAATAGTATAATTAGAGAAATTGATCCTTTATTAAGGATTATAAGATTAAGTTACTTTAGGGATAACAGCGTAATTTCTCTTGAGAGTCCTAATCGACAGAGTTAGTTGCGACCTCGATGTTGAATTAAGATGTTATTCAGGCGCAGCAGTNNNNNNNNNNNNNNNNNNNNNNNNNNNNNNNNNN

27_Lysmata_cf._trisetacea GGGCCGCGGTATCCTGACCGTGCGAAGGTAGCATAATCAATAGTGTTTTAATTGAAGACTGGAATGAAGGGTGGACAAGGGGTTATCTGTCTCTTAGATAAATCTTGAAGTTTACTTTTAAGTGAAAAGGCTTAAATGAAGTAAGGGGACGATAAGACCCTATAAAACTTAACAAATTATTAACTAGTGTAAACTTGATGAGGGTTGTTTTGTTGGGGCGACAGGGATAAAATAAATTAACTGTCTTTATTAAAATAGTATAATTAGAGTATTTGATCCTTTAGTAAGGATTATAAGATTAAGTTACTTTAGGGATAACAGCGTAATTTCTCTTGAGAGTTNTAATCGACAGAGCTAGTTGCGACCTCGATGTTGAATTAAGATGTTATTCAGGCGCAGCAGTNNNNNNNNNNNNNNNNNNNNNNNNNNNNNNNNNN

28_Lysmata_galapagensis GGGCCGCGGTATACTGACCGTGCGAAGGTAGCATAATCAATAGTGTTTTAATTGAAGACTGGAATGAATGGTGGACAAGGGGTTTGCTGTCTCTAAGGTAAATCTTGAAGTTTACCTTTAAGTGAAAAGGCTTAAATGGCGTAAAGGGACGATAAGACCCTATAAAACTTGACGGATTATGAATTAGTGTAA-CTTGATAGTTG--GTTTTGTTGGGGCGACAGGGATATAATAAT-TAACTGTCTTT-TAAAAATAATGTAATTAGTTTAATTGATCCTTTATTAGGGATTACGAGATTAAGTTACTTTAGGGATAACAGCGTAATTTCTCTTGAGAGTTCTAATCGACAGAGTTAGTTGCGACCTCGATGTTGAATTAAGATGTTATTCAGGCGCAGAAGTCTGAAGGGTAGGTCTGTTCGACCTTTAAAATCTT

31_Lysmata_nilita GGGCCGCGGTATACTGACCGTGCGAAGGTAGCATAATCAATAGTGTTTTAATTGAAGACCGGAATGAATGGTGGACAAGGGGTTAGCTGTCTCTAAGGTAAGTCTNGAAGTTTACCTTTAAGTGAAAAGGCTTAAATGACGTAAAGGGACGATAAGACCCTATAAAACTTGACAAGTTATTGA-TAGTGTAAACTTGATGGTTGTTGTTTTGTTGGGGCGACAGGGATATAATCGG-TAACTGTCCTT-TGAAAATAGTATGGTTAGTTTAATTGATCCTCTATTAGGGATTATGAGATTAAGTTACTTTAGGGATAACAGCGTAATTTCTCTTGAGAGTTCTAATCGACAGAGTTAGTTGCGACCTCGATGTTGAATTAAGATGTTATTCAGGCGCAGGAGTCTGAAGGGTAGGTCTGTTCGACCTTTAAAATCTT

29_Lysmata_moorei GGGCCGCGGTATACTGACCGTGCGAAGGTAGCATAATCAATAGTGTTTTAATTGAAGACTGGAATGAAGGGTGGACAAGGGGCTTGCTGTCTCTAAGGTAAATCTTGAAGTTTACTTTTAAGTGAAAAGGCTTAAATGGCGTAGAGGGACGATAAGACCCTATAAAACTTAACGGATCATTAA---GTGTAA-CTTGATGGCCGTCGTTCTGTTGGGGCGACAGGGATATAATATGCTAACTGTCTTA-TAAAAATAGGGTAATTAGTTAAATTGATCCTTTATTAGGGATTATGAGATCAAGTTACTTTAGGGATAACAGCGTAATTTCTCTTGAGAGTTCTAATCGACAGAGTTAGTTGCGACCTCGATGTTGAATTAAGATGTTATTCAGGCGCAGAAGTCTGAATGGTAGGTCTGTTCGACCTTTAAAATCTT

30_Lysmata_moorei GGGCCGCGGTATACTGACCGTGCGAAGGTAGCATAATCAATAGTGTTTTAATTGAAGACTGGAATGAAGGGTGGACAAGGGGCTTGCTGTCTCTAAGGTAAATCTTGAAGTTTACTTTTAAGTGAAAAGGCTTAAATGGCGTAGAGGGACGATAAGACCCTATAAAACTTAACGGATCATTAA---GTGTAA-CTTGATGGCCGTCGTTCTGTTGGGGCGACAGGGATATAATATGCTAACTGTCTTA-TAAAAATAGGGTAATTAGTTAAATTGATCCTCTATTAGGGATTATGAGATCAAGTTACTTTAGGGATAACAGCGTAATTTCTCTTGAGAGTTCTAATCGACAGAGTTAGTTGCGACCTCGATGTTGAATTAAGATGTTATTCAGGCGCAGAAGTCTGAATGGTAGGTCTGTTCGACCTTTAAAATCTT

32_Lysmata_intermedia GGGCCGCGGTATACTGACCGTGCGAAGGTAGCATAATCAATAGTGTTTTAATTGGAGACTGGAATGAACGGTGGACAAAGGGTTTACTGTCTCTGAAATGAATCTTGAATTTTACTTTTAAGTGAAAAGGCTTAAATGACGTAAAGGGACGATAAGACCCTATAAAACTTGACAAGTTATTAGAGAGTGTAAACTTGATGGTCATTGTTTTGTTGGGGCGACAAGGATAAAATAAG-TAACTGTCTTT-TGCAAATAGCATGATTAGTTTATTTGATCCTCTACTAGGGATTAAGAGATTAAGTTACTTTAGGGATAACAGCGTAATTTCTCTTGAGAGTTCTTATCGACAGAGTTAGTTGCGACCTCGATGTTGAATTAAGATGTTATTTGGGCGCAGGAGTCCAAAAAGTAGGTCTGTTCGACCTTTAAAATCTT

33_Lysmata_intermedia GGGCCGCGGTATACTGACCGTGCGAAGGTAGCATAATCAATAGTGTTTTAATTGGAGACTGGAATGAACGGTGGACAAAGGGTTTACTGTCTCTGAAATGAATCTTGAATTTTACTTTTAAGTGAAAAGGCTTAAATGACGTAAAGGGACGATAAGACCCTATAAAACTTGACAAGTTATTAGAGAGTGTAAACTTGATGGTCATTGTTTTGTTGGGGCGACAAGGATAAAATAAG-TAACTGTCTTT-TGCAAATAGCATGATTAGTTTATTTGATCCTCTACTAGGGATTAAGAGATTAAGTTACTTTAGGGATAACAGCGTAATTTCTCTTGAGAGTTCTTATCGACAGAGTTAGTTGCGACCTCGATGTTGAATTAAGATGTTATTTGGGCGCAGGAGTCCGAAAAGTAGGTCTGTTCGACCTTTAAAATCTT

32_Lysmata_intermedia GGGCCGCGGTATACTGACCGTGCGAAGGTAGCATAATCAATAGTGTTTTAATTGGAGACTGGAATGAACGGTGGACAAAGGGTTTACTGTCTCTGAAATGAATCTTGAATTTTACTTTTAAGTGAAAAGGCTTAAATGACGTAAAGGGACGATAAGACCCTATAAAACTTGACAAGTTATTAGAGAGTGTAAACTTGATGGTCATTGTTTTGTTGGGGCGACAAGGATAAAATAAG-TAACTGTCTTT-TGCAAATAGCATGATTAGTTTATTTGATCCTTTACTAGGGATTAAGAGATTAAGTTACTTTAGGGATAACAGCGTAATTTCTCTTGAGAGTTCTTATCGACAGAGTTAGTTGCGACCTCGATGTTGAATTAAGATGTTATTTGGGCGCAGGAGTCCAAAAAGTAGGTCTGTTCGACCTTTAAAATCTT

34_Lysmata_cf_intermedia GGGCCGCGGTATACTGACCGTGCGAAGGTAGCATAATCAATAGTGTTTTAATTGGAGACTGGAATGAACGGTGGACAAAGGGTTTACTGTCTCTGAAATGAATCTTGAATTTTACTTTTAAGTGAAAAGGCTTAAATGACGTAAGGGGACGATAAGACCCTATAAAACTTGACAAGCTATTAGAGAGTGTAAACTTGATG--GTTTGTTTTGTTGGGGCGACAAGGATAAAATAAG-TAACTGTCTTTA-TAAA-TAGCATGATTAGTTTATTTGATCCTTTATTAGGGATTAAGAGATTAAGTTACTTTAGGGATAACAGCGTAATTTCTCTTGAGAGTTCTTATCGACAGAGTTAGTTGCGACCTCGATGTTGAATTAAGATGTTATTCGGGCGCAGGAGTCTGAAAAGTAGGTCTGTTCGACCTTTAAAATCTT

35_Lysmata_cf_intermedia GGGCCGCGGTATACTGACCGTGCGAAGGTAGCATAATCAATAGTGTTTTAATTGAAGACTGGAATGAACGGTGGACAAGGGGTTTACTGTCTCTAAAATGAATCTTGAAGTTTACTTTTAAGTGAGAAGGCTTAAATAATGTGGGGGGACGATAAGACCCTATAAAACTTGACAAGCTATTAAGTGGTGTAA-CTTGATGGTTGTTGTTTTGTTGGGGCGGCAAGGATAAAATAAG-TAACTGTCTTT-TAAAAATAGCATAATTAGTTTAGGTGATCCCTTATTAAGGATTAAGAGATTAAGTTACTTTAGGGATAACAGCGTAATTTCTCTTGAGAGTTCTAATCGACGGAGTTAGTTGCGACCTCGATGTTGAATTAAGATGTTATTTAGGTGCAGGAGTCTAAAGAGTAGGTCTGTTCGACCTTTAAAATCTT

36_Lysmata_holthuisi GGGCCGCGGTATATTGACCGTGCGAAGGTAGCATAATCAATAGTGTTTTAATTGGAGACTGGAATGAACGGTGGACAAGGGGTTTACTGTCTCTGTGATGGATCTTGAAGTTTACTTTTAAGTGAAAAGGCTTAAATAATGTAAAGGGACGATAAGACCCTATAAAACTTGACAAACTATTAATCAGTGTAAATTTGATG---GTTGTTTTGTTGGGGCGACAGGGATAAAATCAG-TAACTGTCTTT-TCAAAATAATATAATTAGTTTATTTGATCCTTTATTAAGGATTAGGAGATTAAGTTACTTTAGGGATAACAGCGTGATTTCTCTTGAGAGTTCTAATCGACAGAGTTAGTTGCGACCTCGATGTTGAATTAAGGTGTTATTCAGGCGCAGGAGTCTGAAGAGTAGGTCTGTTCGACCTTTAAAATCTT

37_Lysmata_seticaudata GGGCCGCGGTATACTGACCGTGCGAAGGTAGCATAATCAATAGTGTTTTAATTGAAGACTGGAATGAAGGGTGGACAAGGGGTTGGCTGTCTCTTAAATAAAATTTGAAGTTTACTTTTAAGTGAAAAGGCTTAAATAAAGTAGAGGGACGATAAGACCCTATAAAACTTTACAAGATATTATAAAGTGTAAACTTGATAAGAATTGTTTTGTTGGGGCGACAGGGATAAAA-AGATTAACTGTCTTTATAATAATAGTATAATTAGTTAAGTTGATCCTTTATTAAGGATTAAGAGATTAAGTTACTTTAGGGATAACAGCGTAATTTCTCTTGAGAGTTCTAATCGACAGAGTTAGTTGCGACCTCGATGTTGAATTAAGATGTTACTCAGGTGCAGCAGTCTGAGTGGTAGGTCTGTTCGACCTTTAAAATCTT

37_Lysmata_seticaudata GG-CCGCGGTATACTGACCGTGCGAAGGTAGCATAATCAATAGTGTTTTAATTGAAGACTGGAATGAAGGGTGGACAAGGGGTTGGCTGTCTCTTAAATAAAATTTGAAGTTTACTTTTAAGTGAAAAGGCTTAAATAAAGTAGAGGGACGATAAGACCCTATAAAACTTTACAAGATATTATAAAGTGTAAACTTGATAAGAATTGTTTTGTTGGGGCGACAGGGATAAAA-AGATTAACTGTCTTTATAATAATAGTATAATTAGTTAAGTTGATCCTTTATTAAGGATTAAGAGATTAAGTTACTTTAGGGATAACAGCGTAATTTCTCTTGAGAGTTCTAATCGACAGAGTTAGTTGCGACCTCGATGTTGAATTAAGATGTTACTCAGGTGCAGCAGTCTGAGTGGTAGGTCTGTTCGACCTTTAAAATCTT

38_Lysmata_ternatensis GGGCCGCGGTATCCTGACCGTGCGAAGGTAGCATAATCAATAGTGTTTTAATTGAAGACTGGAATGAAGGGTGGACAAGGGGTTAGCTGTCTCTAAATAAAATCTTGAAATTTACTTTTAAGTGAAAAGGCTTAAATAACGTGAGGGGACGATAAGACCCTATAAAACTTAACAAGTTATTAACTGGTGTAAACTTGATGGTAGTTGTTTTGTTGGGGCGACAGGGATAAAA-AGATTAACTGTCTTT-TTAAAATAGTATAATTAGTGAACTTGATCCTTTAATAAGGATTACAAGATTAAGTTACTTTAGGGATAACAGCGTAATTTCTCTTGAGAGTCCAAATCGACAGAGTTAGTTGCGACCTCGATGTTGAATTAAGATGTTATTCAGGCGCAGCCGTCTGAATAGTGGGTCTGTTCGACCTTTAAAATCTT

38_Lysmata_ternatensis GGGCCGCGGTATCCTGACCGTGCGAAGGTAGCATAATCAATAGTGTTTTAATTGAAGACTGGAATGAAGGGTGGACAAGGGGTTAGCTGTCTCTAGATAAAATCTTGAAATTTACTTTTAAGTGAAAAGGCTTAAATAACGTGAGGGGACGATAAGACCCTATAAAACTTAACAAGTTATTAACTGGTGTAAACTTGATGGTAGTTGTTTTGTTGGGGCGACAGGGATAAAA-AGATTAACTGTCTTT-TTAAAATAGTATAATTAGTGAACTTGATCCTTTAATAAGGATTACAAGATTAAGTTACTTTAGGGATAACAGCGTAATTTCTCTTGAGAGTCCAAATCGACAGAGTTAGTTGCGACCTCGATGTTGAATTAAGATGTTATTCAGGCGCAGCCGTCTGAATAGTGGGTCTGTTCGACCTTTAAAATCTT

27_Lysmata_cf._trisetacea GGGCCGCGGTATCCTGACCGTGCGAAGGTAGCATAATCAATAGTGTTTTAATTGAAGACTGGAATGAAGGGTGGACAAGGGGTTATCTGTCTCTTAGATAAATCTTGAAGTTTACTTTTAAGTGAAAAGGCTTAAATGAAGTAAGGGGACGATAAGACCCTATAAAACTTAACAAATTATTAACTAGTGTAAACTTGATGAGGGTTGTTTTGTTGGGGCGACAGGGATAAAATAAATTAACTGTCTTTATTAAAATAGTATAATTAGAGTATTTGATCCTTTAGTAAGGATTATAAGATTAAGTTACTTTAGGGATAACAGCGTAATTTCTCTTGAGAGTTCTAATCGACAGAGCTAGTTGCGACCTCGATGTTGAATTAAGATGTTATTCAGGCGCAGCAGTCTGAATGGTAGGTCTGTTCGACCTTTAAAATCTT

27_Lysmata_cf._trisetacea GGGCCGCGGTATCCTGACCGTGCGAAGGTAGCATAATCAATAGTGTTTTAATTGAAGACTGGAATGAAGGGTGGACAAGGGGTTATCTGTCTCTTAGATAAATCTTGAAGTTTACTTTTAAGTGAAAAGGCTTAAATGAAGTAAGGGGACGATAAGACCCTATAAAACTTAACAAATTATTAACTAGTGTAAACTTGATGAGGGTTGTTTTGTTGGGGCGACAGGGATAAAATAAATTAACTGTCTTTATTAAAATAGTATAATTAGAGTATTTGATCCTTTAGTAAGGATTATAAGATTAAGTTACTTTAGGGATAACAGCGTAATTTCTCTTGAGAGTTCTAATCGACAGAGCTAGTTGCGACCTCGATGTTGAATTAAGATGTTATTCAGGCGCAGCAGTCTGAATGGTAGGTCTGTTCGACCTTTAAAATCTT

27_Lysmata_cf._trisetacea GGGCCGCGGTATCCTGACCGTGCGAAGGTAGCATAATCAATAGTGTTTTAATTGAAGACTGGAATGAAGGGTGGACAAGGGGTTATCTGTCTCTTAGATAAATCTTGAAGTTTACTTTTAAGTGAAAAGGCTTAAATGAAGTAAGGGGACGATAAGACCCTATAAAACTTAACAAATTATTAACTAGTGTAAACTTGATGGGGGTTGTTTTGTTGGGGCGACAGGGATAAAATAAATTAACTGTCTTTATTAAAATAGTATAATTAGAGTATTTGATCCTTTAGTAAGGATTATAAGATTAAGTTACTTTAGGGATAACAGCGTAATTTCTCTTGAGAGTTCTAATCGACAGAGCTAGTTGCGACCTCGATGTTGAATTAAGATGTTATTCAGGCGCAGCAGTCTGAATGGTAGGTCTGTTCGACCTTTAAAATCTT

17_Lysmata_amboinensis GGGCCGCGGTATACTGACCGTGCGAAGGTAGCATAATCAATAGTTCTTTAATTGAGGACTGGAATGAAGGGTGGACGAGAAGTTAGCTGTCTCCAAGACAAGTCTTGAAGTTTACTTTTAAGTGAAAAGGCTTAAATGAGATAAAGGGACGATAAGACCCTATAAAACTTTACAGTTTTTAAATTCGTGTAAATCTGTTTTGGT-TGTTTTATTGGGGCGATAAGAATATAATCGATTAACTGTTTTTATGAAAATAGGATAATTAGTTAA-TTGATCCTTTAATAAGGATTAGAAGATTAAGTTACTTTAGGGATAACAGCGTAATTTCTCTTGAGAGTTCTAATCGACAGAGTTAGTTGCGACCTCGATGTTGAATTAAGGTGTTAGCTAGGCGCAGAAGCTTAGATAGTAGGTCTGTTCGACCTTTAAAACCTT

17_Lysmata_amboinensis GGGCCGCGGTATACTGACCGTGCGAAGGTAGCATAATCAATAGTTCTTTAATTGAGGACTGGAATGAAGGGTGGACGAGAAGTTAGCTGTCTCCAAGACAAGTCTTGAAGTTTACTTTTAAGTGAAAAGGCTTAAATGAGATAAAGGGACGATAAGACCCTATAAAACTTTACAGTTTTTAAATTCGTGTAAATCTGTTTTGGT-TGTTTTATTGGGGCGATAAGAATATAATCGATTAACTGTTTTTATGAAAATAGGATAATTAGTTAA-TTGATCCTTTAATAAGGATTAGAAGATTAAGTTACTTTAGGGATAACAGCGTAATTTCTCTTGAGAGTTCTAATCGACAGAGTTAGTTGCGACCTCGATGTTGAATTAAGGTGTTAGCTAGGCGCAGAAGCTTAGATAGTAGGTCTGTTCGACCTTTAAAACCTT

18_Lysmata_amboinensis GGGCCGCGGTATACTGACCGTGCGAAGGTAGCATAATCAATAGTTCTTTAATTGAGGACTGGAATGAAGGGTGGACGAGAAGTTAGCTGTCTCCAAGACAAGTCTTGAAGTTTACTTTTAAGTGAAAAGGCTTAAATAAAATAAAGGGACGATAAGACCCTATAAAACTTTACAGTTTTTAAATTNGTGTAAATCTGTTTTGGT-TGTTTTATTGGGGCGATAAGAATATAATCAATTAACTGTTTTT-TGAAAATAAGATAATTAGTTAA-TTGATCCTTTAATAAGGATTAANAGATTAAGTTACTTTAGGGATAACAGCGTAATTTCTCTTGAGAGTTCTAATCGACAGAGTTAGTTGCGACCTCGATGTTGAATTAAGGTGTTAGCTAGGCGCAGAGACTTAGATAGTAGGTCTGTTCGACCTTTAAAACCTT

19_Lysmata_grabhami GGGCCGCGGTATACTGACCGTGCGAAGGTAGCATAATCAATAGTTCTTTAATTGAGGACTGGAATGAAGGGTGGACGAGAAGTAAGCTGTCTCCAAGGCAAATCTTGAAGTTTACTTTTAAGTGAGAAGGCTTAAATAAGATAAAGGGACGATAAGACCCTATAAAACTTTACAGTATTTAAATT-GTGTAA-CTGTTTTAGCG-TGTTTTATTGGGGCGATAGGGATATAATCAATTAACTGTTTTTAATAAAA----ATAATTAGTTAA-CTGATCCTTTAATAAGGATTAGAAGATTAAGTTACTTTAGGGATAACAGCGTAATTTCTCTTGAGAGTTCTAATCGACAGAGTTAGTTGCGACCTCGATGTTGAATTAAGGTGTTAGCTAGGCGCAGAAGCTTAGATAGTAGGTCTGTTCGACCTTTAAAACCTT

19_Lysmata_grabhami GGGCCGCGGTATACTGACCGTGCGAAGGTAGCATAATCAATAGTTCTTTAATTGAGGACTGGAATGAAGGGTGGACGAGAAGTAAGCTGTCTCCAAGGCAAATCTTGAAGTTTACTTTTAAGTGAGAAGGCTTAAATAAGATAAAGGGACGATAAGACCCTATAAAACTTTACAGTATTTAAATT-GTGTAA-CTGTTTTAGCG-TGTTTTATTGGGGCGATAGGGATATAATCAATTAACTGTTTTTAATAAAA----ATAATTAGTTAA-CTGATCCTTTAATAAGGATTAGAAGATTAAGTTACTTTAGGGATAACAGCGTAATTTCTCTTGAGAGTTCTAATCGACAGAGTTAGTTGCGACCTCGATGTTGAATTAAGGTGTTAGCTAGGCGCAGAAGCTTAGATAGTAGGTCTGTTCGACCTTTAAAACCTT

19_Lysmata_grabhami GGGCCGCGGTATACTGACCGTGCGAAGGTAGCATAATCAATAGTTCTTTAATTGAGGACTGGAATGAAGGGTGGACGAGAAGTAAGCTGTCTCCAAGGCAAATCTTGAAGTTTACTTTTAAGTGAGAAGGCTTAAATAAGATAAAGGGACGATAAGACCCTATAAAACTTTACAGTATTTAAATT-GTGTAA-CTGTNTTAGCG-TGTTTTATTGGGGCGATANGNATATAATCAATTAACTGTTTTTAATAAAA----ATAATTAGTTAA-CTGATCCTTTAATAAGGATTAGAAGATTAAGTTACTTTAGGGATAACAGCGTAATTTCTCTTGAGAGTTCTAATCGACAGAGTTAGTTGCGACCTCGATGTTGAATTAAGGTGTTAGCTAGGCGCAGAAGCTTAGATAGTAGGTCTGTTCGACCTTTAAAACCTT

20_Lysmata_grabhami GGGCCGCGGTATACTGACCGTGCGAAGGTAGCATAATCAATAGTTCTTTAATTGAGGACTGGAATGAAGGGTGGACGAGAAGTAAGCTGTCTCCAAGGCAAATCTTGAAGTTTACTTTTAAGTGAGAAGGCTTAAATAAGATAAAGGGACGATAAGACCCTATAAAACTTTACAGTAT-------T-TGGAATTTAAATTAGTG-TGTTTTATTGGGGCGATAGGGATATAATCAATTAACTGTTTTTAATAAAA----ATAATTAGTTAA-CTGATCCTTTAATAAGGATTAGAAGATTAAGTTACTTTAGGGATAACAGCGTAATTTCTCTTGAGAGTTCTAATCGACAGAGTTAGTTGCGACCTCGATGTTGAATTAAGGTGTTAGCTAGGCGCAGAAGCTTAGATAGTAGGTCTGTTCGACCTTTAAAACCTT

21_Lysmata_grabhami GGGCCGCGGTATACTGACCGTGCGAAGGTAGCATAATCAATAGTTCTTTAATTGAGGACTGGAATGAAGGGTGGACGAGAAGTAAGCTGTCTCCAAGACAAATCTTGAAGTTTACTTTTAAGTGAAAAGGCTTAAATAAGATAAAGGGACGATAAGACCCTATAAAACTTTACAGTATTTAAATT-GTGTAA-CTGTTTTAGCG-TGTTTTATTGGGGCGATANGNATATAATCAATTAACTGTTTTTAATAAAA----ATAATTAGTTAATCTGATCCTTTAATAAGGATTANAAGATTAAGTTACTTTAGGGATAACAGCGTAATTTCTCTTGAGAGTTCTAATCGACAGAGTTAGTTGCGACCTCGATGTTGAATTAAGGTGTTAGCTAGGCGCAGAAGCTTAGATAGTAGGTCTGTTCGACCTTTAAAACCTT

22_Lysmata_debelius GGGCCGCGGTATATTGACCGTGCGAAGGTAGCATAATCAATAGTTCTTTAATTGAGGACTGGAATGAAGGGTGGACGAGAAGTAAGCTGTCTCTAAAATAAATCTTGAAGTTTACTTTTAAGTGAAAAGGCTTAAATAAGATAAAGGGACGATAAGACCCTATAAAACTTTACAGATTCTAAAATTGTTTGAAATT-ATGTGTA-TGTTTTGTTGGGGCGACAGAGATATAATTAATTAACTGTTTTTATTAAAATAATCTAATTAGTTAA-TTGATCCATTAATAAGGATTAAAAGATTAAGTTACTTTAGGGATAACAGCGTAATTTCTCTTGAGAGTTCTAATCGACAGAGTTAGTTGCGACCTCGATGTTGAATTAAGGTGTTAGCTAGGCGCAGAAGCTTAGATAGTAGGTCTGTTCGACCTTTAAAACCTT

23_Lysmata_debelius GGGCCGCGGTATATTGACCGTGCGAAGGTAGCATAATCAATAGT-CTTTAATTGAGGACTGGAATGAAGGGTGGACGAGAAGTAAGCTGTCTCTAAAATAAATCTTGAAGTTTACTTTTAAGTGAAAAGGCTTAAATAAGATAAAGGGACGATAAGACCCTATAAAACTTTACAGATTCTAAAATTGTTTGAAATT-ATGTGTA-TGTTTTGTTGGGGCGACAGAGATATAATTAATTAACTGTTTTTATTAAAATAATCTAATTAGTTAA-TTGATCCATTAATAAGGATTAAAAGATTAAGTTACTTTAGGGATAACAGCGTAATTTCTCTTGAGAGTTCTAATCGACAGAGTTAGTTGCGACCTCGATGTTGAATTAAGGTGTTAGCTAGGCGCAGAAGCTTAGATAGTAGGTCTGTTCGACCTTTAAAACCTT

22_Lysmata_debelius GGGCCGCGGTATATTGACCGTGCGAAGGTAGCATAATCAATAGTCCTTTAATTGGGGACTGGAATGAAGGGTGGACGAGAAGTAAGCTGTCTCTAAAATAAATCTTGAAGTTTACTTTTAAGTGAAAAGGCTTAAATAAGATAAAGGGACGATAAGACCCTATAAAACTTTACAGATT-------CATTTAAATTTGTTAGAAACTGTTTTGTTGGGGCGACAGAGATATAATTAATTAACTGTTTTTATTAAAATAATCTAATTAGTTAA-TTGATCCATTATTAAGGATTATAAGATTAAGTTACTTTAGGGATAACAGCGTAATTTCTCTTGAGAGTTCTAATCGACAGAGTTAGTTGCGACCTCGATGTTGAATTAAGGTGTTAGCTAGGCGCAGAAGCTTAGATAGTAGGTCTGTTCGACCTTTAAAACCTT

24_Lysmata_californica GGGCCGCGGTATACTGACCGTGCGAAGGTAGCATAATCATTAGTTCTTTAATTGAGGACTGGAATGAAAGGTGGACGAGAAGTTAACTGTCTCTAAAATAAATCTTGAAGTTTACTTTTAAGTGAAAAGGCTTAAATAAAATAAAGGGACGATAAGACCCTATAAAACTTTACAGGTC-------ATTTGAAACTCAATTAAGGCTGTTTTGTTGGGGCGATAGAGATATAATTAATTAACTGTTTTTATTAAAATAATCTAATTAGAAAA-ATGATCCTTTAATAAGGATTAAAAGATTAAGTTACTTTAGGGATAACAGCGTAATTTCTCTTGAGAGTTCCAATCGACAGAGTTAGTTGCGACCTCGATGTTGAATTAAGATGTTAGTTAGGCGCAGAAGCTTAAGTAGTAGGTCTGTTCGACCTTTAAAATCTT

24_Lysmata_californica GGGCCGCGGTATACTGACCGTGCGAAGGTATCATAATCATTAGTTCTTTAATTGAGGACTGGAATGAAAGGTGGACGAGAAGTTAACTGTCTCTAAAATAAATCTTGAAGTTTACTTTTAAGTGAAAAGGCTTAAATGAAATAAAGGGACGATAAGACCCTATAAAACTTTACAGGCC-------TTTGAAAATTCAATTAAGGCTGTTTTGTTGGGGCGATAGAGATATAATTGATTAACTGTTTTT-TTGAAATAATTTAATTAGGGAA-TTGATCCTTTAATAAGGATTAAAAGATTAAGTTACTTTAGGGATAACAGCGTAATTTCTCTTGAGAGTTCTGATCGACAGAGTTAGTTGCGACCTCGATGTTGAATTAAGATGTTAGTTAGGCGCAGAAGCTTAAGTAGTAGGTCTGTTCGACCTTTAAAATCTT

3_Lysmata_ankeri GGGCCGCGGTACACTGACCGTGCAAAGGTAGCATAGTAATTAGTTCTTTAATTGAGGACTGGGATGAACGGTGGACGAGAAGTTGGCTGTCTCTAGTATAAGTTTTGAATTTTACTTTTAAGTGAAAAGGCTTAAATAAAATAAAGGGACGATAAGACCCTATAAAACTTTACTTTCTGTAAA--AGTGTAAATTGCTTTTGGG-TGTTTTATTGGGGCGATAGAAATATAA-AATTTAACTGTTTTCATAATAATAATATAATTAGACTAAATGATCCTTTATTATGGAGTACGAGATTAAGTTACTTTAGGGATAACAGCGTAATTTCTCTTGAGAGTTCTAATCGACAGAGTTAGTTGCGACCTCGATGTTGAATTAAGATATCACTTAGGTGGAGGAGCTTAAGTAGTAGGTCTGTTCGACCTTTAAAATCTT

3_Lysmata_ankeri GGGCCGCGGTACACTGACCGTGCAAAGGTAGCATAGTAATTAGTTCTTTAATTGAGGACTGGGATGAACGGTGGACGAGAAGTTGGCTGTCTCTAGTATAAGTTTTGAATTTTACTTTTAAGTGAAAAGGCTTAAATAAAATAAAGGGACGATAAGACCCTATAAAACTTTACTTTCTGTAAA--AGTGTAAATTGCTTTTGGG-TGTTTTATTGGGGCGATAGAAATATAA-AATTTAACTGTTTTCATAATAATAATATAATTAGACTAAATGATCCTTTATTATGGAGTACGAGATTAAGTTACTTTAGGGATAACAGCGTAATTTCTCTTGAGAGTTCTAATCGACAGAGTTAGTTGCGACCTCGATGTTGAATTAAGATATCACTTAGGTGGAGGAGCTTAAGTAGTAGGTCTGTTCGACCTTTAAAATCTT

3_Lysmata_ankeri GGGCCGCGGTACACTGACCGTGCAAAGGTAGCATAGTAATTAGTTCTTTAATTGAGGACTGGGATGAACGGTGGACGAGAAGTTGGCTGTCTCTAGTATAAGTTTTGAATTTTACTTTTAAGTGAAAAGGCTTAAATAAAATAAAGGGACGATAAGACCCTATAAAACTTTACTTTCTGTAAA--AGTGTAAATTGCTTTTGGG-TGTTTTATTGGGGCGATAGAAATATAA-AATTTAACTGTTTTCATAATAATAATATAATTAGACTAAATGATCCTTTATTATGGAGTACGAGATTAAGTTACTTTAGGGATAACAGCGTAATTTCTCTTGAGAGTTCTAATCGACAGAGTTAGTTGCGACCTCGATGTTGAATTAAGATATCACTTAGGTGGAGGAGCTTAAGTAGTAGGTCTGTTCGACCTTTAAAATCTT

3_Lysmata_ankeri GGGCCGCGGTACACTGACCGTGCAAAGGTAGCATAGTAATTAGTTCTTTAATTGAGGACTGGGATGAACGGTGGACGAGAAGTTGGCTGTCTCTAGTATAAGTTTTGAATTTTACTTTTAAGTGAAAAGGCTTAAATAAAATAAAGGGACGATAAGACCCTATAAAACTTTACTTTCTGTAAA--AGTGTAAATTGCTTTTGGG-TGTTTTATTGGGGCGATAGAAATATAA-AATTTAACTGTTTTCATAATAATAATATAATTAGACTAAATGATCCTTTATTATGGAGTACGAGATTAAGTTACTTTAGGGATAACAGCGTAATTTCTCTTGAGAGTTCTAATCGACAGAGTTAGTTGCGACCTCGATGTTGAATTAAGATATCACTTAGGTGGAGGAGCTTAAGTAGTAGGTCTGTTCGACCTTTAAAATCTT

4_Lysmata_ankeri GGGCCGCGGTACCCTGACCGTGCAAAGGTAGCATAGTAATTAGTTCTTTAATTGAGGACTGGGATGAACGGTGGACGAGAAGTTGGCTGTCTCTAATATAAATTTTGAATTTTACTTTTAAGTGAAAAGGCTTAAATAAAATAAAGGGACGATAAGACCCTGTAAAACTTTACTTTCTGTAAA--AGTGTAAGCTT--TTAGGG-TGTTTTATTGGGGCGGTAGAAATATAA-AATTTAACTGTTTTCATAATAATAATATAATTAGACTAAATGATCCTTTACTATGGACTACGAGATTAAGTTACTTTAGGGATAACAGCGTAATTTCTCTTGAGAGTCCTAATCGACAGAGTTAGTTGCGACCTCGATGTTGAATTAAGATATCACCTAGGCGGAGGAGCTTAGGTAGTAGGTCTGTTCGACCTTTAAAATCTT

5_Lysmata_pederseni GGGCCGCGGTATACTGACCGTGCAAAGGTAGCATAATAATTAGTTCTTTAATTGAGGACTGGGATGAACGGTGGACGAGAAGTTAGCTGTCTCTAATATAAACTTTGAACTTTACCTATAAGTGAAAAGGCTTATATATAATAAAGGGACGATAAGACCCTATAAAACTTTACTTTCTAGTAAA-AGTGTAAGCTT--TTAGGG-TGTTTTATTGGGGCGATAGAAATATAA-ATATTAACTGTTTTT-TAATAATAGTATAATTAGAATAAGTGATCCTTTATTAAGGACTACAAGATTAAGTTACTTTAGGGATAACAGCGTAATTTCTCTTGAGAGACCTAATCGACAGAGTTAGTTGCGACCTCGATGTTGAATTAAGATGTTACTTAGGCGGAGGAGCTTAAGTAGTAGGTCTGTTCGACCTTTAAAATCTT

8_Lysmata_pederseni GGGCCGCGGTATACTGACCGTGCAAAGGTAGCATAATAATTAGTTCTTTAATTGAGGACTGGGATGAACGGTGGACGAGAAGTTAGCTGTCTCTAATATAAACTTTGAACTTTACCTATAAGTGAAAAGGCTTATATATAATAAAGGGACGATAAGACCCTATAAAACTTTACTTTCTAGTAAA-AGTGTAAGCTT--TTAGGG-TGTTTTATTGGGGCGATAGAAATATAA-ATATTAACTGTTTTT-TAATAATAGTATAATTAGAATAAGTGATCCTTTACTAAGGACTACAAGATTAAGTTACTTTAGGGATAACAGCGTAATTTCTCTTGAGAGACCTAATCGACAGAGTTAGTTGCGACCTCGATGTTGAATTAAGATGTCACTTAGGCGGAGGAGCTTAAGTAGTAGGTCTGTTCGACCTTTAAAATCTT

6_Lysmata_pederseni GGGCCGCGGTATACTGACCGTGCAAAGGTAGCATAATCATTAGTTCTTTAATTGAGGACTGGGATGAACGGTGGACGAGAAGTTAGCTGTCTCTAATATAAACTTTGAACTTTACCTTTAAGTGAAAAGGCTTATATATAATAAAGGGACGATAAGACCCTATAAAACTTTACTTTCTAGTAAA-AGTGTAAGCTT--TTAGGG-TGTTTTATTGGGGCGATAGAAATATAA-ATATTAACTGTTTTT-TAATAATAGTATAATTAGAATAAGTGATCCTCTACTAAGGACTACAAGATTAAGTTACTTTAGGGATAACAGCGTAATTTCTCTTGAGAGACCTAATCGACAGAGTTAGTTGCGACCTCGATGTTGAATTAAGATGTTACTTAGGCGGAGGAGCTTAAGTAGTAGGTCTGTTCGACCTTTAAAATCTT

7_Lysmata_pederseni GGGCCGCGGTATACTGACCGTGCAAAGGTAGCATAATCATTAGTTCTTTAATTGAGGACTGGGATGAACGGTGGACGAGAAGTTAGCTGTCTCTAATATAAACTTTGAACTTTACCTTTAAGTGAAAAGGCTTATATATAATAAAGGGACGATAAGACCCTATAAAACTTTACTTTCTAGTAAA-AGTGTAAGCTT--TTAGGG-TGTTTTATTGGGGCGATAGAAATATAA-ATATTAACTGTTTTT-TAATAATAGTATAATTAGAATAAGTGATCCTCTATTAAGGACTACAAGATTAAGTTACTTTAGGGATAACAGCGTAATTTCTCTTGAGAGACCTAATCGACAGAGTTAGTTGCGACCTCGATGTTGAATTAAGATGTTACTTAGGCGGAGGAGCTTAAGTAGTAGGTCTGTTCGACCTTTAAAATCTT

9_Lysmata_boggessi GGGCCGCGGTACTCTGACCGTGCAAAGGTAGCATAGTAATTAGTTCTTTAATTGAGGACTGGAATGAACGGTGGACGAGAAGTTAGCTGTCTCTAATGTAAATCTTGAAGTTTACTTTTAAGTGAAAAGGCTTAAATAATATAAAGGGACGATAAGACCCTATAAAACTTTACTTCTTATTAA--A---TAAATTTGCTTTTGGGTGTTTTGTTGGGGCGATAGAAATATAA--ATTTAACTGT-TTTATCAAAATAATATAATTAGTTTAAATGATCCTTTAGTATGGATTATAAGATTAAGTTACTTTAGGGATAACAGCGTAATTTCTCTTGAGAGACCTAATCGACAGAGTTAGTTGCGACCTCGATGTTGAATTAAGATATCATTTAGGTGTAGAAGTCTAAATAGTAGGTCTGTTCGACCTTTAAAATCTT

10_Lysmata_rafa GGGCCGCGGTACACTGACCGTGCGAAGGTAGCATAGTAATTAGTTCTTTAATTGAGGACTGGAATGAACGGTGGACGAGAAGTTAGCTGTCTCTAATGTAAATTTTGAAGTTTACCTTTAAGTGAAAAGGCTTAAATAATATAAAGGGACGATAAGACCCTATAAAACTTTACTTCTTATTAAATGGTGTAAATTTGCTCTTTAGTGTTTTGTTGGGGCGATAGGAATATAA-ATATTAACTGTTTTTAATAAAATAATGTAATTAGATTAAATGATCCTTTAATATGGATNATAAGATTAAGCTACTTTAGGGATAACAGCGTAATTTCTCTTGAGAGACCNAATCGACAGAGTTAGTTGCGACCTCGATGTTGAATTAAGATATCATTTAGGTGCAGAAGTCTAAATAGTAGGTCTTTTCGACCTTNAAAATCCT

11_Lysmata_rafa GGGCCGCGGTATACTGACCGTGCGAAGGTAGCATAGTAATTAGTTCTTTAATTGAGGACTGGAATGAACGGTGGACGAGAAGTTAACTGTCTCGGGTATAAATTTTGAAGTTTACTTTTAAGTGAAAAGGCTTAAATAATATAAAGGGACGATAAGACCCTATAAAACTTTACTCCTT-TTAAATAGTGTAAATTTGCTCTTCGGTGTTTTGTTGGGGCGATAAAAATATAA-ATATTAACTGT-TTTAATAAAATAATATAATTAGATTAGATGATCCTTTAATATGGATTACAAGACCAAGTTACTTTAGGGATAACAGCGTAATTTCTCTTGAGAGACCAAATCGACAGAGTTAGTTGCGACCTCGATGTTGAATTAAGATATCATTTAGGCGTAGAAGTCTAAATAGTAGGTCTGTTCGACCTTTAAAATCTT

12_Lysmata_wurdemanni GGGCCGCGGTATACTGACCGTGCGAAGGTAGCATAATAATTAGTTCTTTAATTGAGGACTGGAATGAACGGTGGACGAGAAGTTATCTGTCTCTAAGGTAGGTCTTGAATTTTACCTTTAAGTGAAAAGGCTTAAATAACATAAAGGGACGATAAGACCCTATAAAACTTTACTTTCTATTAACG--TGTAAATTTGTTATTGG-TGTTTTGTTGGGGCGGCAGGAATATAA-ATATTAACTGTTCTTATGAAA-TAATATAATTAGATTAATTGATCCTATACTATGGATTACAAGATTAAGTTACTTTAGGGATAACAGCGTAATTTCTCTTGAGAGACCCTATCGACAGAGTTAGTTGCGACCTCGATGTTGAATTAAGATACCGTTTAGATGCAGGAGTCTAAACAGTAGGTCTGTTCGACCTTTAAAATCTT

12_Lysmata_wurdemanni GGGCCGCGGTATACTGACCGTGCGAAGGTAGCATAATAATTAGTTCTTTAATTGAGGACTGGGATGAACGGTGGACGAGAAGTTATCTGTCTCTAAGGTAGGTCTTGAATTTTACCTTTAAGTGAAAAGGCTTAAATAACATAAAGGGACGATAAGACCCTATAAAACTTTACTTTCTATTAACG--TGTAAATTTGTTATTGG-TGTTTTGTTGGGGCGGCAGGAATATAA-ATATTAACTGTTCTTATGAAA-TAATATAATTAGATTAATTGATCCTATACTATGGATTACAAGATTAAGTTACTTTAGGGATAACAGCGTAATTTCTCTTGAGAGACCCTATCGACAGAGTTAGTTGCGACCTCGATGTTGAATTAAGATACCGTTTAGATGCAGGAGTCTAAACAGTAGGTCTGTTCGACCTTTAAAATCTT

14_Lysmata_wurdemanni gggccgcggtacactgaccgtgcgaaggtagcataataattagttctttaattgagggctggaatgaacggtgaacgagaagttagctgtctctaaggtaggtcttgaactttacctttaagtgaaaaggcttaaataatataaagggacgataagaccctataaaactttactttctattaatg--tgtaaatttgtttttgg-tgttctgttggggcgacaggaatataa-atactaactgttcttagaaaa-taatataattagatttgatgatcctttaatatggattacaagattaagttactttagggataacagcgtaatttctcttgagagaccttatcgacagagctagttgcgacctcgatgttgaattaagatgccgctcaggtgcaggagcctggggagtgggtctgttcgacctttaaaatctt

12_Lysmata_wurdemanni GGGCCGCGGTATACTGACCGTGCGAAGGTAGCATAATAATTAGTTCTTTAATTGAGGACTGGGATGAACGGTGGACGAGAAGTTATCTGTCTCTAAGGTAGGTCTTGAATTTTACCTTTAAGTGAAAAGGCTTAAATAACATAAAGGGACGATAAGACCCTATAAAACTTTACTTTCTATTAACG--TGTAAATTTGTTATTGG-TGTTTTGTTGGGGCGGCAGGAATATAA-ATATTAACTGTTCTTATGAAA-TAATATAATTAGATTAATTGATCCTATACTATGGATTACAAGATTAAGTTACTTTAGGGATAACAGCGTAATTTCTCTTGAGAGACCCTATCGACAGAGTTAGTTGCGACCTCGATGTTGAATTAAGATATCGTTTAGATGCAGGAGTCTAAACAGTAGGTCTGTTCGACCTTTAAAATCTT

13_Lysmata_wurdemanni GGGCCGCGGTATACTGACCGTGCGAAGGTAGCATAATAATTAGTTCTTTAATTGAGGGCTGGGATGAACGGTGGACGAGAAGTTATCTGTCTCTAAGGTAGGTTTTGAATTTTACCTTTAAGTGAAAAGGCTTAAATAATATAAAGGGACGATAAGACCCTATAAAACTTTACTTTCTTTTAAATCGTGTAAATTTGTTATTGG-TGTTTTGTTGGGGCGGCAGGAATATAA-ATATTAACTGTTCTTATGAAA-TAATATAATTAGATTAATTGATCCTTTAATATGGATTACAAGATTAAGTTACTTTAGGGATAACAGCGTAATTTCTCTTGAGAGACCTTATCGACAGAGTTAGTTGCGACCTCGATGTTGAATTAAGATACCGTTTAGGTGCAGGAGTCTAAACAGTAGGTCTGTTCGACCTTTAAAATCTT

15_Lysmata_gracilirostris GGGCCGCGGTATTCTGACCGTGCGAAGGTAGCATAGTAATTAGTTCTTTAATTGAGGACTGGAATGAACGGTGGACGAGAAGTTAGCTGTCTCTAAGGCAAGGCTTGAATTTTACTTTTAAGTGAAAAGGCTTAAATAAAATAAAGGGACGATAAGACCCTGTAAAACTTAACTTTCTATT--CTAGTGTAAGCTT--TTAAGG-TGTTTTGCTGGGGCGGCAGGAATATAATTAGTTAACTGTTCTTATAATAATA--ATAATTAGGTTAATTGATCCTCTAATAGGGATTAAAAGATTAAGTTACTTTAGGGATAACAGCGTAATTTCTCTTGAGAGACCAAATCGACAGAGTTAGTTGCGACCTCGATGTTGAATTAAGATATCATTCAGGTGCAGGAGTCTGAATAGTAGGTCTGTTCGACCTTTAAAATCTT

16_Lysmata_nayaritensis GGGCCGCGGTATCTTGACCGTGCTAAGGTAGCATAGTCATTAGTTCTTTAATTGAGGACTGGAATGAAAGGTGGATGAGAAGTTAGCTGTCTTTAAGACAAATCTTGAATTTTACTTCTGAGTGAAAAGGCTTAGATAAGATAAGGGGACGACAAGACCCTATAAAACTTTACAGCTT---ATCTAGTATCTAATTAAAG-GTG-TGTTTTGCTGGGGCGGCAGGAATATAA--AATTAACTGTTTCT-TTAAAATAGGTTAATTAG-TAAATTGATCCTTTAATGAGGATTAAGAGATTAAGTTACTTTAGGGATAACAGCGTAATCTCTCTTGAGAGCCCTGATCGACAGAGTTAGTTGCGACCTCGATGTTGAATTAAGATGTTACCTAGGTGCAGGAGTCTAGGCGGTGGGTCTGTTCGACCTTTAAAATCTT

42_Lysmata_cf._anchisteus GGGCCGCGGTATTTTGACCGTGCAAAGGTAGCATAATGAAATGTCTCTTAATTGGAGACTGGAATGAACGGTGGACAAGAAGTAAGCTGTCTCTTTAATAAAACTGAAATTTTACTTTTAAGTGAAAAGGCTTAAATAATATAAGAAGACGATAAGACCCTATAAAACTTTACAAATCAGTAAGTAGTGTAAAAG-GGTGTGTATTGTTTTGCTGGGGCGGCAGAGATATAA-AAG-TAACTGTTTTT-TCAAAATACCATATGTAG-TAAACTGGTCCTTTAATAAGGATTAAAAGATCAAGTTACTTTAGGGATAACAGCGTAATTTCTCTTGAGAGTTCTAATCGACAGAGCAAGTTGCGACCTCGATGTTGAATTAAGGTGTTATTCAGGTGCAGCCGCTTGAATAGTGAGTCTGTTCGACTTTTAAAACCTT

43_Lysmata_hochi GGGCCGCGGTATTTTGACCGTGCAAAGGTAGCATAATCAAATGTTTCTTAATTGGAAACCGGAATGAACGGTGGACGAGAAGTAAGCTGTCTCTTTAATAAAGCTTGAATTTTACTTTTAAGTGAAAAGGCTTAAATAACGTAAGAAGACGATAAGACCCTATAAAACTTTACAAGCT-----GTAGTGTAT-CTTAATTTAAGTTGTTTTGCTGGGGCGGCAGAGATAAAATGAG-TAACTGTTTTT-TGAAAATATTATGTATAG-AAAGTTGATCCTGTATTATGGATTAAAAGAGTAAGTTACTTTAGGGATAACAGCGTAATTTCTCTTGAGAGTTCTGATCGACAGAGTAAGTTGCGACCTCGATGTTGAATTAAGGTGTTATCCAGGTGCAGCAGCTTGGATAGTAGGTCTGTTCGACCTTTAAAACCCT

25_Lysmata_hochi GGGCCGCGGTAATTTGACCGTGCTAAGGTAGCATAATCAATAGTCCTTTAATTGGGGACCGGAATGAACGGTGGACAAGAAGTTAACTGTCTCTTAAATGAATCTTGAAGTTTACTTTTAAGTGAAAAGGCTTAAATGAGTTAAAGGGACGATAAGACCCTATAAAACTTAACAAGTTAATTA-T-GTTTAAAATGACTAAAGGTTGTTTCGTTGGGGCGATTGAGATAAAATTAATTAACTGTCTAAAATAAAA----ATAATTAG-TGAGTTGATCCTTTATTAAGGATTAAAAGATTAAGTTACTTTAGGGATAACAGCGTAATTTCTCTTGAGAGTCCTAATCGACAGAGTTAGTTGCGACCTCGATGTTGAATTAAGGTGTTATTCGGGCGCAGAAGCTCGAAAAGTAGGTCTGTTCGACCTTTAAAACCTT

**16S Additional Figure, S1A**

1_Lysmata_bahia TACTGACCGTGCAAAGGTAGCATAATAATTAGTCTTTAATTGAGGGCTGGAATGAAAGGTTTACGAGAAGGTAGCTGTCTCTTTTATAAAGTTCATTTAAGTGAGAAGGCTTAAATAAGATAAAGGGACGAGAAGACCCTATAAAACTTTATAGGGATTTAGATTAAGTTACTTTAGGGATAACAGCGTAATTTCTCTTGAGAGTCCAAATCGACAGAGTTAGTTGCGACCTCGATGTTGAATTAAGATGTTAGCTAGGTGCAGGAGCCTAGATAGTAGGTCTGTTCGACCTTTAAAATCTT

1_Lysmata_bahia TACTGACCGTGCAAAGGTAGCATAATAATTAGTCTTTAATTGAGGGCTGGAATGAAAGGTTTACGAGAAGGTAGCTGTCTCTTTTATAAAGTTCATTTAAGTGAGAAGGCTTAAATAAGATAAAGGGACGAGAAGACCCTATAAAACTTTATAGGGATTTAGATTAAGTTACTTTAGGGATAACAGCGTAATTTCTCTTGAGAGTCCAAATCGACAGAGTTAGTTGCGACCTCGATGTTGAATTAAGATGTTAGCTAGGTGCAGGAGCCTAGATAGTAGGTCTGTTCGACCTTTAAAATCTT

2_Lysmata_bahia TACTGACCGTGCAAAGGTAGCATAATAATTAGTCTTTAATTGAGGGCTGGAATGAAAGGTTTACGAGAAGGTAGCTGTCTCTTTTATAAAGTTCATTTAAGTGAGAAGGCTTAAATAAGATAAAGGGACGAGAAGACCCTATAAAACTTTATAGGGATTTAGATTAAGTTACTTTAGGGATAACAGCGTAATTTCTCTTGAGAGTCCAAATCGACAGAGTTAGTTGCGACCTCGATGTTGAATTAAGATGTTAGCTAGGTGCAGGAGCCTAGATAGTAGGTCTGTTCGACCTTTAAAATCTT

49_Alope_orientalis TTCTGACCGTGCGAAGGTAGCATCATCAATAGTTTTTAATTGGAGTCTGGAATGAACGATGGACAAAAAGTAATCTGTCTTGAGAGTAAAAATCTTTTAAGTGAGAAGGCTTAAATAAGTTAGAGGGACGATAAGACCCTATAAAACTTTATATAGATTAGTATAAAGTTCCTTTAGGGATAACAGCGTTATTCTTTCTGAGAGTTCTTATCGACGAAGGTAGTTGCGACCTCGATGTTGAATTAAAATTCCTTTTAGGTNNNNNNNNNNNNNNNNNNNNNNNNNNNNNNNNNNNNNNNNNN

44_Parhippolyte_mistica CAATAACCGTGCGAAGGTAGCATCATCATTTGTTTTTAATTGAAGGCTGGAATGAATGGTGAACGAGAAGTAAGCTGTCTCTTTAATGTATACCCTTTAAGTGAAAAGGCTTAAATGTCACGGGGGGACGATAAGACCCTATAAAGCTTTATAAGGATCATTATTAAGTTCCTTTAGGGATAACAGCGTTATTTCCCTTGAGAGTTCATATCGACAGGGTAAGTTGCGACCTCGATGTTGAATTAAGGTTCCATTCAGGTNNNNNNNNNNNNNNNNNNNNNNNNNNNNNNNNNNNNNNNNNN

50_Hippolyte acuta ACTTGACCGTGCTAAGGTAGCATNATCAGTAGTTTTTAATTGATGACTGGAATGAATGGCTTACGAGAAATAAGCTGTCTTAAAAATAAAAATCCTTCAAGTGAAAAGGCTTGAATACTACTAAGGGACGATAAGACCCTATAAAACTTAATAAGGATTAGAAAAAAGTTNCTTTAGGGATAACAGCGTAATTTTTTCAGAGAGTTCTTATCGAAGAAAGTAGTTGCGACCTCGATGTTGAATTAAAGTTNCTCTTAAGCNNNNNNNNNNNNNNNNNNNNNNNNNNNNNNNNNNNNNNNNNN

54_Heptacarpus_futilirostris ATCTGACCGTGCGAAGGTAGCATCATCAGTAGTTCTTAATTGGAGGCTGGAATGAATGGTGCACGAGAAGAAAGCTGTTTCTTCTTCAAGAATCTTTTAAGTGAAAAGGCTTAAATAAATTGAAGGGACGATAAGACCCTATAAAACTTTATAAAGATTAAAATTAAGTTCCTTTAGGGATAACAGCGTGATTTTTTTAAAGAGTTCTTATCGATGAAATTAGTTGCGACCTCGATGTTGAATTAAAATTCCTATAAAGTNNNNNNNNNNNNNNNNNNNNNNNNNNNNNNNNNNNNNNNNNN

56_Heptacarpus_palpator TCTTAACCGTGCGAAGGTAGCATAATCAATAGTTTTTAATTGGAGGCTGGAATGAATGGCGCACGAGAAGTTAGCTGTCTCTTTTTTAGAGATCTTTTAAGTGAAAAGGCTTAAATAAACTAAAGGGACGATAAGACCCTATAAAACTTTATAAAGATTAAAATTAAGTTACTTTAGGGATAACAGCGTAATTTTTTCTTAGAGTTCCTATCGACGAAATTAGTTGCGACCTCGATGTTGAATTAAAATTTCTATAAAGTGTAGCAGCTTACTTAGTTGGTCTGTTCGACCATTAAAATTTT

55_Heptacarpus_geniculatus TTTTGACCGTGCGAAGGTACAcMBATCAATAGTTCTTAATTAGAGGCTGGAATGAATGGTGCACGAGAAAGAAGCTGTCTCTTCTTTAAGAATCTTTTAAGTGAAAAGGCTTAAATAATCTAAGGGGACGATAAGACCCTATAAAACTTTATAAAGATTATAATTACAcMBCTTTAGGGATAACAGCGTAATTTTTTCTGAGAGTTCTTATCGATGAAATTAGTTGCGACCTCGATGTTGAATTAACAcMBCTTTTNNNNNNNNNNNNNNNNNNNNNNNNNNNNNNNNNNNNNNNNNNNNNN

59_Synalpheus_brevicarpus ATTTGACCGTGCAAAGGTAGCATAATCAATAGTTTTTAATTGGAGGCTTGGATGAAAGGTTGACGGATGAGGAGCTGTCTCTTTAGTTGTGTTCGTTTGTGTGAAAAGGCATTAATGGTTTAGGGGGACGATAAGACCCTATAAAACTTAATAGGGAGTTAAAGTAAGTTACTTTAGGGATAACAGCGTAATTTTTCTTGAGAGTTCTTATCGAAGGAAGTAGTTGTGACCTCGATGTTGAATTAAATTTTCCCTTTGGTGNNNNNNNNNNNNNNNNNNNNNNNNNNNNNNNNNNNNNNNNN

45_Barbouria_cubensis ACCTGACCGTGCGAAGGTAGCATAATAATTTGTTTTTAATTGAAGGCTCGTATGAATGGTGGACAAGAAGAAGGCTGTCTTATTAATGAAAGCCTTTTAAGTGAGAAGGCTTAAATAAACCAAGGGGACGATGAGACCCTATAAAACTTTATAAGGATTAAAAGTAAGTTACTTTAGGGATAACAGCGTTATTTCCCCTGAGAGGTCTTATCGACGGGGTAAGTTGCGACCTCGATGTTGAATTAAGGTTTCACCCAGACGNNNNNNNNNNNNNNNNNNNNNNNNNNNNNNNNNNNNNNNNN

39_Exhippolysmata_oplophoroides CTTTGACCGTGCAAAGGTAGCATAATCAGTAGTCTTTAATTGGGAACTTGTATGAAGGGCGGACAAGAAGGAACCTGTCTCTAAAATAAAATTCTTTTAAGTGAAAAGGCTTAAATAAAATAAGGGGACGATAAGACCCTATAAAACTTGATGGGGATTAAAATAAAGTTACTTTAGGGATAACAGCGTTATTTCTCTTGAGAGTCCAAATCGACAGAGTAAGTTGCGACCTCGATGTTGAATCAAGATGTTATTCAGGTGCAGAAGCCTGAATTGTGGGTCTGTTCGACCTTTAAAATCTT

39_Exhippolysmata_oplophoroides CTTTGACCGTGCAAAGGTAGCATAATCAGTAGTCTTTAATTGGGAACTTGTATGAAGGGCGGACAAGAAGGAACCTGTCTCTAAAATAAAATTCTTTTAAGTGAAAAGGCTTAAATAGAATAAGGGGACGATAAGACCCTATAAAACTTGATGGGGATTAAAATAAAGTTACTTTAGGGATAACAGCGTTATTTCTCTTGAGAGTCCAAATCGACAGAGTAAGTTGCGACCTCGATGTTGAATCAAGATGTTATTCAGGTGCAGAAGCCTGAATTGTGGGTCTGTTCGACCTTTAAAATCTT

39_Exhippolysmata_oplophoroides CTTTGACCGTGCAAAGGTAGCATAATCAGTAGTCTTTAATTGGGAACTTGTATGAAGGGCGGACAAGAAGGAACCTGTCTCTAAAATAAAATTCTTTTAAGTGAAAAGGCTTAAATAAAATAAGGGGACGATAAGACCCTATAAAACTTGATGGGGATTAAAATAAAGTTACTTTAGGGATAACAGCGTTATTTCTCTTGAGAGTCCAAATCGACAGAGTAAGTTGCGACCTCGATGTTGAATCAAGATGTTATTCAGGTGCAGAAGCCTGAATTGTGGGTCTGTTCGACCTTTAAAATCTT

40_Lysmatella_prima TTATGACCGTGCAAAGGTAGCATAGTCACTAGTCTTTAATTGGGTTCTGGAATGAATGGTGGACAAGAAGAAAACTGTCTAGAATATAAAATTCTTTTAAGTGAGAAGGCTTAAATAAAATAAGGGGACGATAAGACCCTATAAAACTTGTTAAGGATTTTGATAAAGTTACTTTAGGGATAACAGCGTTATTTCTCTTGAGAGTCCAAATCGACAGAGTAAGTTGCGACCTCGATGTTGAATCAAGATGTTATTCAGGTGGAGGAGCCTGAATTGTAGGTCTGTTCGACCTTTAAAATCTT

46_Merguia_rhizophorae TTTTGACCGTGCGAAGGTAGCATAATCAATAGTTTTTAATTGAAGGCTGGAATGAATGGTGGACGAGATGGAGGCTGTCTCTATTATAAATTTCTTTTAAGTGAAAAGGCTTAAATAATTTAGTGGGACGATAAGACCCTGTAAAGCTTTATTAGGATTATAATAAAGTTACTTCAGGGATAACAGCGTAATTTCTCTTGAGAGCACATATCGACAGAGTTAGTTGCGACCTCGATGTTGAATTAAGGTAATTGTTAGGCGCAGCAGTTTATAAAATAGGTCTGTTCGACCTTTAAAACCTT

48_Merguia_oligodon TTTTGACCGTGCGAAGGTAGCACAATCAATAGTTTTTAATTGAGGGCTGGAATGAATGGTGGACGAGATGGAGGCTGTCTCTAGCGTAAATCTCTTTTAAGTGAAAAGGCTTAAATGATTTAGTGGGACGATAAGACCCTGTAAAGCTTCATTAGGATTAAAATAAAGTCACTTCAGGGATAACAGCGTAATTTCTCTTGAGAGCACATATCGACAGAGTTAGTTGCGACCTCGATGTTGAATTAAGGTCATTTTTAGGNNNNNNNNNNNNNNNNNNNNNNNNNNNNNNNNNNNNNNNNNNN

57_Thor_amboinensis TTTTGACCGTGCGAAGGTAGCATAATCAGTTGTTTTTAATTGAAGGCTGGAATGAAAGGTGCACGAGAAAAAATCTGTATTAATTATAAGTTTCTTTTAAGTGAAAAGGCTTAAATATTTTAAAGGGACGATAAGACCCTATAAAGCTTTATAAAGATTAAAATAAAGTTACTTTAGGGATAACAGCGTAATTTTTTCAGAGAGTTCTTATCGAAGAAAGTAGTTGCGACCTCGATGTTGAATTAAAATTTCTATTAAATGTAGCAGTTTAAGTAGTTGGTCTGTTCGACCATTAAAGTTTT

57_Thor_amboinensis TTTTGACCGTGCGAAGGTAGCATAATCAGTTGTTTTTAATTGAAGGCTGGAATGAAAGGTGCACGAGAAAAAATCTGTATTAATTATAAGTTTCTTTTAAGTGAAAAGGCTTAAATATTTTAAAGGGACGATAAGACCCTATAAAGCTTTATAAAGATTAAAATAAAGTTACTTTAGGGATAACAGCGTAATTTTTTCAGAGAGTTCTTATCGAAGAAAGTAGTTGCGACCTCGATGTTGAATTAAAATTTCTATTAAATGTAGCAGTTTAAGTAGTTGGTCTGTTCGACCATTAAAGTTTT

58_Thor_cf._manningi TTTTGACTGTGCAAAGGTAGCATAATCAGTTGTTTTTAATTGGAGGCTGGAATGAAAGGTAAACGAGAAAAAAACTGTCTCAGCTTAAAGACTCCTTTAAGTGAAAAGGCTTAAATAATTTAAAGGGACGATAAGACCCTATAAAACTTTTTAAGGATTAAAATAAAGTTACTTTAGGGATAACAGCGTTATTTTCTTGGAGAGTTCATATTGATAAGATAAGTTGCGACCTCGATGTTGAATTAAGGTGTCTTGTAAATGTAGCAGTTTATTAAGTTGGTCTGTTCGACCATTAAAACCTT

53_Tozeuma_carolinense ATTTGACCGTGCGAAGGTAGCATAATCAGTAGTTCTTAATTGGGGGCTTGTATGAAAGGTGGACGAGAGGAAAGCTGTCTTTGTGATGGATCTCTTTTAAGTGAAAAGTCTTAAATATACTTAAGGGACGATAAGACCCTGTAAAACTTAATAAGGATTAAAATAAAGTTACTTCAGGGATAACAGCGTGATTTTTTTTGAGAGTCCTTATCGACAAAAGTAGTTGCGACCTCGATGTTGAATTAAAATTTCTTTCAAATGCAGCAGTTTGATTAGTGGGTCTGTTCGACCTTTAAAATTTT

51_Hippolyte_williamsi ATTTGACCGTGCTAAGGTAGCATAATCAATAGTTTCTAATTTAAAACTGGAATGAATGGTTGACGAAAAGCAAGCTTTTTTTAAAATATAAATCTTCTGAGTGAAAAGGCTTAGATTCAGATAAGGGACGATAAGACCCTATAAAACTTAAAAAGATTTAAAACTAAGTTACTTTAGGGATAACAGCGTAATTTTTTCTGAGAGTTCTTATCGAAGAAAATAGTTGCGACCTCGATGTTGAATTAAAATTTTTGATTAACGCAGATGTTAAAGAAATAGGTCTGTTCGACCTTTAAAATTTT

52_Hippolyte_inermis CTTTGACCGTGCTAAGGTAGCATAATCAATAGTTCCTAACTAGGGACCGGAATGAACGGTTGACGAAAAGAAAGCTGTCTCTGAGGCATAGATCTCTTAAGTGAAAAGGCTTAAATATTGTTAAGGGACGATAAGACCCTGTGAAACTTAATAAAGATTAGAACAAAGTTACTTCAGGGATAACAGCGTAATTTTTTCTGAGAGTTCATATCGAAGAAAGTAGTTGCGACCTCGATGTTGAATTAAAGTTTTTAGTAAGCGCAGCGGCTTAAAAAATAGGTCTGTTCGACCTTAAAAATTTT

41_Lysmata_lipkei TTTTGACCGTGCAAAGGTAGCATAATCAATAGTTTTTAATTGAAAACTGGAATGAAAGGTGGACAAGAAGTAGACTGTCTCTTTTATAAGACTCTTTTAAGTGAAAAGGCTTAAATGGTTTAGGGGGACGATAAGACCCTATAAAACTTAATAGGGATTAGGATAAAGTTACTTTAGGGATAACAGCGTAATCTCTCTTGAGAGTCCTAATCGACAGAGTAAGTTGCGACCTCGATGTTGAATTAAGGTGTTATCTAGGCGCAGAAGTCTAGACGGTAGGTCTGTTCGACCTTTAAAACCTT

26_Lysmata_acicula TCCTGACCGTGCGAAGGTAGCATAATCAATAGTTTTTAATTGAAGACTGGAATGAAGGGTGGACAAGGGGTAAGCTGTCTCTAAAATAAATCTCCTTTAAGTGAAAAGGCTTAAATAAAGTAAAGGGACGATAAGACCCTATAAAACTTAATAAGGATTATAATTAAGTTACTTTAGGGATAACAGCGTAATTTCTCTTGAGAGTCCTAATCGACAGAGTTAGTTGCGACCTCGATGTTGAATTAAGATGTTATTCAGGCGCAGCAGTNNNNNNNNNNNNNNNNNNNNNNNNNNNNNNNNNN

27_Lysmata_cf._trisetacea TCCTGACCGTGCGAAGGTAGCATAATCAATAGTTTTTAATTGAAGACTGGAATGAAGGGTGGACAAGGGGTTATCTGTCTCTTAGATAAATCTCTTTTAAGTGAAAAGGCTTAAATGAAGTAAGGGGACGATAAGACCCTATAAAACTTAATAAGGATTATAATTAAGTTACTTTAGGGATAACAGCGTAATTTCTCTTGAGAGTTNTAATCGACAGAGCTAGTTGCGACCTCGATGTTGAATTAAGATGTTATTCAGGCGCAGCAGTNNNNNNNNNNNNNNNNNNNNNNNNNNNNNNNNNN

28_Lysmata_galapagensis 2 TACTGACCGTGCGAAGGTAGCATAATCAATAGTTTTTAATTGAAGACTGGAATGAATGGTGGACAAGGGGTTTGCTGTCTCTAAGGTAAATCTCCTTTAAGTGAAAAGGCTTAAATGGCGTAAAGGGACGATAAGACCCTATAAAACTTGATAGGGATTACGATTAAGTTACTTTAGGGATAACAGCGTAATTTCTCTTGAGAGTTCTAATCGACAGAGTTAGTTGCGACCTCGATGTTGAATTAAGATGTTATTCAGGCGCAGAAGTCTGAAGGGTAGGTCTGTTCGACCTTTAAAATCTT

31_Lysmata_nilita TACTGACCGTGCGAAGGTAGCATAATCAATAGTTTTTAATTGAAGACCGGAATGAATGGTGGACAAGGGGTTAGCTGTCTCTAAGGTAAGTCTCCTTTAAGTGAAAAGGCTTAAATGACGTAAAGGGACGATAAGACCCTATAAAACTTGATAGGGATTATGATTAAGTTACTTTAGGGATAACAGCGTAATTTCTCTTGAGAGTTCTAATCGACAGAGTTAGTTGCGACCTCGATGTTGAATTAAGATGTTATTCAGGCGCAGGAGTCTGAAGGGTAGGTCTGTTCGACCTTTAAAATCTT

29_Lysmata_moorei TACTGACCGTGCGAAGGTAGCATAATCAATAGTTTTTAATTGAAGACTGGAATGAAGGGTGGACAAGGGGCTTGCTGTCTCTAAGGTAAATCTCTTTTAAGTGAAAAGGCTTAAATGGCGTAGAGGGACGATAAGACCCTATAAAACTTAATAGGGATTATGATCAAGTTACTTTAGGGATAACAGCGTAATTTCTCTTGAGAGTTCTAATCGACAGAGTTAGTTGCGACCTCGATGTTGAATTAAGATGTTATTCAGGCGCAGAAGTCTGAATGGTAGGTCTGTTCGACCTTTAAAATCTT

30_Lysmata_moorei TACTGACCGTGCGAAGGTAGCATAATCAATAGTTTTTAATTGAAGACTGGAATGAAGGGTGGACAAGGGGCTTGCTGTCTCTAAGGTAAATCTCTTTTAAGTGAAAAGGCTTAAATGGCGTAGAGGGACGATAAGACCCTATAAAACTTAATAGGGATTATGATCAAGTTACTTTAGGGATAACAGCGTAATTTCTCTTGAGAGTTCTAATCGACAGAGTTAGTTGCGACCTCGATGTTGAATTAAGATGTTATTCAGGCGCAGAAGTCTGAATGGTAGGTCTGTTCGACCTTTAAAATCTT

32_Lysmata_intermedia TACTGACCGTGCGAAGGTAGCATAATCAATAGTTTTTAATTGGAGACTGGAATGAACGGTGGACAAAGGGTTTACTGTCTCTGAAATGAATCTCTTTTAAGTGAAAAGGCTTAAATGACGTAAAGGGACGATAAGACCCTATAAAACTTGATAGGGATTAAGATTAAGTTACTTTAGGGATAACAGCGTAATTTCTCTTGAGAGTTCTTATCGACAGAGTTAGTTGCGACCTCGATGTTGAATTAAGATGTTATTTGGGCGCAGGAGTCCAAAAAGTAGGTCTGTTCGACCTTTAAAATCTT

33_Lysmata_intermedia TACTGACCGTGCGAAGGTAGCATAATCAATAGTTTTTAATTGGAGACTGGAATGAACGGTGGACAAAGGGTTTACTGTCTCTGAAATGAATCTCTTTTAAGTGAAAAGGCTTAAATGACGTAAAGGGACGATAAGACCCTATAAAACTTGATAGGGATTAAGATTAAGTTACTTTAGGGATAACAGCGTAATTTCTCTTGAGAGTTCTTATCGACAGAGTTAGTTGCGACCTCGATGTTGAATTAAGATGTTATTTGGGCGCAGGAGTCCGAAAAGTAGGTCTGTTCGACCTTTAAAATCTT

32_Lysmata_intermedia TACTGACCGTGCGAAGGTAGCATAATCAATAGTTTTTAATTGGAGACTGGAATGAACGGTGGACAAAGGGTTTACTGTCTCTGAAATGAATCTCTTTTAAGTGAAAAGGCTTAAATGACGTAAAGGGACGATAAGACCCTATAAAACTTGATAGGGATTAAGATTAAGTTACTTTAGGGATAACAGCGTAATTTCTCTTGAGAGTTCTTATCGACAGAGTTAGTTGCGACCTCGATGTTGAATTAAGATGTTATTTGGGCGCAGGAGTCCAAAAAGTAGGTCTGTTCGACCTTTAAAATCTT

34_Lysmata_cf_intermedia TACTGACCGTGCGAAGGTAGCATAATCAATAGTTTTTAATTGGAGACTGGAATGAACGGTGGACAAAGGGTTTACTGTCTCTGAAATGAATCTCTTTTAAGTGAAAAGGCTTAAATGACGTAAGGGGACGATAAGACCCTATAAAACTTGATAGGGATTAAGATTAAGTTACTTTAGGGATAACAGCGTAATTTCTCTTGAGAGTTCTTATCGACAGAGTTAGTTGCGACCTCGATGTTGAATTAAGATGTTATTCGGGCGCAGGAGTCTGAAAAGTAGGTCTGTTCGACCTTTAAAATCTT

35_Lysmata_cf_intermedia TACTGACCGTGCGAAGGTAGCATAATCAATAGTTTTTAATTGAAGACTGGAATGAACGGTGGACAAGGGGTTTACTGTCTCTAAAATGAATCTCTTTTAAGTGAGAAGGCTTAAATAATGTGGGGGGACGATAAGACCCTATAAAACTTGATAAGGATTAAGATTAAGTTACTTTAGGGATAACAGCGTAATTTCTCTTGAGAGTTCTAATCGACGGAGTTAGTTGCGACCTCGATGTTGAATTAAGATGTTATTTAGGTGCAGGAGTCTAAAGAGTAGGTCTGTTCGACCTTTAAAATCTT

36_Lysmata_holthuisi TATTGACCGTGCGAAGGTAGCATAATCAATAGTTTTTAATTGGAGACTGGAATGAACGGTGGACAAGGGGTTTACTGTCTCTGTGATGGATCTCTTTTAAGTGAAAAGGCTTAAATAATGTAAAGGGACGATAAGACCCTATAAAACTTGATAAGGATTAGGATTAAGTTACTTTAGGGATAACAGCGTGATTTCTCTTGAGAGTTCTAATCGACAGAGTTAGTTGCGACCTCGATGTTGAATTAAGGTGTTATTCAGGCGCAGGAGTCTGAAGAGTAGGTCTGTTCGACCTTTAAAATCTT

37_Lysmata_seticaudata TACTGACCGTGCGAAGGTAGCATAATCAATAGTTTTTAATTGAAGACTGGAATGAAGGGTGGACAAGGGGTTGGCTGTCTCTTAAATAAAATTCTTTTAAGTGAAAAGGCTTAAATAAAGTAGAGGGACGATAAGACCCTATAAAACTTTATAAGGATTAAGATTAAGTTACTTTAGGGATAACAGCGTAATTTCTCTTGAGAGTTCTAATCGACAGAGTTAGTTGCGACCTCGATGTTGAATTAAGATGTTACTCAGGTGCAGCAGTCTGAGTGGTAGGTCTGTTCGACCTTTAAAATCTT

37_Lysmata_seticaudata TACTGACCGTGCGAAGGTAGCATAATCAATAGTTTTTAATTGAAGACTGGAATGAAGGGTGGACAAGGGGTTGGCTGTCTCTTAAATAAAATTCTTTTAAGTGAAAAGGCTTAAATAAAGTAGAGGGACGATAAGACCCTATAAAACTTTATAAGGATTAAGATTAAGTTACTTTAGGGATAACAGCGTAATTTCTCTTGAGAGTTCTAATCGACAGAGTTAGTTGCGACCTCGATGTTGAATTAAGATGTTACTCAGGTGCAGCAGTCTGAGTGGTAGGTCTGTTCGACCTTTAAAATCTT

38_Lysmata_ternatensis TCCTGACCGTGCGAAGGTAGCATAATCAATAGTTTTTAATTGAAGACTGGAATGAAGGGTGGACAAGGGGTTAGCTGTCTCTAAATAAAATCTCTTTTAAGTGAAAAGGCTTAAATAACGTGAGGGGACGATAAGACCCTATAAAACTTAATAAGGATTACAATTAAGTTACTTTAGGGATAACAGCGTAATTTCTCTTGAGAGTCCAAATCGACAGAGTTAGTTGCGACCTCGATGTTGAATTAAGATGTTATTCAGGCGCAGCCGTCTGAATAGTGGGTCTGTTCGACCTTTAAAATCTT

38_Lysmata_ternatensis TCCTGACCGTGCGAAGGTAGCATAATCAATAGTTTTTAATTGAAGACTGGAATGAAGGGTGGACAAGGGGTTAGCTGTCTCTAGATAAAATCTCTTTTAAGTGAAAAGGCTTAAATAACGTGAGGGGACGATAAGACCCTATAAAACTTAATAAGGATTACAATTAAGTTACTTTAGGGATAACAGCGTAATTTCTCTTGAGAGTCCAAATCGACAGAGTTAGTTGCGACCTCGATGTTGAATTAAGATGTTATTCAGGCGCAGCCGTCTGAATAGTGGGTCTGTTCGACCTTTAAAATCTT

27_Lysmata_cf._trisetacea TCCTGACCGTGCGAAGGTAGCATAATCAATAGTTTTTAATTGAAGACTGGAATGAAGGGTGGACAAGGGGTTATCTGTCTCTTAGATAAATCTCTTTTAAGTGAAAAGGCTTAAATGAAGTAAGGGGACGATAAGACCCTATAAAACTTAATAAGGATTATAATTAAGTTACTTTAGGGATAACAGCGTAATTTCTCTTGAGAGTTCTAATCGACAGAGCTAGTTGCGACCTCGATGTTGAATTAAGATGTTATTCAGGCGCAGCAGTCTGAATGGTAGGTCTGTTCGACCTTTAAAATCTT

27_Lysmata_cf._trisetacea TCCTGACCGTGCGAAGGTAGCATAATCAATAGTTTTTAATTGAAGACTGGAATGAAGGGTGGACAAGGGGTTATCTGTCTCTTAGATAAATCTCTTTTAAGTGAAAAGGCTTAAATGAAGTAAGGGGACGATAAGACCCTATAAAACTTAATAAGGATTATAATTAAGTTACTTTAGGGATAACAGCGTAATTTCTCTTGAGAGTTCTAATCGACAGAGCTAGTTGCGACCTCGATGTTGAATTAAGATGTTATTCAGGCGCAGCAGTCTGAATGGTAGGTCTGTTCGACCTTTAAAATCTT

27_Lysmata_cf._trisetacea TCCTGACCGTGCGAAGGTAGCATAATCAATAGTTTTTAATTGAAGACTGGAATGAAGGGTGGACAAGGGGTTATCTGTCTCTTAGATAAATCTCTTTTAAGTGAAAAGGCTTAAATGAAGTAAGGGGACGATAAGACCCTATAAAACTTAATAAGGATTATAATTAAGTTACTTTAGGGATAACAGCGTAATTTCTCTTGAGAGTTCTAATCGACAGAGCTAGTTGCGACCTCGATGTTGAATTAAGATGTTATTCAGGCGCAGCAGTCTGAATGGTAGGTCTGTTCGACCTTTAAAATCTT

17_Lysmata_amboinensis TACTGACCGTGCGAAGGTAGCATAATCAATAGTCTTTAATTGAGGACTGGAATGAAGGGTGGACGAGAAGTTAGCTGTCTCCAAGACAAGTCTCTTTTAAGTGAAAAGGCTTAAATGAGATAAAGGGACGATAAGACCCTATAAAACTTTATAAGGATTAGAATTAAGTTACTTTAGGGATAACAGCGTAATTTCTCTTGAGAGTTCTAATCGACAGAGTTAGTTGCGACCTCGATGTTGAATTAAGGTGTTAGCTAGGCGCAGAAGCTTAGATAGTAGGTCTGTTCGACCTTTAAAACCTT

17_Lysmata_amboinensis TACTGACCGTGCGAAGGTAGCATAATCAATAGTCTTTAATTGAGGACTGGAATGAAGGGTGGACGAGAAGTTAGCTGTCTCCAAGACAAGTCTCTTTTAAGTGAAAAGGCTTAAATGAGATAAAGGGACGATAAGACCCTATAAAACTTTATAAGGATTAGAATTAAGTTACTTTAGGGATAACAGCGTAATTTCTCTTGAGAGTTCTAATCGACAGAGTTAGTTGCGACCTCGATGTTGAATTAAGGTGTTAGCTAGGCGCAGAAGCTTAGATAGTAGGTCTGTTCGACCTTTAAAACCTT

18_Lysmata_amboinensis TACTGACCGTGCGAAGGTAGCATAATCAATAGTCTTTAATTGAGGACTGGAATGAAGGGTGGACGAGAAGTTAGCTGTCTCCAAGACAAGTCTCTTTTAAGTGAAAAGGCTTAAATAAAATAAAGGGACGATAAGACCCTATAAAACTTTATAAGGATTAANATTAAGTTACTTTAGGGATAACAGCGTAATTTCTCTTGAGAGTTCTAATCGACAGAGTTAGTTGCGACCTCGATGTTGAATTAAGGTGTTAGCTAGGCGCAGAGACTTAGATAGTAGGTCTGTTCGACCTTTAAAACCTT

19_Lysmata_grabhami TACTGACCGTGCGAAGGTAGCATAATCAATAGTCTTTAATTGAGGACTGGAATGAAGGGTGGACGAGAAGTAAGCTGTCTCCAAGGCAAATCTCTTTTAAGTGAGAAGGCTTAAATAAGATAAAGGGACGATAAGACCCTATAAAACTTTATAAGGATTAGAATTAAGTTACTTTAGGGATAACAGCGTAATTTCTCTTGAGAGTTCTAATCGACAGAGTTAGTTGCGACCTCGATGTTGAATTAAGGTGTTAGCTAGGCGCAGAAGCTTAGATAGTAGGTCTGTTCGACCTTTAAAACCTT

19_Lysmata_grabhami TACTGACCGTGCGAAGGTAGCATAATCAATAGTCTTTAATTGAGGACTGGAATGAAGGGTGGACGAGAAGTAAGCTGTCTCCAAGGCAAATCTCTTTTAAGTGAGAAGGCTTAAATAAGATAAAGGGACGATAAGACCCTATAAAACTTTATAAGGATTAGAATTAAGTTACTTTAGGGATAACAGCGTAATTTCTCTTGAGAGTTCTAATCGACAGAGTTAGTTGCGACCTCGATGTTGAATTAAGGTGTTAGCTAGGCGCAGAAGCTTAGATAGTAGGTCTGTTCGACCTTTAAAACCTT

19_Lysmata_grabhami TACTGACCGTGCGAAGGTAGCATAATCAATAGTCTTTAATTGAGGACTGGAATGAAGGGTGGACGAGAAGTAAGCTGTCTCCAAGGCAAATCTCTTTTAAGTGAGAAGGCTTAAATAAGATAAAGGGACGATAAGACCCTATAAAACTTTATAAGGATTAGAATTAAGTTACTTTAGGGATAACAGCGTAATTTCTCTTGAGAGTTCTAATCGACAGAGTTAGTTGCGACCTCGATGTTGAATTAAGGTGTTAGCTAGGCGCAGAAGCTTAGATAGTAGGTCTGTTCGACCTTTAAAACCTT

20_Lysmata_grabhami TACTGACCGTGCGAAGGTAGCATAATCAATAGTCTTTAATTGAGGACTGGAATGAAGGGTGGACGAGAAGTAAGCTGTCTCCAAGGCAAATCTCTTTTAAGTGAGAAGGCTTAAATAAGATAAAGGGACGATAAGACCCTATAAAACTTTATAAGGATTAGAATTAAGTTACTTTAGGGATAACAGCGTAATTTCTCTTGAGAGTTCTAATCGACAGAGTTAGTTGCGACCTCGATGTTGAATTAAGGTGTTAGCTAGGCGCAGAAGCTTAGATAGTAGGTCTGTTCGACCTTTAAAACCTT

21_Lysmata_grabhami TACTGACCGTGCGAAGGTAGCATAATCAATAGTCTTTAATTGAGGACTGGAATGAAGGGTGGACGAGAAGTAAGCTGTCTCCAAGACAAATCTCTTTTAAGTGAAAAGGCTTAAATAAGATAAAGGGACGATAAGACCCTATAAAACTTTATAAGGATTANAATTAAGTTACTTTAGGGATAACAGCGTAATTTCTCTTGAGAGTTCTAATCGACAGAGTTAGTTGCGACCTCGATGTTGAATTAAGGTGTTAGCTAGGCGCAGAAGCTTAGATAGTAGGTCTGTTCGACCTTTAAAACCTT

22_Lysmata_debelius TATTGACCGTGCGAAGGTAGCATAATCAATAGTCTTTAATTGAGGACTGGAATGAAGGGTGGACGAGAAGTAAGCTGTCTCTAAAATAAATCTCTTTTAAGTGAAAAGGCTTAAATAAGATAAAGGGACGATAAGACCCTATAAAACTTTATAAGGATTAAAATTAAGTTACTTTAGGGATAACAGCGTAATTTCTCTTGAGAGTTCTAATCGACAGAGTTAGTTGCGACCTCGATGTTGAATTAAGGTGTTAGCTAGGCGCAGAAGCTTAGATAGTAGGTCTGTTCGACCTTTAAAACCTT

23_Lysmata_debelius TATTGACCGTGCGAAGGTAGCATAATCAATAGTCTTTAATTGAGGACTGGAATGAAGGGTGGACGAGAAGTAAGCTGTCTCTAAAATAAATCTCTTTTAAGTGAAAAGGCTTAAATAAGATAAAGGGACGATAAGACCCTATAAAACTTTATAAGGATTAAAATTAAGTTACTTTAGGGATAACAGCGTAATTTCTCTTGAGAGTTCTAATCGACAGAGTTAGTTGCGACCTCGATGTTGAATTAAGGTGTTAGCTAGGCGCAGAAGCTTAGATAGTAGGTCTGTTCGACCTTTAAAACCTT

22_Lysmata_debelius TATTGACCGTGCGAAGGTAGCATAATCAATAGTCTTTAATTGGGGACTGGAATGAAGGGTGGACGAGAAGTAAGCTGTCTCTAAAATAAATCTCTTTTAAGTGAAAAGGCTTAAATAAGATAAAGGGACGATAAGACCCTATAAAACTTTATAAGGATTATAATTAAGTTACTTTAGGGATAACAGCGTAATTTCTCTTGAGAGTTCTAATCGACAGAGTTAGTTGCGACCTCGATGTTGAATTAAGGTGTTAGCTAGGCGCAGAAGCTTAGATAGTAGGTCTGTTCGACCTTTAAAACCTT

24_Lysmata_californica TACTGACCGTGCGAAGGTAGCATAATCATTAGTCTTTAATTGAGGACTGGAATGAAAGGTGGACGAGAAGTTAACTGTCTCTAAAATAAATCTCTTTTAAGTGAAAAGGCTTAAATAAAATAAAGGGACGATAAGACCCTATAAAACTTTATAAGGATTAAAATTAAGTTACTTTAGGGATAACAGCGTAATTTCTCTTGAGAGTTCCAATCGACAGAGTTAGTTGCGACCTCGATGTTGAATTAAGATGTTAGTTAGGCGCAGAAGCTTAAGTAGTAGGTCTGTTCGACCTTTAAAATCTT

24_Lysmata_californica TACTGACCGTGCGAAGGTATCATAATCATTAGTCTTTAATTGAGGACTGGAATGAAAGGTGGACGAGAAGTTAACTGTCTCTAAAATAAATCTCTTTTAAGTGAAAAGGCTTAAATGAAATAAAGGGACGATAAGACCCTATAAAACTTTATAAGGATTAAAATTAAGTTACTTTAGGGATAACAGCGTAATTTCTCTTGAGAGTTCTGATCGACAGAGTTAGTTGCGACCTCGATGTTGAATTAAGATGTTAGTTAGGCGCAGAAGCTTAAGTAGTAGGTCTGTTCGACCTTTAAAATCTT

3_Lysmata_ankeri CACTGACCGTGCAAAGGTAGCATAGTAATTAGTCTTTAATTGAGGACTGGGATGAACGGTGGACGAGAAGTTGGCTGTCTCTAGTATAAGTTTCTTTTAAGTGAAAAGGCTTAAATAAAATAAAGGGACGATAAGACCCTATAAAACTTTATATGGAGTACGATTAAGTTACTTTAGGGATAACAGCGTAATTTCTCTTGAGAGTTCTAATCGACAGAGTTAGTTGCGACCTCGATGTTGAATTAAGATATCACTTAGGTGGAGGAGCTTAAGTAGTAGGTCTGTTCGACCTTTAAAATCTT

3_Lysmata_ankeri CACTGACCGTGCAAAGGTAGCATAGTAATTAGTCTTTAATTGAGGACTGGGATGAACGGTGGACGAGAAGTTGGCTGTCTCTAGTATAAGTTTCTTTTAAGTGAAAAGGCTTAAATAAAATAAAGGGACGATAAGACCCTATAAAACTTTATATGGAGTACGATTAAGTTACTTTAGGGATAACAGCGTAATTTCTCTTGAGAGTTCTAATCGACAGAGTTAGTTGCGACCTCGATGTTGAATTAAGATATCACTTAGGTGGAGGAGCTTAAGTAGTAGGTCTGTTCGACCTTTAAAATCTT

3_Lysmata_ankeri CACTGACCGTGCAAAGGTAGCATAGTAATTAGTCTTTAATTGAGGACTGGGATGAACGGTGGACGAGAAGTTGGCTGTCTCTAGTATAAGTTTCTTTTAAGTGAAAAGGCTTAAATAAAATAAAGGGACGATAAGACCCTATAAAACTTTATATGGAGTACGATTAAGTTACTTTAGGGATAACAGCGTAATTTCTCTTGAGAGTTCTAATCGACAGAGTTAGTTGCGACCTCGATGTTGAATTAAGATATCACTTAGGTGGAGGAGCTTAAGTAGTAGGTCTGTTCGACCTTTAAAATCTT

3_Lysmata_ankeri CACTGACCGTGCAAAGGTAGCATAGTAATTAGTCTTTAATTGAGGACTGGGATGAACGGTGGACGAGAAGTTGGCTGTCTCTAGTATAAGTTTCTTTTAAGTGAAAAGGCTTAAATAAAATAAAGGGACGATAAGACCCTATAAAACTTTATATGGAGTACGATTAAGTTACTTTAGGGATAACAGCGTAATTTCTCTTGAGAGTTCTAATCGACAGAGTTAGTTGCGACCTCGATGTTGAATTAAGATATCACTTAGGTGGAGGAGCTTAAGTAGTAGGTCTGTTCGACCTTTAAAATCTT

4_Lysmata_ankeri CCCTGACCGTGCAAAGGTAGCATAGTAATTAGTCTTTAATTGAGGACTGGGATGAACGGTGGACGAGAAGTTGGCTGTCTCTAATATAAATTTCTTTTAAGTGAAAAGGCTTAAATAAAATAAAGGGACGATAAGACCCTGTAAAACTTTATATGGACTACGATTAAGTTACTTTAGGGATAACAGCGTAATTTCTCTTGAGAGTCCTAATCGACAGAGTTAGTTGCGACCTCGATGTTGAATTAAGATATCACCTAGGCGGAGGAGCTTAGGTAGTAGGTCTGTTCGACCTTTAAAATCTT

5_Lysmata_pederseni TACTGACCGTGCAAAGGTAGCATAATAATTAGTCTTTAATTGAGGACTGGGATGAACGGTGGACGAGAAGTTAGCTGTCTCTAATATAAACTTCCTATAAGTGAAAAGGCTTATATATAATAAAGGGACGATAAGACCCTATAAAACTTTATAAGGACTACAATTAAGTTACTTTAGGGATAACAGCGTAATTTCTCTTGAGAGACCTAATCGACAGAGTTAGTTGCGACCTCGATGTTGAATTAAGATGTTACTTAGGCGGAGGAGCTTAAGTAGTAGGTCTGTTCGACCTTTAAAATCTT

8_Lysmata_pederseni TACTGACCGTGCAAAGGTAGCATAATAATTAGTCTTTAATTGAGGACTGGGATGAACGGTGGACGAGAAGTTAGCTGTCTCTAATATAAACTTCCTATAAGTGAAAAGGCTTATATATAATAAAGGGACGATAAGACCCTATAAAACTTTATAAGGACTACAATTAAGTTACTTTAGGGATAACAGCGTAATTTCTCTTGAGAGACCTAATCGACAGAGTTAGTTGCGACCTCGATGTTGAATTAAGATGTCACTTAGGCGGAGGAGCTTAAGTAGTAGGTCTGTTCGACCTTTAAAATCTT

6_Lysmata_pederseni TACTGACCGTGCAAAGGTAGCATAATCATTAGTCTTTAATTGAGGACTGGGATGAACGGTGGACGAGAAGTTAGCTGTCTCTAATATAAACTTCCTTTAAGTGAAAAGGCTTATATATAATAAAGGGACGATAAGACCCTATAAAACTTTATAAGGACTACAATTAAGTTACTTTAGGGATAACAGCGTAATTTCTCTTGAGAGACCTAATCGACAGAGTTAGTTGCGACCTCGATGTTGAATTAAGATGTTACTTAGGCGGAGGAGCTTAAGTAGTAGGTCTGTTCGACCTTTAAAATCTT

7_Lysmata_pederseni TACTGACCGTGCAAAGGTAGCATAATCATTAGTCTTTAATTGAGGACTGGGATGAACGGTGGACGAGAAGTTAGCTGTCTCTAATATAAACTTCCTTTAAGTGAAAAGGCTTATATATAATAAAGGGACGATAAGACCCTATAAAACTTTATAAGGACTACAATTAAGTTACTTTAGGGATAACAGCGTAATTTCTCTTGAGAGACCTAATCGACAGAGTTAGTTGCGACCTCGATGTTGAATTAAGATGTTACTTAGGCGGAGGAGCTTAAGTAGTAGGTCTGTTCGACCTTTAAAATCTT

9_Lysmata_boggessi CTCTGACCGTGCAAAGGTAGCATAGTAATTAGTCTTTAATTGAGGACTGGAATGAACGGTGGACGAGAAGTTAGCTGTCTCTAATGTAAATCTCTTTTAAGTGAAAAGGCTTAAATAATATAAAGGGACGATAAGACCCTATAAAACTTTATATGGATTATAATTAAGTTACTTTAGGGATAACAGCGTAATTTCTCTTGAGAGACCTAATCGACAGAGTTAGTTGCGACCTCGATGTTGAATTAAGATATCATTTAGGTGTAGAAGTCTAAATAGTAGGTCTGTTCGACCTTTAAAATCTT

10_Lysmata_rafa CACTGACCGTGCGAAGGTAGCATAGTAATTAGTCTTTAATTGAGGACTGGAATGAACGGTGGACGAGAAGTTAGCTGTCTCTAATGTAAATTTCCTTTAAGTGAAAAGGCTTAAATAATATAAAGGGACGATAAGACCCTATAAAACTTTATATGGATNATAATTAAGCTACTTTAGGGATAACAGCGTAATTTCTCTTGAGAGACCNAATCGACAGAGTTAGTTGCGACCTCGATGTTGAATTAAGATATCATTTAGGTGCAGAAGTCTAAATAGTAGGTCTTTTCGACCTTNAAAATCCT

11_Lysmata_rafa TACTGACCGTGCGAAGGTAGCATAGTAATTAGTCTTTAATTGAGGACTGGAATGAACGGTGGACGAGAAGTTAACTGTCTCGGGTATAAATTTCTTTTAAGTGAAAAGGCTTAAATAATATAAAGGGACGATAAGACCCTATAAAACTTTATATGGATTACAACCAAGTTACTTTAGGGATAACAGCGTAATTTCTCTTGAGAGACCAAATCGACAGAGTTAGTTGCGACCTCGATGTTGAATTAAGATATCATTTAGGCGTAGAAGTCTAAATAGTAGGTCTGTTCGACCTTTAAAATCTT

12_Lysmata_wurdemanni TACTGACCGTGCGAAGGTAGCATAATAATTAGTCTTTAATTGAGGACTGGAATGAACGGTGGACGAGAAGTTATCTGTCTCTAAGGTAGGTCTCCTTTAAGTGAAAAGGCTTAAATAACATAAAGGGACGATAAGACCCTATAAAACTTTATATGGATTACAATTAAGTTACTTTAGGGATAACAGCGTAATTTCTCTTGAGAGACCCTATCGACAGAGTTAGTTGCGACCTCGATGTTGAATTAAGATACCGTTTAGATGCAGGAGTCTAAACAGTAGGTCTGTTCGACCTTTAAAATCTT

12_Lysmata_wurdemanni TACTGACCGTGCGAAGGTAGCATAATAATTAGTCTTTAATTGAGGACTGGGATGAACGGTGGACGAGAAGTTATCTGTCTCTAAGGTAGGTCTCCTTTAAGTGAAAAGGCTTAAATAACATAAAGGGACGATAAGACCCTATAAAACTTTATATGGATTACAATTAAGTTACTTTAGGGATAACAGCGTAATTTCTCTTGAGAGACCCTATCGACAGAGTTAGTTGCGACCTCGATGTTGAATTAAGATACCGTTTAGATGCAGGAGTCTAAACAGTAGGTCTGTTCGACCTTTAAAATCTT

14_Lysmata_wurdemanni cactgaccgtgcgaaggtagcataataattagtctttaattgagggctggaatgaacggtgaacgagaagttagctgtctctaaggtaggtctcctttaagtgaaaaggcttaaataatataaagggacgataagaccctataaaactttatatggattacaattaagttactttagggataacagcgtaatttctcttgagagaccttatcgacagagctagttgcgacctcgatgttgaattaagatgccgctcaggtgcaggagcctggggagtgggtctgttcgacctttaaaatctt

12_Lysmata_wurdemanni TACTGACCGTGCGAAGGTAGCATAATAATTAGTCTTTAATTGAGGACTGGGATGAACGGTGGACGAGAAGTTATCTGTCTCTAAGGTAGGTCTCCTTTAAGTGAAAAGGCTTAAATAACATAAAGGGACGATAAGACCCTATAAAACTTTATATGGATTACAATTAAGTTACTTTAGGGATAACAGCGTAATTTCTCTTGAGAGACCCTATCGACAGAGTTAGTTGCGACCTCGATGTTGAATTAAGATATCGTTTAGATGCAGGAGTCTAAACAGTAGGTCTGTTCGACCTTTAAAATCTT

13_Lysmata_wurdemanni TACTGACCGTGCGAAGGTAGCATAATAATTAGTCTTTAATTGAGGGCTGGGATGAACGGTGGACGAGAAGTTATCTGTCTCTAAGGTAGGTTTCCTTTAAGTGAAAAGGCTTAAATAATATAAAGGGACGATAAGACCCTATAAAACTTTATATGGATTACAATTAAGTTACTTTAGGGATAACAGCGTAATTTCTCTTGAGAGACCTTATCGACAGAGTTAGTTGCGACCTCGATGTTGAATTAAGATACCGTTTAGGTGCAGGAGTCTAAACAGTAGGTCTGTTCGACCTTTAAAATCTT

15_Lysmata_gracilirostris TTCTGACCGTGCGAAGGTAGCATAGTAATTAGTCTTTAATTGAGGACTGGAATGAACGGTGGACGAGAAGTTAGCTGTCTCTAAGGCAAGGCTCTTTTAAGTGAAAAGGCTTAAATAAAATAAAGGGACGATAAGACCCTGTAAAACTTAATAGGGATTAAAATTAAGTTACTTTAGGGATAACAGCGTAATTTCTCTTGAGAGACCAAATCGACAGAGTTAGTTGCGACCTCGATGTTGAATTAAGATATCATTCAGGTGCAGGAGTCTGAATAGTAGGTCTGTTCGACCTTTAAAATCTT

16_Lysmata_nayaritensis TCTTGACCGTGCTAAGGTAGCATAGTCATTAGTCTTTAATTGAGGACTGGAATGAAAGGTGGATGAGAAGTTAGCTGTCTTTAAGACAAATCTCTTCTGAGTGAAAAGGCTTAGATAAGATAAGGGGACGACAAGACCCTATAAAACTTTATGAGGATTAAGATTAAGTTACTTTAGGGATAACAGCGTAATCTCTCTTGAGAGCCCTGATCGACAGAGTTAGTTGCGACCTCGATGTTGAATTAAGATGTTACCTAGGTGCAGGAGTCTAGGCGGTGGGTCTGTTCGACCTTTAAAATCTT

42_Lysmata_cf._anchisteus TTTTGACCGTGCAAAGGTAGCATAATGAAATGTTCTTAATTGGAGACTGGAATGAACGGTGGACAAGAAGTAAGCTGTCTCTTTAATAAAACTCTTTTAAGTGAAAAGGCTTAAATAATATAAGAAGACGATAAGACCCTATAAAACTTTATAAGGATTAAAATCAAGTTACTTTAGGGATAACAGCGTAATTTCTCTTGAGAGTTCTAATCGACAGAGCAAGTTGCGACCTCGATGTTGAATTAAGGTGTTATTCAGGTGCAGCCGCTTGAATAGTGAGTCTGTTCGACTTTTAAAACCTT

43_Lysmata_hochi TTTTGACCGTGCAAAGGTAGCATAATCAAATGTTCTTAATTGGAAACCGGAATGAACGGTGGACGAGAAGTAAGCTGTCTCTTTAATAAAGCTCTTTTAAGTGAAAAGGCTTAAATAACGTAAGAAGACGATAAGACCCTATAAAACTTTATATGGATTAAAAGTAAGTTACTTTAGGGATAACAGCGTAATTTCTCTTGAGAGTTCTGATCGACAGAGTAAGTTGCGACCTCGATGTTGAATTAAGGTGTTATCCAGGTGCAGCAGCTTGGATAGTAGGTCTGTTCGACCTTTAAAACCCT

25_Lysmata_olavoi ATTTGACCGTGCTAAGGTAGCATAATCAATAGTCTTTAATTGGGGACCGGAATGAACGGTGGACAAGAAGTTAACTGTCTCTTAAATGAATCTCTTTTAAGTGAAAAGGCTTAAATGAGTTAAAGGGACGATAAGACCCTATAAAACTTAATAAGGATTAAAATTAAGTTACTTTAGGGATAACAGCGTAATTTCTCTTGAGAGTCCTAATCGACAGAGTTAGTTGCGACCTCGATGTTGAATTAAGGTGTTATTCGGGCGCAGAAGCTCGAAAAGTAGGTCTGTTCGACCTTTAAAACCTT

**16S Additional Figure, S1B**

1_Lysmata_bahia

GGGCCGCGGTA-TACTGACCGTGCAAAGGTAGCATAATAATTAGTTCTTTAATTGAGGGCTGGAATGAAAGGT-CTTACGAGAAGGTAGCTGTCT-CTTTTATAAAGTTTGAATTTTACATTTAAGTGAGAAGGCTTAAATAAGATAAAGGGACGAGAAGACCCTATAAAACTTTACAGCTTATTTCGAAAGTGGTCTAACTAAATTA-TGTGTAACGATCCCTTT-TGTTGTAG-----------------------------------------------TTGTTTCGT-------------TGGGGCAATGGGCATAAAAA-------TAAAT--TAACTGTGTCTATTTTTATACATTTCTGAAATTTTATGTTTGTATTTATACACGGTAGTGTTGGACGTTTATTGATACATATATTGTAATA-TAAATAATT-ATAATTAGATTATTCTGATCCCTTAGTAGGGATTTAGAGATTAAGTTACTTTAGGGATAACAGCGTAATTTCTCTTGAGAGTCCAAATCGACAGAGTTAGTTGCGACCTCGATGTTGAATTAAGATGTTAGCTAGGTGCAGGAGCCTAGATAGTAGGTCTGTTCGACCTTTAAAATCTT

1_Lysmata_bahia

GGGCCGCGGTA-TACTGACCGTGCAAAGGTAGCATAATAATTAGTTCTTTAATTGAGGGCTGGAATGAAAGGT-CTTACGAGAAGGTAGCTGTCT-CTTTTATAAAGTTTGAATTTTACATTTAAGTGAGAAGGCTTAAATAAGATAAAGGGACGAGAAGACCCTATAAAACTTTACAGCTTATTTCGAAAGTGGTCTAACTAAATTA-TGTGTAACGATCCCTTT-TGTTGTAG-----------------------------------------------TTGTTTCGT-------------TGGGGCAATGGGCATAAAAA-------TAAAT--TAACTGTGTCTATTTTTATACATTTCTGAAATTTTATGTTTGTATTTATAGACGGTAGTGTTGGACGTTTATTGATACATATATTGTAATA-TAAATAATT-ATAATTAGATTATTCTGATCCCTTAGTAGGGATTTAGAGATTAAGTTACTTTAGGGATAACAGCGTAATTTCTCTTGAGAGTCCAAATCGACAGAGTTAGTTGCGACCTCGATGTTGAATTAAGATGTTAGCTAGGTGCAGGAGCCTAGATAGTAGGTCTGTTCGACCTTTAAAATCTT

2_Lysmata_bahia

GGGCCGCGGTA-TACTGACCGTGCAAAGGTAGCATAATAATTAGTTCTTTAATTGAGGGCTGGAATGAAAGGT-CTTACGAGAAGGTAGCTGTCT-CTTTTATAAAGTTTGAATTTTACATTTAAGTGAGAAGGCTTAAATAAGATAAAGGGACGAGAAGACCCTATAAAACTTTACAGCTTATTTCGAAAGTGGTCTAACTAAATTA-TGTGTAACGATCCCTTT-TGTTGTAG-----------------------------------------------CTGTTTCGT-------------TGGGGCAATGGGCATAAAAA-------TAAAT--TAACTGTGTCTATTTTTATACATTTCTGAAATTTTATGTTTGTATTTATACACGGTAGTGTTGGACGTTTATTGATACATATATTGTAATA-TAAATAATT-ATAATTAGATTATTCTGATCCCTTAGTAGGGATTTAGAGATTAAGTTACTTTAGGGATAACAGCGTAATTTCTCTTGAGAGTCCAAATCGACAGAGTTAGTTGCGACCTCGATGTTGAATTAAGATGTTAGCTAGGTGCAGGAGCCTAGATAGTAGGTCTGTTCGACCTTTAAAATCTT

49_Alope_orientalis

GGATCGCGGTA-TTCTGACCGTGCGAAGGTAGCATCATCAATAGTCTTTTAATTGGAGTCTGGAATGAACGAT-TGGACAAAAAGTAATCTGTCT-TGAGAGTAAAAATTGAAATTCACTTTTAAGTGAGAAGGCTTAAATAAGTTAGAGGGACGATAAGACCCTATAAAACTTTATGA----------------------------A-TTTTG---GGCCTATCT-AGTAATTTTAGTTTTATATTAAGATAGGTTTAGGT--------------------TCGTTTTAT-------------TGGGGCGATAAAAATATAAATA-----TCAAT--TAACTGTTTGTACACTGG-------------------------------------------------------------------AATAAA--T--------ATATTTAAA-ATTGTTGATCCTGTATTATAGATTAGTAGATAAAGTTCCTTTAGGGATAACAGCGTTATTCTTTCTGAGAGTTCTTATCGACGAAGGTAGTTGCGACCTCGATGTTGAATTAAAATTCCTTTTAGGTNNNNNNNNNNNNNNNNNNNNNNNNNNNNNNNNNNNNNNNNNN

44_Parhippolyte_mistica

GGACCGCGGTATCAATAACCGTGCGAAGGTAGCATCATCATTTGTCTTTTAATTGAAGGCTGGAATGAATGGT-CGAACGAGAAGTAAGCTGTCT-CTTTAATGTATACTGAATTTCACCTTTAAGTGAAAAGGCTTAAATGTCACGGGGGGACGATAAGACCCTATAAAGCTTTACGA------------------------------------------TTACT-TGAACTGTCTCCG--AATT--TGAGGT--ATAAGGGAGGCAGGTATCGGTG----TCGTTTCGT-------------TGGGGCGACGAGAATATAAC--------ACAG--TAACTGT-TCTTAA-----------------------------------------------------------------------AATAAA-ACGGTTG-TGATCGGTTGAG--TG--GACCCTTTATTAAGGATCATTAGATTAAGTTCCTTTAGGGATAACAGCGTTATTTCCCTTGAGAGTTCATATCGACAGGGTAAGTTGCGACCTCGATGTTGAATTAAGGTTCCATTCAGGTNNNNNNNNNNNNNNNNNNNNNNNNNNNNNNNNNNNNNNNNNN

50_Hippolyte acuta

GGGCTGCGGTA-ACTTGACCGTGCTAAGGTAGCATNATCAGTAGTCTTTTAATTGATGACTGGAATGAATGGC-GTTACGAGAAATAAGCTGTCT-TAAAAATAAAAATTGAATTTNACCTTCAAGTGAAAAGGCTTGAATACTACTAAGGGACGATAAGACCCTATAAAACTTAA------------------------------CA-TGATT---GTCTTTTCT-GANGGTTAAAGTTG-AATTAAGAATGGGTGTATT---------------------TTGTTTGGT-------------TGGGGCGACCTAGATATAAT--------TNAG--TAACTGTCTGAAAA------------------------------------------------------------------------TAAAA-ATAAT---T-ATAATTTG-GTTCA-TGAACCTTTAATAAGGATTAGAAGAAAAAGTTNCTTTAGGGATAACAGCGTAATTTTTTCAGAGAGTTCTTATCGAAGAAAGTAGTTGCGACCTCGATGTTGAATTAAAGTTNCTCTTAAGCNNNNNNNNNNNNNNNNNNNNNNNNNNNNNNNNNNNNNNNNNN

54_Heptacarpus_futilirostris

CGACCGCGGTATATCTGACCGTGCGAAGGTAGCATCATCAGTAGTCTCTTAATTGGAGGCTGGAATGAATGGT-TGCACGAGAAGAAAGCTGTTT-CTTCTTCAAGAATTGAATTTCACTTTTAAGTGAAAAGGCTTAAATAAATTGAAGGGACGATAAGACCCTATAAAACTTTA------------------------CAATTTAT-TATTG-CTCCATAAATT-TTTAGTATAA------CTTGGGTTAGT-TGTGAT--------------------ATTGTTGAGT-------------TGGGGCGACTATTATAAAATTC------TAA---TAACTGTATTCTGA-----------------------------------------------------------------------ATTAATAATT-------CTGTTTAGTTAATT--GATCCTTTTTTAAAGATTAAAAGATTAAGTTCCTTTAGGGATAACAGCGTGATTTTTTTAAAGAGTTCTTATCGATGAAATTAGTTGCGACCTCGATGTTGAATTAAAATTCCTATAAAGTNNNNNNNNNNNNNNNNNNNNNNNNNNNNNNNNNNNNNNNNNN

56_Heptacarpus_palpator

GGGCCGCGGTA-TCTTAACCGTGCGAAGGTAGCATAATCAATAGTCTTTTAATTGGAGGCTGGAATGAATGGC-TGCACGAGAAGTTAGCTGTCT-CTTTTTTAGAGATTGAATTTTACTTTTAAGTGAAAAGGCTTAAATAAACTAAAGGGACGATAAGACCCTATAAAACTTTACAATTT-----------------------ATA-TTTAC--TGCACAAATT--TTGGTATAAC-----CTAGGTAAGATGTGTT----------------------ATTGTTGGGT-------------TGGGGCGACTATTATATAAATT-----TTAA---TAACTGTAATTTAA-----------------------------------------------------------------------ATTAAT-AGAT------TTATTTAGTTTA-A-TGATCCTTTT-TAAAGATTAAAAGATTAAGTTACTTTAGGGATAACAGCGTAATTTTTTCTTAGAGTTCCTATCGACGAAATTAGTTGCGACCTCGATGTTGAATTAAAATTTCTATAAAGTGTAGCAGCTTACTTAGTTGGTCTGTTCGACCATTAAAATTTT

55_Heptacarpus_geniculatus

cgacCGCGGTA-TTTTGACCGTGCGAAGGTACAcMBATCAATAGTCTCTTAATTAGAGGCTGGAATGAATGGT-TGCACGAGAAAGAAGCTGTCT-CTTCTTTAAGAATTGACAcMBACTTTTAAGTGAAAAGGCTTAAATAATCTAAGGGGACGATAAGACCCTATAAAACTTTACAATTT-----------------------ATG-TTCAcM--BCATGAATT--TTCGTCTAAAT-----TAAGTGAAATATACT----------------------ATTGTTAGGT-------------TGGGGCGACTATTATATAGCAcMB----TAAC---AACTGTAATTTAA-----------------------------------------------------------------------AGAAATAAGTT-------TATTTAGTTAA-T-TGATCCTTTAATAAAGATTATAAGATTACAcMBCTTTAGGGATAACAGCGTAATTTTTTCTGAGAGTTCTTATCGATGAAATTAGTTGCGACCTCGATGTTGAATTAACAcMBCTTTTNNNNNNNNNNNNNNNNNNNNNNNNNNNNNNNNNNNNNNNNNNNNNN

59_Synalpheus_brevicarpus

GGACTGCGGTA-ATTTGACCGTGCAAAGGTAGCATAATCAATAGTCTTTTAATTGGAGGCTTGGATGAAAGGT-TTGACGGATGAGGAGCTGTCT-CTTTAGTTGTGTT-GAACTTAACGTTTGTGTGAAAAGGCATTAATGGTTTAGGGGGACGATAAGACCCTATAAAACTTAAC---------------------------------------------ATGT--GTGGTGTTAGCTT--TTGGAGTTGTTCTGTAATCATAAGGTGACTGGGTG-----TGTTTTGC-------------TGGGGCGGCACGAATATAA--------TTTG---TAACTGT-TTTAGG-----------------------------------------------------------------------AAAAAA-ATA-------TTGATTAATAGATTTTGGTCCTTTATTAGGGAGTTAA-GAGTAAGTTACTTTAGGGATAACAGCGTAATTTTTCTTGAGAGTTCTTATCGAAGGAAGTAGTTGTGACCTCGATGTTGAATTAAATTTTCCCTTTGGTGNNNNNNNNNNNNNNNNNNNNNNNNNNNNNNNNNNNNNNNNN

45_Barbouria_cubensis

GGGCCGCGGTATACCTGACCGTGCGAAGGTAGCATAATAATTTGTCTTTTAATTGAAGGCTCGTATGAATGGT-CGGACAAGAAGAAGGCTGTCT-TATTAATGAAAGCTGAATTTTACTTTTAAGTGAGAAGGCTTAAATAAACCAAGGGGACGATGAGACCCTATAAAACTTTA------------------------------------CA-AGATCTTAGGT-TGTCCTGTGA----------ATTAGAGGTATAAAACAGGTATAGCTTGAGT---ATTGTTTTGT-------------TGGGGCGACAGGAATAAAA---------AAGG--TAACTGTTCTTGAA-----------------------------------------------------------------------ATGAAA--TGGT---T-ATGACCAG-GTAGA-TGATCTTTTTTTAAGGATTAAAAGAGTAAGTTACTTTAGGGATAACAGCGTTATTTCCCCTGAGAGGTCTTATCGACGGGGTAAGTTGCGACCTCGATGTTGAATTAAGGTTTCACCCAGACGNNNNNNNNNNNNNNNNNNNNNNNNNNNNNNNNNNNNNNNNN

39_Exhippolysmata_oplophoroides

GGGCCGCGGTA-CTTTGACCGTGCAAAGGTAGCATAATCAGTAGTTCTTTAATTGGGAACTTGTATGAAGGGC-TGGACAAGAAGGAACCTGTCT-CTAAAATAAAATTTGAATTTCACTTTTAAGTGAAAAGGCTTAAATAAAATAAGGGGACGATAAGACCCTATAAAACTTGA--------------------------TATACA-AGAA--ATATACTAACT-CTTAGTTCTACAGT--TTAAAGGAGGG-GGTATGTGAT----------------ATTGTTTTAC-------------TGGGGCGGTAGTTATATAGGAT-----TA-GA---AACTGTAAAGTATT----------------------------------------------------------------------A-TAAA--TAAT---T-ATAATTAGAGAATA-TGATCCCTTCCTGGGGATTAAAAGATAAAGTTACTTTAGGGATAACAGCGTTATTTCTCTTGAGAGTCCAAATCGACAGAGTAAGTTGCGACCTCGATGTTGAATCAAGATGTTATTCAGGTGCAGAAGCCTGAATTGTGGGTCTGTTCGACCTTTAAAATCTT

39_Exhippolysmata_oplophoroides

GGGCCGCGGTA-CTTTGACCGTGCAAAGGTAGCATAATCAGTAGTTCTTTAATTGGGAACTTGTATGAAGGGC-TGGACAAGAAGGAACCTGTCT-CTAAAATAAAATTTGAATTTCACTTTTAAGTGAAAAGGCTTAAATAGAATAAGGGGACGATAAGACCCTATAAAACTTGA--------------------------TATACA-AGAA--ATATACTAACT-CTTAGTTCTACAGT--TTAAAGGAGGG-GGTATGTGAT----------------ATTGTTTTAC-------------TGGGGCGGTAGTTATATAGGAT-----TA-GA---AACTGTAAAGTATT----------------------------------------------------------------------A-TAAA--TAAT---T-ATAATTAGAGAATA-TGATCCCTTCCTGGGGATTAAAAGATAAAGTTACTTTAGGGATAACAGCGTTATTTCTCTTGAGAGTCCAAATCGACAGAGTAAGTTGCGACCTCGATGTTGAATCAAGATGTTATTCAGGTGCAGAAGCCTGAATTGTGGGTCTGTTCGACCTTTAAAATCTT

39_Exhippolysmata_oplophoroides

GGGCCGCGGTA-CTTTGACCGTGCAAAGGTAGCATAATCAGTAGTTCTTTAATTGGGAACTTGTATGAAGGGC-TGGACAAGAAGGAACCTGTCT-CTAAAATAAAATTTGAATTTCACTTTTAAGTGAAAAGGCTTAAATAAAATAAGGGGACGATAAGACCCTATAAAACTTGA--------------------------TATACA-AGAA--ATATACTAACT-CTTAGTTCTACAGT--CTAAAGGAGGG-GGTATGTGAT----------------ATTGTTTTAC-------------TGGGGCGGTAGTTATATAGGAT-----TA-GA---AACTGTAAAGTATT----------------------------------------------------------------------A-TAAA--TAAT---T-ATAATTAGAAAATA-TGATCCCTTCTTGGGGATTAAAAGATAAAGTTACTTTAGGGATAACAGCGTTATTTCTCTTGAGAGTCCAAATCGACAGAGTAAGTTGCGACCTCGATGTTGAATCAAGATGTTATTCAGGTGCAGAAGCCTGAATTGTGGGTCTGTTCGACCTTTAAAATCTT

40_Lysmatella_prima

GGGCCGCGGTA-TTATGACCGTGCAAAGGTAGCATAGTCACTAGTTCTTTAATTGGGTTCTGGAATGAATGGT-TGGACAAGAAGAAAACTGTCT-AGAATATAAAATTAGAATTTTACTTTTAAGTGAGAAGGCTTAAATAAAATAAGGGGACGATAAGACCCTATAAAACTTGT-----------------------------------TAC-ACAAATAAAGT-ATTACTCCTCG----AATT-ATGAAGG-TATGTTTAAACAGGGGGATACTT---AATGTTGTTCAAC----------TGGGGCGGTGCTTATATAA---------ATATGAAAACTATAAAATTGG-----------------------------------------------------------------------TTACA-ATAGT---T-ATAATTAGATTATT-AGACCCTTGTATAAGGATTTTGAGATAAAGTTACTTTAGGGATAACAGCGTTATTTCTCTTGAGAGTCCAAATCGACAGAGTAAGTTGCGACCTCGATGTTGAATCAAGATGTTATTCAGGTGGAGGAGCCTGAATTGTAGGTCTGTTCGACCTTTAAAATCTT

46_Merguia_rhizophorae

AGGCCGCGGTA-TTTTGACCGTGCGAAGGTAGCATAATCAATAGTCTTTTAATTGAAGGCTGGAATGAATGGT-TGGACGAGATGGAGGCTGTCTTCTATTATAAATTTTGAATTTTACTTTTAAGTGAAAAGGCTTAAATAATTTAGTGGGACGATAAGACCCTGTAAAGCTTTATAATTT-------------------------------------------T-ATTTGAGTTAG-----CAAAATTAGTA-CATAAGTAAACTGTTAAGTTAA----ATTGGTTATTAGGT---------TGGGGTGACTAAGATATAATA------TAAAT--TGAGAAACTGTCTT-----------------------------------------------------------------------ATAAAATATAATAATT-ATAATTAGTTTAAG-TGATCCTTAATTTAGGATTATAAGATAAAGTTACTTCAGGGATAACAGCGTAATTTCTCTTGAGAGCACATATCGACAGAGTTAGTTGCGACCTCGATGTTGAATTAAGGTAATTGTTAGGCGCAGCAGTTTATAAAATAGGTCTGTTCGACCTTTAAAACCTT

48_Merguia_oligodon

tggcCGCGGTA-TTTTGACCGTGCGAAGGTAGCACAATCAATAGTCTTTTAATTGAGGGCTGGAATGAATGGT-TGGACGAGATGGAGGCTGTCTTCTAGCGTAAATCTTGAATTCAACTTTTAAGTGAAAAGGCTTAAATGATTTAGTGGGACGATAAGACCCTGTAAAGCTTCATAATTT-------------------------------------------T-ATTTTACACAG-----CAAAATTAGTA-CATAAGTAAACTGTTGGGTGAA----ATCGATTATTAGGT---------TGGGGTGACTTAGATATAACA------TCAAT--TGAGAAACTGTCTT-----------------------------------------------------------------------ACAAAATATAATAACT-ATAATTAGCTTAGG-TGATCCTTAATTTAGGATTAAAAGATAAAGTCACTTCAGGGATAACAGCGTAATTTCTCTTGAGAGCACATATCGACAGAGTTAGTTGCGACCTCGATGTTGAATTAAGGTCATTTTTAGGNNNNNNNNNNNNNNNNNNNNNNNNNNNNNNNNNNNNNNNNNNN

57_Thor_amboinensis

TGGCCGCGGTA-TTTTGACCGTGCGAAGGTAGCATAATCAGTTGTCTTTTAATTGAAGGCTGGAATGAAAGGT-TGCACGAGAAAAAATCTGTAT-TAATTATAAGTTTTGAATTTTACTTTTAAGTGAAAAGGCTTAAATATTTTAAAGGGACGATAAGACCCTATAAAGCTTTACAATTT------------------------------------------------AATTTTATT---AAATGATTTTCAGTTTAAAGTAAATTAAA----------ATAATTTTGTTTGAT--------TGGGGTGATTATTATATATCAAATG--TAAA---TAACTGTAAATTGA-----------------------------------------------------------------------A-TAAA--TAGTG----ATAATTAGTCTA-T-TGATCCTTTTTTAAAGATTAAAAGATAAAGTTACTTTAGGGATAACAGCGTAATTTTTTCAGAGAGTTCTTATCGAAGAAAGTAGTTGCGACCTCGATGTTGAATTAAAATTTCTATTAAATGTAGCAGTTTAAGTAGTTGGTCTGTTCGACCATTAAAGTTTT

57_Thor_amboinensis

TGGCCGCGGTA-TTTTGACCGTGCGAAGGTAGCATAATCAGTTGTCTTTTAATTGAAGGCTGGAATGAAAGGT-TGCACGAGAAAAAATCTGTAT-TAATTATAAGTTTTGAATTTT-CTTTTAAGTGAAAAGGCTTAAATATTTTAAAGGGACGATAAGACCCTATAAAGCTTTACAATTT------------------------------------------------AATTTTATT---AAATGATTTTCAGTTTAAAGTAAATTAAA----------ATAATTTTGTTTGAT--------TGGGGTGATTATTATATATCAAATG--TAAA---TAACTGTAAATTGA-----------------------------------------------------------------------A-TAAA--TAGTG----ATAATTAGTCTA-T-TGATCCTTTTTTAAAGATTAAAAGATAAAGTTACTTTAGGGATAACAGCGTAATTTTTTCAGAGAGTTCTTATCGAAGAAAGTAGTTGCGACCTCGATGTTGAATTAAAATTTCTATTAAATGTAGCAGTTTAAGTAGTTGGTCTGTTCGACCATTAAAGTTTT

58_Thor_cf._manningi

TGGCCGCGGTA-TTTTGACTGTGCAAAGGTAGCATAATCAGTTGTCTTTTAATTGGAGGCTGGAATGAAAGGT-TAAACGAGAAAAAAACTGTCT-CAGCTTAAAGACTTGAATTTCCCCTTTAAGTGAAAAGGCTTAAATAATTTAAAGGGACGATAAGACCCTATAAAACTTTTATAATT-----------------------AGT-TTTAT--TCAATGATTT--TAAGTTTAAAAA--AAGTTAA-AAGG--ATT-----------------------TTATTTTAT-------------TGGGGTGATTATTATATAAACATGTG-TATAAA-TAACTGTAGTTAAA-----------------------------------------------------------------------ATTAATAATT-------ATTATTAGAAAA-A-TGATCCTTTAATAAGGATTAAAAGATAAAGTTACTTTAGGGATAACAGCGTTATTTTCTTGGAGAGTTCATATTGATAAGATAAGTTGCGACCTCGATGTTGAATTAAGGTGTCTTGTAAATGTAGCAGTTTATTAAGTTGGTCTGTTCGACCATTAAAACCTT

53_Tozeuma_carolinense

GGGCCGCGGTA-ATTTGACCGTGCGAAGGTAGCATAATCAGTAGTCTCTTAATTGGGGGCTTGTATGAAAGGT-TGGACGAGAGGAAAGCTGTCT-TTGTGATGGATCTTGAATTTTACTTTTAAGTGAAAAGTCTTAAATATACTTAAGGGACGATAAGACCCTGTAAAACTTAATAAGGC-----------------------------------------TCT--CTTGTTTAA-----AAAATATGAGTT--AAACTTTAGTTCGTGAGATTC-----TTATTTGGT-------------TGGGGCGACTGGAATATAATAG-----TATAG--TAACTGTTCATAAA-----------------------------------------------------------------------AATAAA--TAATC----ATAATTTGTAAACA-TGAGCCTTTATTAAGGATTAAAAGATAAAGTTACTTCAGGGATAACAGCGTGATTTTTTTTGAGAGTCCTTATCGACAAAAGTAGTTGCGACCTCGATGTTGAATTAAAATTTCTTTCAAATGCAGCAGTTTGATTAGTGGGTCTGTTCGACCTTTAAAATTTT

51_Hippolyte_williamsi

TGGCTGCGGTA-ATTTGACCGTGCTAAGGTAGCATAATCAATAGTTTTCTAATTTAAAACTGGAATGAATGGT-GTGACGAAAAGCAAGCTTTTT-TTAAAATATAAATTGAATTTTACTTCTGAGTGAAAAGGCTTAGATTCAGATAAGGGACGATAAGACCCTATAAAACTTAATAATAC-----------------------------------------------TTGTTTATTA---AAATTATAAAGT-TAAAAATTAACTAATTAAATC------TTATTTAGT-------------TGGGGTGACTAAGATATAAA-------TAA----TAGCTGTCTCAGTTAGATT------------------------------------------------------------------AATAAAT----------ATATTTTGTTATTA-AATCCTTTTTTAAAGATTTAAA--ACTAAGTTACTTTAGGGATAACAGCGTAATTTTTTCTGAGAGTTCTTATCGAAGAAAATAGTTGCGACCTCGATGTTGAATTAAAATTTTTGATTAACGCAGATGTTAAAGAAATAGGTCTGTTCGACCTTTAAAATTTT

52_Hippolyte_inermis

GGGCTGCGGTA-CTTTGACCGTGCTAAGGTAGCATAATCAATAGTCTCCTAACTAGGGACCGGAATGAACGGT-GTGACGAAAAGAAAGCTGTCT-CTGAGGCATAGATTGAATTTTACTCTTAAGTGAAAAGGCTTAAATATTGTTAAGGGACGATAAGACCCTGTGAAACTTAATACTAT-----------------------------------------------------CAGTTT--TTTTATTATGG-TGAAGTGAAATTTTAATTAAATTTG-ATTCTATTCAGT-----------TGGGGCGAC-----TAAAATA------TAAAGAGTAGCTGTTTGAGTT-----------------------------------------------------------------------AGAAAA-ATAAT-----GTAGTTTGTTATAA-AAAGCTTTAAGTAAAGATTAGA--ACAAAGTTACTTCAGGGATAACAGCGTAATTTTTTCTGAGAGTTCATATCGAAGAAAGTAGTTGCGACCTCGATGTTGAATTAAAGTTTTTAGTAAGCGCAGCGGCTTAAAAAATAGGTCTGTTCGACCTTAAAAATTTT

41_Lysmata_lipkei

GGGCCGCGGTA-TTTTGACCGTGCAAAGGTAGCATAATCAATAGTTTTTTAATTGAAAACTGGAATGAAAGGT-CGGACAAGAAGTAGACTGTCT-CTTTTATAAGACTTGAATTTTACTTTTAAGTGAAAAGGCTTAAATGGTTTAGGGGGACGATAAGACCCTATAAAACTTAACAGATT--------------------------------------TTATTCAGAGAGGATAA-------TTAATGTTTA-AATAGTTTTTGAGTAAA---------ACTGTTTTAC-------------TGGGGCGGTACGGATAAAA--------TAAAA--TAACTGTCTATATA------------------------------------------------------------------------TTATA-ATATT-----ATAAATAGTAAATT-TGATCCTTTACTAGGGATTAGGAGATAAAGTTACTTTAGGGATAACAGCGTAATCTCTCTTGAGAGTCCTAATCGACAGAGTAAGTTGCGACCTCGATGTTGAATTAAGGTGTTATCTAGGCGCAGAAGTCTAGACGGTAGGTCTGTTCGACCTTTAAAACCTT

26_Lysmata_acicula

GGGCCGCGGTA-TCCTGACCGTGCGAAGGTAGCATAATCAATAGTGTTTTAATTGAAGACTGGAATGAAGGGT-CGGACAAGGGGTAAGCTGTCT-CTAAAATAAATCTTGAAGTTTACCTTTAAGTGAAAAGGCTTAAATAAAGTAAAGGGACGATAAGACCCTATAAAACTTAACAAGTT---------------ATACAGCCATA-TGT---ACGAATTAACT-TTGAGTGTAA-----ACTTGATGGGTG-TACA----------------------ATTGTTTTGT-------------TGGGGCGACAGAGATAAAA--------TGAAT--TAACTGTCTTTTTT-----------------------------------------------------------------------ATTGTA-ATAGTA----ATAATTAGAGAAAT-TGATCCTTTATTAAGGATTATAAGATTAAGTTACTTTAGGGATAACAGCGTAATTTCTCTTGAGAGTCCTAATCGACAGAGTTAGTTGCGACCTCGATGTTGAATTAAGATGTTATTCAGGCGCAGCAGTNNNNNNNNNNNNNNNNNNNNNNNNNNNNNNNNNN

27_Lysmata_cf._trisetacea

GGGCCGCGGTA-TCCTGACCGTGCGAAGGTAGCATAATCAATAGTGTTTTAATTGAAGACTGGAATGAAGGGT-CGGACAAGGGGTTATCTGTCT-CTTAGATAAATCTTGAAGTTTACTTTTAAGTGAAAAGGCTTAAATGAAGTAAGGGGACGATAAGACCCTATAAAACTTAACAAATT---------------ATGCCTTTAGA-TGT---ACGAATTAACT-GGTAGTGTAA-----ACTTGATGAGGG-TATA----------------------ATTGTTTTGT-------------TGGGGCGACAGGGATAAAA--------TAAAT--TAACTGTCTTTTTT-----------------------------------------------------------------------ATTAAA-ATAGT---T-ATAATTAGAGTATT-TGATCCTTTAGTAAGGATTATAAGATTAAGTTACTTTAGGGATAACAGCGTAATTTCTCTTGAGAGTTNTAATCGACAGAGCTAGTTGCGACCTCGATGTTGAATTAAGATGTTATTCAGGCGCAGCAGTNNNNNNNNNNNNNNNNNNNNNNNNNNNNNNNNNN

28_Lysmata_galapagensis 2

GGGCCGCGGTA-TACTGACCGTGCGAAGGTAGCATAATCAATAGTGTTTTAATTGAAGACTGGAATGAATGGT-CGGACAAGGGGTTTGCTGTCT-CTAAGGTAAATCTTGAAGTTTACCTTTAAGTGAAAAGGCTTAAATGGCGTAAAGGGACGATAAGACCCTATAAAACTTGACGGATT---------------GTGCAGTTAGA-TGT-------ATGAATT-GGTAGTGTAAG-----CTTGATAGTTG-TGTAGCC----------------------GTTTTGT-------------TGGGGCGACAGGGATATAAGTA-----TAAT---TAACTGTCTTTAAT------------------------------------------------------------------------TAAAA-ATAATA----GTAATTAGTTTAAT-TGATCCTTTATTAGGGATTACGAGATTAAGTTACTTTAGGGATAACAGCGTAATTTCTCTTGAGAGTTCTAATCGACAGAGTTAGTTGCGACCTCGATGTTGAATTAAGATGTTATTCAGGCGCAGAAGTCTGAAGGGTAGGTCTGTTCGACCTTTAAAATCTT

31_Lysmata_nilita

GGGCCGCGGTA-TACTGACCGTGCGAAGGTAGCATAATCAATAGTGTTTTAATTGAAGACCGGAATGAATGGT-CGGACAAGGGGTTAGCTGTCT-CTAAGGTAAGTCTNGAAGTTTACCTTTAAGTGAAAAGGCTTAAATGACGTAAAGGGACGATAAGACCCTATAAAACTTGACAAGTT---------------ATGTAGTTAGA-TCT---ACGAATTGA-T----AGTGTAA-----ACTTGATGGTTG-CGTAG----------------------TTGTTTTGT-------------TGGGGCGACAGGGATATAAGAG-----TCGG---TAACTGTCCTTACAT-----------------------------------------------------------------------TGAAA-ATAGTGA---ATGGTTAGTTTAAT-TGATCCTCTATTAGGGATTATGAGATTAAGTTACTTTAGGGATAACAGCGTAATTTCTCTTGAGAGTTCTAATCGACAGAGTTAGTTGCGACCTCGATGTTGAATTAAGATGTTATTCAGGCGCAGGAGTCTGAAGGGTAGGTCTGTTCGACCTTTAAAATCTT

29_Lysmata_moorei

GGGCCGCGGTA-TACTGACCGTGCGAAGGTAGCATAATCAATAGTGTTTTAATTGAAGACTGGAATGAAGGGT-CGGACAAGGGGCTTGCTGTCT-CTAAGGTAAATCTTGAAGTTTACTTTTAAGTGAAAAGGCTTAAATGGCGTAGAGGGACGATAAGACCCTATAAAACTTAACGGATC---------------ATGTGGCTAGA-TGT---ATGAATTAA---GG--GTGTAAG-----CTTGATGGCCG-GGTG----------------------ATCGTTCTGT-------------TGGGGCGACAGGGATATAAG-------TATGCT-TAACTGTCTTAATG------------------------------------------------------------------------TAAAA-ATAGGA----GTAATTAGTTAAAT-TGATCCTTTATTAGGGATTATGAGATCAAGTTACTTTAGGGATAACAGCGTAATTTCTCTTGAGAGTTCTAATCGACAGAGTTAGTTGCGACCTCGATGTTGAATTAAGATGTTATTCAGGCGCAGAAGTCTGAATGGTAGGTCTGTTCGACCTTTAAAATCTT

30_Lysmata_moorei

GGGCCGCGGTA-TACTGACCGTGCGAAGGTAGCATAATCAATAGTGTTTTAATTGAAGACTGGAATGAAGGGTTCGGACAAGGGGCTTGCTGTCT-CTAAGGTAAATCTTGAAGTTTACTTTTAAGTGAAAAGGCTTAAATGGCGTAGAGGGACGATAAGACCCTATAAAACTTAACGGATC---------------ATGTGGCTAGA-TGT---ATGAATTAA---GG--GTGTAAG-----CTTGATGGCCG-GGTG----------------------ATCGTTCTGT-------------TGGGGCGACAGGGATATAAG-------TATGCT-TAACTGTCTTAATG------------------------------------------------------------------------TAAAA-ATAGGA----GTAATTAGTTAAAT-TGATCCTCTATTAGGGATTATGAGATCAAGTTACTTTAGGGATAACAGCGTAATTTCTCTTGAGAGTTCTAATCGACAGAGTTAGTTGCGACCTCGATGTTGAATTAAGATGTTATTCAGGCGCAGAAGTCTGAATGGTAGGTCTGTTCGACCTTTAAAATCTT

32_Lysmata_intermedia

GGGCCGCGGTA-TACTGACCGTGCGAAGGTAGCATAATCAATAGTGTTTTAATTGGAGACTGGAATGAACGGT-CGGACAAAGGGTTTACTGTCT-CTGAAATGAATCTTGAATTTTACTTTTAAGTGAAAAGGCTTAAATGACGTAAAGGGACGATAAGACCCTATAAAACTTGACAAGTT-------------GAATGA--TTAGA-TGT---AGAAATTAGAG----AGTGTAA-----ACTTGATGGTCA-TTCAG----------------------TTGTTTTGT-------------TGGGGCGACAAGGATAAAAGG------TAAG---TAACTGTCTTTCTG------------------------------------------------------------------------TGCAA-ATAGCA----ATGATTAGTTTATT-TGATCCTCTACTAGGGATTAAGAGATTAAGTTACTTTAGGGATAACAGCGTAATTTCTCTTGAGAGTTCTTATCGACAGAGTTAGTTGCGACCTCGATGTTGAATTAAGATGTTATTTGGGCGCAGGAGTCCAAAAAGTAGGTCTGTTCGACCTTTAAAATCTT

33_Lysmata_intermedia

GGGCCGCGGTA-TACTGACCGTGCGAAGGTAGCATAATCAATAGTGTTTTAATTGGAGACTGGAATGAACGGT-CGGACAAAGGGTTTACTGTCT-CTGAAATGAATCTTGAATTTTACTTTTAAGTGAAAAGGCTTAAATGACGTAAAGGGACGATAAGACCCTATAAAACTTGACAAGTT-------------GAATGA--TTAGA-TGT---AGAAATTAGAG----AGTGTAA-----ACTTGATGGTCA-TTCAG----------------------TTGTTTTGT-------------TGGGGCGACAAGGATAAAAGG------TAAG---TAACTGTCTTTCTG------------------------------------------------------------------------TGCAA-ATAGCA----ATGATTAGTTTATT-TGATCCTCTACTAGGGATTAAGAGATTAAGTTACTTTAGGGATAACAGCGTAATTTCTCTTGAGAGTTCTTATCGACAGAGTTAGTTGCGACCTCGATGTTGAATTAAGATGTTATTTGGGCGCAGGAGTCCGAAAAGTAGGTCTGTTCGACCTTTAAAATCTT

32_Lysmata_intermedia

GGGCCGCGGTA-TACTGACCGTGCGAAGGTAGCATAATCAATAGTGTTTTAATTGGAGACTGGAATGAACGGT-CGGACAAAGGGTTTACTGTCT-CTGAAATGAATCTTGAATTTTACTTTTAAGTGAAAAGGCTTAAATGACGTAAAGGGACGATAAGACCCTATAAAACTTGACAAGTT-------------GAATGA--TTAGA-TGT---AGAAATTAGAG----AGTGTAA-----ACTTGATGGTCA-TTCAG----------------------TTGTTTTGT-------------TGGGGCGACAAGGATAAAAGG------TAAG---TAACTGTCTTTCTG------------------------------------------------------------------------TGCAA-ATAGCA----ATGATTAGTTTATT-TGATCCTTTACTAGGGATTAAGAGATTAAGTTACTTTAGGGATAACAGCGTAATTTCTCTTGAGAGTTCTTATCGACAGAGTTAGTTGCGACCTCGATGTTGAATTAAGATGTTATTTGGGCGCAGGAGTCCAAAAAGTAGGTCTGTTCGACCTTTAAAATCTT

34_Lysmata_cf_intermedia

GGGCCGCGGTA-TACTGACCGTGCGAAGGTAGCATAATCAATAGTGTTTTAATTGGAGACTGGAATGAACGGT-CGGACAAAGGGTTTACTGTCT-CTGAAATGAATCTTGAATTTTACTTTTAAGTGAAAAGGCTTAAATGACGTAAGGGGACGATAAGACCCTATAAAACTTGACAAGCT-------------GAATGA--TTAGA-TCT---AGAAATTAGAG----AGTGTAA-----ACTTGATG--GT-TATTCAG--------------------TTGTTTTGT-------------TGGGGCGACAAGGATAAAAAGG-----TAAG---TAACTGTCTTTTTGT----------------------------------------------------------------------A-TAAA--TAGCA----ATGATTAGTTTATT-TGATCCTTTATTAGGGATTAAGAGATTAAGTTACTTTAGGGATAACAGCGTAATTTCTCTTGAGAGTTCTTATCGACAGAGTTAGTTGCGACCTCGATGTTGAATTAAGATGTTATTCGGGCGCAGGAGTCTGAAAAGTAGGTCTGTTCGACCTTTAAAATCTT

35_Lysmata_cf_intermedia

GGGCCGCGGTA-TACTGACCGTGCGAAGGTAGCATAATCAATAGTGTTTTAATTGAAGACTGGAATGAACGGT-CGGACAAGGGGTTTACTGTCT-CTAAAATGAATCTTGAAGTTTACTTTTAAGTGAGAAGGCTTAAATAATGTGGGGGGACGATAAGACCCTATAAAACTTGACAAGCT---------------ATGTGATCAGA-TGT---AGGAATTAAGT---TGGTGTAAG-----CTTGATGGTTG-TATGG----------------------TTGTTTTGT-------------TGGGGCGGCAAGGATAAAAAGA-----TAAG---TAACTGTCTTTATA------------------------------------------------------------------------TAAAA-ATAGCG----ATAATTAGTTTAGG-TGATCCCTTATTAAGGATTAAGAGATTAAGTTACTTTAGGGATAACAGCGTAATTTCTCTTGAGAGTTCTAATCGACGGAGTTAGTTGCGACCTCGATGTTGAATTAAGATGTTATTTAGGTGCAGGAGTCTAAAGAGTAGGTCTGTTCGACCTTTAAAATCTT

36_Lysmata_holthuisi

GGGCCGCGGTA-TATTGACCGTGCGAAGGTAGCATAATCAATAGTGTTTTAATTGGAGACTGGAATGAACGGT-CGGACAAGGGGTTTACTGTCT-CTGTGATGGATCTTGAAGTTTACTTTTAAGTGAAAAGGCTTAAATAATGTAAAGGGACGATAAGACCCTATAAAACTTGACAAACT---------------ATGTAATCAGG-TGT---AGGAATTAATC----AGTGTAA-----ATTTGATG---G-TTTAGTAG-------------------TTGTTTTGT-------------TGGGGCGACAGGGATAAAATAG-----TCAG---TAACTGTCTTTATG------------------------------------------------------------------------TCAAA-ATAATA----ATAATTAGTTTATT-TGATCCTTTATTAAGGATTAGGAGATTAAGTTACTTTAGGGATAACAGCGTGATTTCTCTTGAGAGTTCTAATCGACAGAGTTAGTTGCGACCTCGATGTTGAATTAAGGTGTTATTCAGGCGCAGGAGTCTGAAGAGTAGGTCTGTTCGACCTTTAAAATCTT

37_Lysmata_seticaudata

GGGCCGCGGTA-TACTGACCGTGCGAAGGTAGCATAATCAATAGTGTTTTAATTGAAGACTGGAATGAAGGGT-CGGACAAGGGGTTGGCTGTCT-CTTAAATAAAATTTGAAGTTTACTTTTAAGTGAAAAGGCTTAAATAAAGTAGAGGGACGATAAGACCCTATAAAACTTTACAAGAT---------------GTGTTGTTAGA-GGT---ATGAATTATAA----AGTGTAA-----ACTTGATAAGAA-CACA----------------------ATTGTTTTGT-------------TGGGGCGACAGGGATAAAACA-------AGAT--TAACTGTCTTTTTCT----------------------------------------------------------------------ATAATA-ATAGT---T-ATAATTAGTTAAGT-TGATCCTTTATTAAGGATTAAGAGATTAAGTTACTTTAGGGATAACAGCGTAATTTCTCTTGAGAGTTCTAATCGACAGAGTTAGTTGCGACCTCGATGTTGAATTAAGATGTTACTCAGGTGCAGCAGTCTGAGTGGTAGGTCTGTTCGACCTTTAAAATCTT

37_Lysmata_seticaudata2

GG-CCGCGGTA-TACTGACCGTGCGAAGGTAGCATAATCAATAGTGTTTTAATTGAAGACTGGAATGAAGGGT-CGGACAAGGGGTTGGCTGTCT-CTTAAATAAAATTTGAAGTTTACTTTTAAGTGAAAAGGCTTAAATAAAGTAGAGGGACGATAAGACCCTATAAAACTTTACAAGAT---------------GTGTTGTTAGA-GGT---ATGAATTATAA----AGTGTAA-----ACTTGATAAGAA-CACA----------------------ATTGTTTTGT-------------TGGGGCGACAGGGATAAAACA-------AGAT--TAACTGTCTTTTTCT----------------------------------------------------------------------ATAATA-ATAGT---T-ATAATTAGTTAAGT-TGATCCTTTATTAAGGATTAAGAGATTAAGTTACTTTAGGGATAACAGCGTAATTTCTCTTGAGAGTTCTAATCGACAGAGTTAGTTGCGACCTCGATGTTGAATTAAGATGTTACTCAGGTGCAGCAGTCTGAGTGGTAGGTCTGTTCGACCTTTAAAATCTT

38_Lysmata_ternatensis

GGGCCGCGGTA-TCCTGACCGTGCGAAGGTAGCATAATCAATAGTGTTTTAATTGAAGACTGGAATGAAGGGT-CGGACAAGGGGTTAGCTGTCT-CTAAATAAAATCTTGAAATTTACTTTTAAGTGAAAAGGCTTAAATAACGTGAGGGGACGATAAGACCCTATAAAACTTAACAAGTT---------------ATACTGTTAGA-TTT---ACGAATTAACT-GTAGGTGTAA-----ACTTGATGGTAG-TGTA----------------------ATTGTTTTGT-------------TGGGGCGACAGGGATAAAATG-------AGAT--TAACTGTCTTTTTTG-----------------------------------------------------------------------TTAAA-ATAGTA----ATAATTAGTGAACT-TGATCCTTTAATAAGGATTACAAGATTAAGTTACTTTAGGGATAACAGCGTAATTTCTCTTGAGAGTCCAAATCGACAGAGTTAGTTGCGACCTCGATGTTGAATTAAGATGTTATTCAGGCGCAGCCGTCTGAATAGTGGGTCTGTTCGACCTTTAAAATCTT

38_Lysmata_ternatensis

GGGCCGCGGTA-TCCTGACCGTGCGAAGGTAGCATAATCAATAGTGTTTTAATTGAAGACTGGAATGAAGGGT-CGGACAAGGGGTTAGCTGTCT-CTAGATAAAATCTTGAAATTTACTTTTAAGTGAAAAGGCTTAAATAACGTGAGGGGACGATAAGACCCTATAAAACTTAACAAGTT---------------ATACTGTTAGA-TTT---ACGAATTAACT-GTAGGTGTAA-----ACTTGATGGTAG-TGTA----------------------ATTGTTTTGT-------------TGGGGCGACAGGGATAAAATG-------AGAT--TAACTGTCTTTTTTG-----------------------------------------------------------------------TTAAA-ATAGTA----ATAATTAGTGAACT-TGATCCTTTAATAAGGATTACAAGATTAAGTTACTTTAGGGATAACAGCGTAATTTCTCTTGAGAGTCCAAATCGACAGAGTTAGTTGCGACCTCGATGTTGAATTAAGATGTTATTCAGGCGCAGCCGTCTGAATAGTGGGTCTGTTCGACCTTTAAAATCTT

27_Lysmata_cf._trisetacea

GGGCCGCGGTA-TCCTGACCGTGCGAAGGTAGCATAATCAATAGTGTTTTAATTGAAGACTGGAATGAAGGGT-CGGACAAGGGGTTATCTGTCT-CTTAGATAAATCTTGAAGTTTACTTTTAAGTGAAAAGGCTTAAATGAAGTAAGGGGACGATAAGACCCTATAAAACTTAACAAATT---------------ATGCCTTTAGA-TGT---ACGAATTAACT-GCTAGTGTAA-----ACTTGATGAGGG-TATA----------------------ATTGTTTTGT-------------TGGGGCGACAGGGATAAAA--------TAAAT--TAACTGTCTTTTTT-----------------------------------------------------------------------ATTAAA-ATAGTC----ATAATTAGAGTATT-TGATCCTTTAGTAAGGATTATAAGATTAAGTTACTTTAGGGATAACAGCGTAATTTCTCTTGAGAGTTCTAATCGACAGAGCTAGTTGCGACCTCGATGTTGAATTAAGATGTTATTCAGGCGCAGCAGTCTGAATGGTAGGTCTGTTCGACCTTTAAAATCTT

27_Lysmata_cf._trisetacea

GGGCCGCGGTA-TCCTGACCGTGCGAAGGTAGCATAATCAATAGTGTTTTAATTGAAGACTGGAATGAAGGGT-CGGACAAGGGGTTATCTGTCT-CTTAGATAAATCTTGAAGTTTACTTTTAAGTGAAAAGGCTTAAATGAAGTAAGGGGACGATAAGACCCTATAAAACTTAACAAATT---------------ATGCTTTTAGA-TGT---ACGAATTAACT-GGTAGTGTAA-----ACTTGATGAGGG-TATA----------------------ATTGTTTTGT-------------TGGGGCGACAGGGATAAAA--------TAAAT--TAACTGTCTTTTTT-----------------------------------------------------------------------ATTAAA-ATAGTC----ATAATTAGAGTATT-TGATCCTTTAGTAAGGATTATAAGATTAAGTTACTTTAGGGATAACAGCGTAATTTCTCTTGAGAGTTCTAATCGACAGAGCTAGTTGCGACCTCGATGTTGAATTAAGATGTTATTCAGGCGCAGCAGTCTGAATGGTAGGTCTGTTCGACCTTTAAAATCTT

27_Lysmata_cf._trisetacea

GGGCCGCGGTA-TCCTGACCGTGCGAAGGTAGCATAATCAATAGTGTTTTAATTGAAGACTGGAATGAAGGGT-CGGACAAGGGGTTATCTGTCT-CTTAGATAAATCTTGAAGTTTACTTTTAAGTGAAAAGGCTTAAATGAAGTAAGGGGACGATAAGACCCTATAAAACTTAACAAATT---------------ATGCCTTTAGA-TGT---ACGAATTAACT-GGTAGTGTAA-----ACTTGATGGGGG-TATA----------------------ATTGTTTTGT-------------TGGGGCGACAGGGATAAAA--------TAAAT--TAACTGTCTTTTCT-----------------------------------------------------------------------ATTAAA-ATAGT---T-ATAATTAGAGTATT-TGATCCTTTAGTAAGGATTATAAGATTAAGTTACTTTAGGGATAACAGCGTAATTTCTCTTGAGAGTTCTAATCGACAGAGCTAGTTGCGACCTCGATGTTGAATTAAGATGTTATTCAGGCGCAGCAGTCTGAATGGTAGGTCTGTTCGACCTTTAAAATCTT

17_Lysmata_amboinensis

GGGCCGCGGTA-TACTGACCGTGCGAAGGTAGCATAATCAATAGTTCTTTAATTGAGGACTGGAATGAAGGGT-CGGACGAGAAGTTAGCTGTCT-CCAAGACAAGTCTTGAAGTTTACTTTTAAGTGAAAAGGCTTAAATGAGATAAAGGGACGATAAGACCCTATAAAACTTTACAGTTT---------------ATGTCTAGA-A-TTT-----GTTTAAATT--TGCGTGTAA-----ATCTGTTTTGGT--AAAGC----------------------TGTTTTAT-------------TGGGGCGATAAGAATATAA--------TCGAT--TAACTGTTTTTAGTT----------------------------------------------------------------------ATGAAA-ATAGG---T-ATAATTAGTTAA-TTTGATCCTTTAATAAGGATTAGAAGATTAAGTTACTTTAGGGATAACAGCGTAATTTCTCTTGAGAGTTCTAATCGACAGAGTTAGTTGCGACCTCGATGTTGAATTAAGGTGTTAGCTAGGCGCAGAAGCTTAGATAGTAGGTCTGTTCGACCTTTAAAACCTT

17_Lysmata_amboinensis

GGGCCGCGGTA-TACTGACCGTGCGAAGGTAGCATAATCAATAGTTCTTTAATTGAGGACTGGAATGAAGGGT-CGGACGAGAAGTTAGCTGTCT-CCAAGACAAGTCTTGAAGTTTACTTTTAAGTGAAAAGGCTTAAATGAGATAAAGGGACGATAAGACCCTATAAAACTTTACAGTTT---------------ATGTCTTGA-A-TTT-----GTTTAAATT--TGCGTGTAA-----ATCTGTTTTGGT--AAAGC----------------------TGTTTTAT-------------TGGGGCGATAAGAATATAA--------TCGAT--TAACTGTTTTTAGTT----------------------------------------------------------------------ATGAAA-ATAGG---T-ATAATTAGTTAA-TTTGATCCTTTAATAAGGATTAGAAGATTAAGTTACTTTAGGGATAACAGCGTAATTTCTCTTGAGAGTTCTAATCGACAGAGTTAGTTGCGACCTCGATGTTGAATTAAGGTGTTAGCTAGGCGCAGAAGCTTAGATAGTAGGTCTGTTCGACCTTTAAAACCTT

18_Lysmata_amboinensis

GGGCCGCGGTA-TACTGACCGTGCGAAGGTAGCATAATCAATAGTTCTTTAATTGAGGACTGGAATGAAGGGT-CGGACGAGAAGTTAGCTGTCT-CCAAGACAAGTCTTGAAGTTTACTTTTAAGTGAAAAGGCTTAAATAAAATAAAGGGACGATAAGACCCTATAAAACTTTACAGTTT---------------ATGTCTAGA-A-TTT-----GTTTAAATT--TGNGTGTAA-----ATCTGTTTTGGT--AAAGC----------------------TGTTTTAT-------------TGGGGCGATAAGAATATAA--------TCAAT--TAACTGTTTTTAGTTG----------------------------------------------------------------------TGAAA-ATAAGGT-TAATAATTAGTTAA-TTTGATCCTTTAATAAGGATTAANAGATTAAGTTACTTTAGGGATAACAGCGTAATTTCTCTTGAGAGTTCTAATCGACAGAGTTAGTTGCGACCTCGATGTTGAATTAAGGTGTTAGCTAGGCGCAGAGACTTAGATAGTAGGTCTGTTCGACCTTTAAAACCTT

19_Lysmata_grabhami

GGGCCGCGGTA-TACTGACCGTGCGAAGGTAGCATAATCAATAGTTCTTTAATTGAGGACTGGAATGAAGGGT-CGGACGAGAAGTAAGCTGTCT-CCAAGGCAAATCTTGAAGTTTACTTTTAAGTGAGAAGGCTTAAATAAGATAAAGGGACGATAAGACCCTATAAAACTTTACAGTAT----------------AGGCTTGGAA-TTT-----GTTTAAATT-TGT-GTGTAAAT----CTGTTTTAGCG-TAGC------------------------TGTTTTAT-------------TGGGGCGATAGGGATATAA--------TCAAT--TAACTGTTTTTAGTTGTAAA-----------------------------------------------------------------AATAAA-A---------ATAATTAGTTAA-CTTGATCCTTTAATAAGGATTAGAAGATTAAGTTACTTTAGGGATAACAGCGTAATTTCTCTTGAGAGTTCTAATCGACAGAGTTAGTTGCGACCTCGATGTTGAATTAAGGTGTTAGCTAGGCGCAGAAGCTTAGATAGTAGGTCTGTTCGACCTTTAAAACCTT

19_Lysmata_grabhami

GGGCCGCGGTA-TACTGACCGTGCGAAGGTAGCATAATCAATAGTTCTTTAATTGAGGACTGGAATGAAGGGT-CGGACGAGAAGTAAGCTGTCT-CCAAGGCAAATCTTGAAGTTTACTTTTAAGTGAGAAGGCTTAAATAAGATAAAGGGACGATAAGACCCTATAAAACTTTACAGTAT----------------AGGCTTGGAA-TTT-----GTTTAAATT-TGT-GTGTAAAT----CTGTTTTAGCG-TAGC------------------------TGTTTTAT-------------TGGGGCGATAGGGATATAA--------TCAAT--TAACTGTTTTTAGTTGTGAA-----------------------------------------------------------------AATAAA-A---------ATAATTAGTTAA-CTTGATCCTTTAATAAGGATTAGAAGATTAAGTTACTTTAGGGATAACAGCGTAATTTCTCTTGAGAGTTCTAATCGACAGAGTTAGTTGCGACCTCGATGTTGAATTAAGGTGTTAGCTAGGCGCAGAAGCTTAGATAGTAGGTCTGTTCGACCTTTAAAACCTT

19_Lysmata_grabhami

GGGCCGCGGTA-TACTGACCGTGCGAAGGTAGCATAATCAATAGTTCTTTAATTGAGGACTGGAATGAAGGGT-CGGACGAGAAGTAAGCTGTCT-CCAAGGCAAATCTTGAAGTTTACTTTTAAGTGAGAAGGCTTAAATAAGATAAAGGGACGATAAGACCCTATAAAACTTTACAGTAT---------------AGGNNTGGA-A-TTT-----GTTTAAATT-TGT-GTGTAAAT----CTGTNTTAGCG-TAGC------------------------TGTTTTAT-------------TGGGGCGATANGNATATAA--------TCAAT--TAACTGTTTTTAGTTGTAAA-----------------------------------------------------------------AATAAA-A---------ATAATTAGTTAA-CTTGATCCTTTAATAAGGATTAGAAGATTAAGTTACTTTAGGGATAACAGCGTAATTTCTCTTGAGAGTTCTAATCGACAGAGTTAGTTGCGACCTCGATGTTGAATTAAGGTGTTAGCTAGGCGCAGAAGCTTAGATAGTAGGTCTGTTCGACCTTTAAAACCTT

20_Lysmata_grabhami

GGGCCGCGGTA-TACTGACCGTGCGAAGGTAGCATAATCAATAGTTCTTTAATTGAGGACTGGAATGAAGGGT-CGGACGAGAAGTAAGCTGTCT-CCAAGGCAAATCTTGAAGTTTACTTTTAAGTGAGAAGGCTTAAATAAGATAAAGGGACGATAAGACCCTATAAAACTTTACAGTAT--------------------------------------------AGGTT-TGGAATTTG-TTTAAATTAGTG-TGTAAATCTGTTTTAGCGTAGC------TGTTTTAT-------------TGGGGCGATAGGGATATAA--------TCAAT--TAACTGTTTTTAGTTGTAAA-----------------------------------------------------------------AATAAA-A---------ATAATTAGTTAA-CTTGATCCTTTAATAAGGATTAGAAGATTAAGTTACTTTAGGGATAACAGCGTAATTTCTCTTGAGAGTTCTAATCGACAGAGTTAGTTGCGACCTCGATGTTGAATTAAGGTGTTAGCTAGGCGCAGAAGCTTAGATAGTAGGTCTGTTCGACCTTTAAAACCTT

21_Lysmata_grabhami

GGGCCGCGGTA-TACTGACCGTGCGAAGGTAGCATAATCAATAGTTCTTTAATTGAGGACTGGAATGAAGGGT-CGGACGAGAAGTAAGCTGTCT-CCAAGACAAATCTTGAAGTTTACTTTTAAGTGAAAAGGCTTAAATAAGATAAAGGGACGATAAGACCCTATAAAACTTTACAGTATT--------------AGGTCTANA-A-TTT-----GTTTAAATT-TGT-GTGTAAAT----CTGTTTTAGCG-TANC------------------------TGTTTTAT-------------TGGGGCGATANGNATATAA--------TCAAT--TAACTGTTTTTAGTTGTAAA-----------------------------------------------------------------AATAAA-A---------ATAATTAGTTAATCTTGATCCTTTAATAAGGATTANAAGATTAAGTTACTTTAGGGATAACAGCGTAATTTCTCTTGAGAGTTCTAATCGACAGAGTTAGTTGCGACCTCGATGTTGAATTAAGGTGTTAGCTAGGCGCAGAAGCTTAGATAGTAGGTCTGTTCGACCTTTAAAACCTT

22_Lysmata_debelius GGGCCGCGGTA-TATTGACCGTGCGAAGGTAGCATAATCAATAGTTCTTTAATTGAGGACTGGAATGAAGGGT-CGGACGAGAAGTAAGCTGTCT-CTAAAATAAATCTTGAAGTTTACTTTTAAGTGAAAAGGCTTAAATAAGATAAAGGGACGATAAGACCCTATAAAACTTTACAGATT---------------------------------ACATCTAAAAT---TTGTTTGA-----AATT-ATGTGTA-AATTCGTTTTAGTGTAGC----------TGTTTTGT-------------TGGGGCGACAGAGATATAA--------TTAAT--TAACTGTTTTTGGTG----------------------------------------------------------------------ATTAAA-ATAATA----CTAATTAGTTAA-TTTGATCCATTAATAAGGATTAAAAGATTAAGTTACTTTAGGGATAACAGCGTAATTTCTCTTGAGAGTTCTAATCGACAGAGTTAGTTGCGACCTCGATGTTGAATTAAGGTGTTAGCTAGGCGCAGAAGCTTAGATAGTAGGTCTGTTCGACCTTTAAAACCTT

23_Lysmata_debelius

GGGCCGCGGTA-TATTGACCGTGCGAAGGTAGCATAATCAATAGT-CTTTAATTGAGGACTGGAATGAAGGGT-CGGACGAGAAGTAAGCTGTCT-CTAAAATAAATCTTGAAGTTTACTTTTAAGTGAAAAGGCTTAAATAAGATAAAGGGACGATAAGACCCTATAAAACTTTACAGATT---------------------------------ACATCTAAAAT---TTGTTTGA-----AATT-ATGTGTA-AATTCGTTTTAGTGTAGC----------TGTTTTGT-------------TGGGGCGACAGAGATATAA--------TTAAT--TAACTGTTTTTGGTG----------------------------------------------------------------------ATTAAA-ATAATA----CTAATTAGTTAA-TTTGATCCATTAATAAGGATTAAAAGATTAAGTTACTTTAGGGATAACAGCGTAATTTCTCTTGAGAGTTCTAATCGACAGAGTTAGTTGCGACCTCGATGTTGAATTAAGGTGTTAGCTAGGCGCAGAAGCTTAGATAGTAGGTCTGTTCGACCTTTAAAACCTT

22_Lysmata_debelius

GGGCCGCGGTA-TATTGACCGTGCGAAGGTAGCATAATCAATAGTCCTTTAATTGGGGACTGGAATGAAGGGT-CGGACGAGAAGTAAGCTGTCT-CTAAAATAAATCTTGAAGTTTACTTTTAAGTGAAAAGGCTTAAATAAGATAAAGGGACGATAAGACCCTATAAAACTTTACAGATT-----------------------------------------------ACATTTAAA----ATTTGTTAGAAATTATGTGTAAATTCGTTTTAATGTAG--CTGTTTTGT-------------TGGGGCGACAGAGATATAA--------TTAAT--TAACTGTTTTTGGTG----------------------------------------------------------------------ATTAAA-ATAATA----CTAATTAGTTAA-TTTGATCCATTATTAAGGATTATAAGATTAAGTTACTTTAGGGATAACAGCGTAATTTCTCTTGAGAGTTCTAATCGACAGAGTTAGTTGCGACCTCGATGTTGAATTAAGGTGTTAGCTAGGCGCAGAAGCTTAGATAGTAGGTCTGTTCGACCTTTAAAACCTT

24_Lysmata_californica

GGGCCGCGGTA-TACTGACCGTGCGAAGGTAGCATAATCATTAGTTCTTTAATTGAGGACTGGAATGAAAGGT-TGGACGAGAAGTTAACTGTCT-CTAAAATAAATCTTGAAGTTTACTTTTAAGTGAAAAGGCTTAAATAAAATAAAGGGACGATAAGACCCTATAAAACTTTACAGGTC------------------------------------------------ATTTGAAAATT-ACTCAATTAAGG-TGTAAATGTGTTTTTAATGTT-----ACTGTTTTGT-------------TGGGGCGATAGAGATATAA--------TTAAT--TAACTGTTTTTTGT-----------------------------------------------------------------------ATTAAA-ATAATA----CTAATTAGAAAA-AGTGATCCTTTAATAAGGATTAAAAGATTAAGTTACTTTAGGGATAACAGCGTAATTTCTCTTGAGAGTTCCAATCGACAGAGTTAGTTGCGACCTCGATGTTGAATTAAGATGTTAGTTAGGCGCAGAAGCTTAAGTAGTAGGTCTGTTCGACCTTTAAAATCTT

24_Lysmata_californica

GGGCCGCGGTA-TACTGACCGTGCGAAGGTATCATAATCATTAGTTCTTTAATTGAGGACTGGAATGAAAGGT-TGGACGAGAAGTTAACTGTCT-CTAAAATAAATCTTGAAGTTTACTTTTAAGTGAAAAGGCTTAAATGAAATAAAGGGACGATAAGACCCTATAAAACTTTACAGGCC----------------------------------------------GTTTTGAAAATT--ATTCAATTAAGG-TGTAAATGTGTTTTTAATGTG-----ACTGTTTTGT-------------TGGGGCGATAGAGATATAA--------TTGAT--TAACTGTTTTTTGAG-----------------------------------------------------------------------TTGAA-ATAATA----TTAATTAGGGAA-TATGATCCTTTAATAAGGATTAAAAGATTAAGTTACTTTAGGGATAACAGCGTAATTTCTCTTGAGAGTTCTGATCGACAGAGTTAGTTGCGACCTCGATGTTGAATTAAGATGTTAGTTAGGCGCAGAAGCTTAAGTAGTAGGTCTGTTCGACCTTTAAAATCTT

3_Lysmata_ankeri

GGGCCGCGGTA-CACTGACCGTGCAAAGGTAGCATAGTAATTAGTTCTTTAATTGAGGACTGGGATGAACGGT-TGGACGAGAAGTTGGCTGTCT-CTAGTATAAGTTTTGAATTTTACTTTTAAGTGAAAAGGCTTAAATAAAATAAAGGGACGATAAGACCCTATAAAACTTTACTTTCT------------------TCTTTAAA-AGTGT-AAGAGTAAA------AGTGTAAT----ATTGCTTTTGGGGCAGA------------------------TGTTTTAT-------------TGGGGCGATAGAAATATAA---------AATTGTTAACTGTTTTCTTTATTATA-----------------------------------------------------------------ATAATA-ATAATAA-TAATAATTAGACTAAAATGATCCTTTATTATGGAGTACGAGATTAAGTTACTTTAGGGATAACAGCGTAATTTCTCTTGAGAGTTCTAATCGACAGAGTTAGTTGCGACCTCGATGTTGAATTAAGATATCACTTAGGTGGAGGAGCTTAAGTAGTAGGTCTGTTCGACCTTTAAAATCTT

3_Lysmata_ankeri GGGCCGCGGTA-CACTGACCGTGCAAAGGTAGCATAGTAATTAGTTCTTTAATTGAGGACTGGGATGAACGGT-TGGACGAGAAGTTGGCTGTCT-CTAGTATAAGTTTTGAATTTTACTTTTAAGTGAAAAGGCTTAAATAAAATAAAGGGACGATAAGACCCTATAAAACTTTACTTTCT------------------TCTTTAAA-AGTGT-AAGAGTAAA------AGTGTAAT----ATTGCTTTTGGGGCAGA------------------------TGTTTTAT-------------TGGGGCGATAGAAATATAA---------AATTGTTAACTGTTTTCTTTATT--------------------------------------------------------------------ATAATA-ATAATAA-TAATAATTAGACTAAAATGATCCTTTATTATGGAGTACGAGATTAAGTTACTTTAGGGATAACAGCGTAATTTCTCTTGAGAGTTCTAATCGACAGAGTTAGTTGCGACCTCGATGTTGAATTAAGATATCACTTAGGTGGAGGAGCTTAAGTAGTAGGTCTGTTCGACCTTTAAAATCTT

3_Lysmata_ankeri

GGGCCGCGGTA-CACTGACCGTGCAAAGGTAGCATAGTAATTAGTTCTTTAATTGAGGACTGGGATGAACGGT-TGGACGAGAAGTTGGCTGTCT-CTAGTATAAGTTTTGAATTTTACTTTTAAGTGAAAAGGCTTAAATAAAATAAAGGGACGATAAGACCCTATAAAACTTTACTTTCT------------------TCTTTAAA-AGTGT-AAGAGTAAA------AGTGTAAT----ATTGCTTTTGGGGCAGA------------------------TGTTTTAT-------------TGGGGCGATAGAAATATAA---------AATTGTTAACTGTTTTCTTTATTAGA-----------------------------------------------------------------ATAATA-ATAATAA-TAATAATTAGACTAAAATGATCCTTTATTATGGAGTACGAGATTAAGTTACTTTAGGGATAACAGCGTAATTTCTCTTGAGAGTTCTAATCGACAGAGTTAGTTGCGACCTCGATGTTGAATTAAGATATCACTTAGGTGGAGGAGCTTAAGTAGTAGGTCTGTTCGACCTTTAAAATCTT

3_Lysmata_ankeri

GGGCCGCGGTA-CACTGACCGTGCAAAGGTAGCATAGTAATTAGTTCTTTAATTGAGGACTGGGATGAACGGT-TGGACGAGAAGTTGGCTGTCT-CTAGTATAAGTTTTGAATTTTACTTTTAAGTGAAAAGGCTTAAATAAAATAAAGGGACGATAAGACCCTATAAAACTTTACTTTCT------------------TCTTTAAA-AGTGT-AAGAGTAAA------AGTGTAAT----ATTGCTTTTGGGGCAGA------------------------TGTTTTAT-------------TGGGGCGATAGAAATATAA---------AATTGTTAACTGTTTTCTTTACTATA-----------------------------------------------------------------ATAATA-ATAATAA-TAATAATTAGACTAAAATGATCCTTTATTATGGAGTACGAGATTAAGTTACTTTAGGGATAACAGCGTAATTTCTCTTGAGAGTTCTAATCGACAGAGTTAGTTGCGACCTCGATGTTGAATTAAGATATCACTTAGGTGGAGGAGCTTAAGTAGTAGGTCTGTTCGACCTTTAAAATCTT

4_Lysmata_ankeri

GGGCCGCGGTA-CCCTGACCGTGCAAAGGTAGCATAGTAATTAGTTCTTTAATTGAGGACTGGGATGAACGGT-TGGACGAGAAGTTGGCTGTCT-CTAATATAAATTTTGAATTTTACTTTTAAGTGAAAAGGCTTAAATAAAATAAAGGGACGATAAGACCCTGTAAAACTTTACTTTCT-----------------GCTTTTAAAGTGT---AAGAGTAAA------AGTGTAATATT-GCTT--TTAGGG-TAGA------------------------TGTTTTAT-------------TGGGGCGGTAGAAATATAA---------AATTGTTAACTGTTTTCTTTTATTATAGCA-------------------------------------------------------------ATAATA-ATAATAA-TAATAATTAGACTAAA-TGATCCTTTACTATGGACTACGAGATTAAGTTACTTTAGGGATAACAGCGTAATTTCTCTTGAGAGTCCTAATCGACAGAGTTAGTTGCGACCTCGATGTTGAATTAAGATATCACCTAGGCGGAGGAGCTTAGGTAGTAGGTCTGTTCGACCTTTAAAATCTT

5_Lysmata_pederseni

GGGCCGCGGTA-TACTGACCGTGCAAAGGTAGCATAATAATTAGTTCTTTAATTGAGGACTGGGATGAACGGT-TGGACGAGAAGTTAGCTGTCT-CTAATATAAACTTTGAACTTTACCTATAAGTGAAAAGGCTTATATATAATAAAGGGACGATAAGACCCTATAAAACTTTACTTTCT------------------TTTCTAAA-AGTTT--GAGAGTAAA-----AGTGTAATATTTGCTT--TTAGGG-TAGA------------------------TGTTTTAT-------------TGGGGCGATAGAAATATAAACA------ATAT--TAACTGTTTTTTTGATG---------------------------------------------------------------------TAATA-ATAGTA----ATAATTAGAATAAG-TGATCCTTTATTAAGGACTACAAGATTAAGTTACTTTAGGGATAACAGCGTAATTTCTCTTGAGAGACCTAATCGACAGAGTTAGTTGCGACCTCGATGTTGAATTAAGATGTTACTTAGGCGGAGGAGCTTAAGTAGTAGGTCTGTTCGACCTTTAAAATCTT

8_Lysmata_pederseni

GGGCCGCGGTA-TACTGACCGTGCAAAGGTAGCATAATAATTAGTTCTTTAATTGAGGACTGGGATGAACGGT-TGGACGAGAAGTTAGCTGTCT-CTAATATAAACTTTGAACTTTACCTATAAGTGAAAAGGCTTATATATAATAAAGGGACGATAAGACCCTATAAAACTTTACTTTCT------------------TTTCTAAA-AGTTT--GAGAGTAAA-----AGTGTAATATTTGCTT--TTAGGG-TAGA------------------------TGTTTTAT-------------TGGGGCGATAGAAATATAAACA------ATAT--TAACTGTTTTTTGATG----------------------------------------------------------------------TAATA-ATAGTA----ATAATTAGAATAAG-TGATCCTTTACTAAGGACTACAAGATTAAGTTACTTTAGGGATAACAGCGTAATTTCTCTTGAGAGACCTAATCGACAGAGTTAGTTGCGACCTCGATGTTGAATTAAGATGTCACTTAGGCGGAGGAGCTTAAGTAGTAGGTCTGTTCGACCTTTAAAATCTT

6_Lysmata_pederseni

GGGCCGCGGTA-TACTGACCGTGCAAAGGTAGCATAATCATTAGTTCTTTAATTGAGGACTGGGATGAACGGT-TGGACGAGAAGTTAGCTGTCT-CTAATATAAACTTTGAACTTTACCTTTAAGTGAAAAGGCTTATATATAATAAAGGGACGATAAGACCCTATAAAACTTTACTTTCT------------------TCTNTAAA-AGTTT--GAGAGTAAA-----AGTGTAATATTTGCTT--TTAGGG-TAGA------------------------TGTTTTAT-------------TGGGGCGATAGAAATATAAACA------ATAT--TAACTGTTTTTTTGATG---------------------------------------------------------------------TAATA-ATAGTA----ATAATTAGAATAAG-TGATCCTCTACTAAGGACTACAAGATTAAGTTACTTTAGGGATAACAGCGTAATTTCTCTTGAGAGACCTAATCGACAGAGTTAGTTGCGACCTCGATGTTGAATTAAGATGTTACTTAGGCGGAGGAGCTTAAGTAGTAGGTCTGTTCGACCTTTAAAATCTT

7_Lysmata_pederseni

GGGCCGCGGTA-TACTGACCGTGCAAAGGTAGCATAATCATTAGTTCTTTAATTGAGGACTGGGATGAACGGT-TGGACGAGAAGTTAGCTGTCT-CTAATATAAACTTTGAACTTTACCTTTAAGTGAAAAGGCTTATATATAATAAAGGGACGATAAGACCCTATAAAACTTTACTTTCT------------------TCTNTAAA-AGTTT--GAGAGTAAA-----AGTGTAATATTTGCTT--TTAGGG-TAGA------------------------TGTTTTAT-------------TGGGGCGATAGAAATATAAACA------ATAT--TAACTGTTTTTTTGATG---------------------------------------------------------------------TAATA-ATAGTA----ATAATTAGAATAAG-TGATCCTCTATTAAGGACTACAAGATTAAGTTACTTTAGGGATAACAGCGTAATTTCTCTTGAGAGACCTAATCGACAGAGTTAGTTGCGACCTCGATGTTGAATTAAGATGTTACTTAGGCGGAGGAGCTTAAGTAGTAGGTCTGTTCGACCTTTAAAATCTT

9_Lysmata_boggessi

GGGCCGCGGTA-CTCTGACCGTGCAAAGGTAGCATAGTAATTAGTTCTTTAATTGAGGACTGGAATGAACGGT-TGGACGAGAAGTTAGCTGTCT-CTAATGTAAATCTTGAAGTTTACTTTTAAGTGAAAAGGCTTAAATAATATAAAGGGACGATAAGACCCTATAAAACTTTACTTCTT-------------------GTTTAAAAAGTAG-TTGAATTAA---GGTA---TAA-----ATTTGCTTTTGG-AATA----------------------AGTGTTTTGT-------------TGGGGCGATAGAAATATAA----------ATTGTTAACTGT-TTTTAA-----------------------------------------------------------------------ATCAAAAATAATG----ATAATTAGTTTAAA-TGATCCTTTAGTATGGATTATAAGATTAAGTTACTTTAGGGATAACAGCGTAATTTCTCTTGAGAGACCTAATCGACAGAGTTAGTTGCGACCTCGATGTTGAATTAAGATATCATTTAGGTGTAGAAGTCTAAATAGTAGGTCTGTTCGACCTTTAAAATCTT

10_Lysmata_rafa

GGGCCGCGGTA-CACTGACCGTGCGAAGGTAGCATAGTAATTAGTTCTTTAATTGAGGACTGGAATGAACGGT-TGGACGAGAAGTTAGCTGTCT-CTAATGTAAATTTTGAAGTTTACCTTTAAGTGAAAAGGCTTAAATAATATAAAGGGACGATAAGACCCTATAAAACTTTACTTCTT-------------------------ATTTTAAGAGTTATTAAATTAGAGGTGTAA-----ATTTGCTCTTTA-AATA----------------------AGTGTTTTGT-------------TGGGGCGATAGGAATATAAA--------ATAT--TAACTGTTTTTGAA-----------------------------------------------------------------------AATAAA-ATAATA----GTAATTAGATTAAA-TGATCCTTTAATATGGATNATAAGATTAAGCTACTTTAGGGATAACAGCGTAATTTCTCTTGAGAGACCNAATCGACAGAGTTAGTTGCGACCTCGATGTTGAATTAAGATATCATTTAGGTGCAGAAGTCTAAATAGTAGGTCTTTTCGACCTTNAAAATCCT

11_Lysmata_rafa

GGGCCGCGGTA-TACTGACCGTGCGAAGGTAGCATAGTAATTAGTTCTTTAATTGAGGACTGGAATGAACGGT-TGGACGAGAAGTTAACTGTCT-CGGGTATAAATTTTGAAGTTTACTTTTAAGTGAAAAGGCTTAAATAATATAAAGGGACGATAAGACCCTATAAAACTTTACTCCTT-----------------GCTTTTATAGTTT--------TTAAATTGTAAGTGTAA-----ATTTGCTCTTCG-AGCA----------------------AGTGTTTTGT-------------TGGGGCGATAAAAATATAAA--------ATAT--TAACTGT-TTTTGA-----------------------------------------------------------------------AATAAA-ATAATA----ATAATTAGATTAGA-TGATCCTTTAATATGGATTACAAGACCAAGTTACTTTAGGGATAACAGCGTAATTTCTCTTGAGAGACCAAATCGACAGAGTTAGTTGCGACCTCGATGTTGAATTAAGATATCATTTAGGCGTAGAAGTCTAAATAGTAGGTCTGTTCGACCTTTAAAATCTT

12_Lysmata_wurdemanni

GGGCCGCGGTA-TACTGACCGTGCGAAGGTAGCATAATAATTAGTTCTTTAATTGAGGACTGGAATGAACGGT-TGGACGAGAAGTTATCTGTCT-CTAAGGTAGGTCTTGAATTTTACCTTTAAGTGAAAAGGCTTAAATAACATAAAGGGACGATAAGACCCTATAAAACTTTACTTTCT-----------------ATTTTTATAATTTTT---AAATTAACG------TGTAA-----ATTTGTTATTGG-GATAG---------------------A-TGTTTTGT-------------TGGGGCGGCAGGAATATAAA--------ATAT--TAACTGTTCTTGGATT---------------------------------------------------------------------ATGAAA--TAATA----ATAATTAGATTAAT-TGATCCTATACTATGGATTACAAGATTAAGTTACTTTAGGGATAACAGCGTAATTTCTCTTGAGAGACCCTATCGACAGAGTTAGTTGCGACCTCGATGTTGAATTAAGATACCGTTTAGATGCAGGAGTCTAAACAGTAGGTCTGTTCGACCTTTAAAATCTT

12_Lysmata_wurdemanni

GGGCCGCGGTA-TACTGACCGTGCGAAGGTAGCATAATAATTAGTTCTTTAATTGAGGACTGGGATGAACGGT-TGGACGAGAAGTTATCTGTCT-CTAAGGTAGGTCTTGAATTTTACCTTTAAGTGAAAAGGCTTAAATAACATAAAGGGACGATAAGACCCTATAAAACTTTACTTTCT-----------------ATTTTTATAATTTTT---AAATTAACG------TGTAA-----ATTTGTTATTGG-GATAG---------------------A-TGTTTTGT-------------TGGGGCGGCAGGAATATAAA--------ATAT--TAACTGTTCTTGGATT---------------------------------------------------------------------ATGAAA--TAATA----ATAATTAGATTAAT-TGATCCTATACTATGGATTACAAGATTAAGTTACTTTAGGGATAACAGCGTAATTTCTCTTGAGAGACCCTATCGACAGAGTTAGTTGCGACCTCGATGTTGAATTAAGATACCGTTTAGATGCAGGAGTCTAAACAGTAGGTCTGTTCGACCTTTAAAATCTT

14_Lysmata_wurdemanni

gggccgcggta-cactgaccgtgcgaaggtagcataataattagttctttaattgagggctggaatgaacggt-tgaacgagaagttagctgtct-ctaaggtaggtcttgaactttacctttaagtgaaaaggcttaaataatataaagggacgataagaccctataaaactttactttct-----------------a-ttttaaaattttta--aaattaatg------tgtaa-----atttgtttttgg-aagag---------------------a-tgttctgt-------------tggggcgacaggaatataaa--------atac--taactgttcttggaaa---------------------------------------------------------------------agaaaa--taata----ataattagatttga-tgatcctttaatatggattacaagattaagttactttagggataacagcgtaatttctcttgagagaccttatcgacagagctagttgcgacctcgatgttgaattaagatgccgctcaggtgcaggagcctggggagtgggtctgttcgacctttaaaatctt

12_Lysmata_wurdemanni

GGGCCGCGGTA-TACTGACCGTGCGAAGGTAGCATAATAATTAGTTCTTTAATTGAGGACTGGGATGAACGGT-TGGACGAGAAGTTATCTGTCT-CTAAGGTAGGTCTTGAATTTTACCTTTAAGTGAAAAGGCTTAAATAACATAAAGGGACGATAAGACCCTATAAAACTTTACTTTCT-----------------ATTTTTATAATTTTT---AAATTAACG------TGTAA-----ATTTGTTATTGG-GATAG---------------------A-TGTTTTGT-------------TGGGGCGGCAGGAATATAAA--------ATAT--TAACTGTTCTTGGATT---------------------------------------------------------------------ATGAAA--TAATA----ATAATTAGATTAAT-TGATCCTATACTATGGATTACAAGATTAAGTTACTTTAGGGATAACAGCGTAATTTCTCTTGAGAGACCCTATCGACAGAGTTAGTTGCGACCTCGATGTTGAATTAAGATATCGTTTAGATGCAGGAGTCTAAACAGTAGGTCTGTTCGACCTTTAAAATCTT

13_Lysmata_wurdemanni

GGGCCGCGGTA-TACTGACCGTGCGAAGGTAGCATAATAATTAGTTCTTTAATTGAGGGCTGGGATGAACGGT-TGGACGAGAAGTTATCTGTCT-CTAAGGTAGGTTTTGAATTTTACCTTTAAGTGAAAAGGCTTAAATAATATAAAGGGACGATAAGACCCTATAAAACTTTACTTTCT-------------------------ATTTTAGAAATTTTTAAAT-TGACGTGTAA-----ATTTGTTATTGG-AATAG---------------------A-TGTTTTGT-------------TGGGGCGGCAGGAATATAAA--------ATAT--TAACTGTTCTTGGATT---------------------------------------------------------------------ATGAAA--TAATA----ATAATTAGATTAAT-TGATCCTTTAATATGGATTACAAGATTAAGTTACTTTAGGGATAACAGCGTAATTTCTCTTGAGAGACCTTATCGACAGAGTTAGTTGCGACCTCGATGTTGAATTAAGATACCGTTTAGGTGCAGGAGTCTAAACAGTAGGTCTGTTCGACCTTTAAAATCTT

15_Lysmata_gracilirostris

GGGCCGCGGTA-TTCTGACCGTGCGAAGGTAGCATAGTAATTAGTTCTTTAATTGAGGACTGGAATGAACGGT-TGGACGAGAAGTTAGCTGTCT-CTAAGGCAAGGCTTGAATTTTACTTTTAAGTGAAAAGGCTTAAATAAAATAAAGGGACGATAAGACCCTGTAAAACTTAACTTTCT------------------ATTTTAAA-GGTTT-CTGAATT--CT---AAGTGTAAATTT-GCTT--TTAAGG-TAGG------------------------TGTTTTGC-------------TGGGGCGGCAGGAATATAA--------TTAGT--TAACTGTTCTTTTTAGA--------------------------------------------------------------------ATAATA-ATA-------ATAATTAGGTTAAT-TGATCCTCTAATAGGGATTAAAAGATTAAGTTACTTTAGGGATAACAGCGTAATTTCTCTTGAGAGACCAAATCGACAGAGTTAGTTGCGACCTCGATGTTGAATTAAGATATCATTCAGGTGCAGGAGTCTGAATAGTAGGTCTGTTCGACCTTTAAAATCTT

16_Lysmata_nayaritensis

GGGCCGCGGTA-TCTTGACCGTGCTAAGGTAGCATAGTCATTAGTTCTTTAATTGAGGACTGGAATGAAAGGT-TGGATGAGAAGTTAGCTGTCT-TTAAGACAAATCTTGAATTTTACTTCTGAGTGAAAAGGCTTAGATAAGATAAGGGGACGACAAGACCCTATAAAACTTTACAGCTT----------------------------------------ATCT---TAGTATCTGCCC-AATTAAAG-GTG-TAAATGTAGTATTAAGACAGC-------TGTTTTGC-------------TGGGGCGGCAGGAATATAAAG--------AAT--TAACTGTTTCTTTT------------------------------------------------------------------------TTAAAAATAGG---T-TTAATTAG-TAAATGTGATCCTTTAATGAGGATTAAGAGATTAAGTTACTTTAGGGATAACAGCGTAATCTCTCTTGAGAGCCCTGATCGACAGAGTTAGTTGCGACCTCGATGTTGAATTAAGATGTTACCTAGGTGCAGGAGTCTAGGCGGTGGGTCTGTTCGACCTTTAAAATCTT

42_Lysmata_cf._anchisteus

GGGCCGCGGTA-TTTTGACCGTGCAAAGGTAGCATAATGAAATGTCTCTTAATTGGAGACTGGAATGAACGGT-CGGACAAGAAGTAAGCTGTCT-CTTTAATAAAACTGAAATTTTACTTTTAAGTGAAAAGGCTTAAATAATATAAGAAGACGATAAGACCCTATAAAACTTTACAAATC---------------------ATAGA-TATAC--TTTAGTAAGT--TGAGTGTAA-----AAG-GGTGTGTA-TATGAG---------------------TTGTTTTGC-------------TGGGGCGGCAGAGATATAAAC-------AAG---TAACTGTTTTTAGTG-----------------------------------------------------------------------TCAAA-ATACCG----ATATGTAG-TAAACATGGTCCTTTAATAAGGATTAAAAGATCAAGTTACTTTAGGGATAACAGCGTAATTTCTCTTGAGAGTTCTAATCGACAGAGCAAGTTGCGACCTCGATGTTGAATTAAGGTGTTATTCAGGTGCAGCCGCTTGAATAGTGAGTCTGTTCGACTTTTAAAACCTT

43_Lysmata_hochi

GGGCCGCGGTA-TTTTGACCGTGCAAAGGTAGCATAATCAAATGTTTCTTAATTGGAAACCGGAATGAACGGT-CGGACGAGAAGTAAGCTGTCT-CTTTAATAAAGCTTGAATTTTACTTTTAAGTGAAAAGGCTTAAATAACGTAAGAAGACGATAAGACCCTATAAAACTTTACAAGCT------------------------------------------GT-AAAAGTGTAT------CTTAATTTAAGGTGTAACAGGGCATTTGTATAG-----ATTGTTTTGC-------------TGGGGCGGCAGAGATAAAA--------TGAG---TAACTGTTTTTAGTG-----------------------------------------------------------------------TGAAA-ATATTG----ATGTATAG-AAAGTATGATCCTGTATTATGGATTAAAAGAGTAAGTTACTTTAGGGATAACAGCGTAATTTCTCTTGAGAGTTCTGATCGACAGAGTAAGTTGCGACCTCGATGTTGAATTAAGGTGTTATCCAGGTGCAGCAGCTTGGATAGTAGGTCTGTTCGACCTTTAAAACCCT

25_Lysmata_hochi

GGGCCGCGGTA-ATTTGACCGTGCTAAGGTAGCATAATCAATAGTCCTTTAATTGGGGACCGGAATGAACGGT-TGGACAAGAAGTTAACTGTCT-CTTAAATGAATCTTGAAGTTTACTTTTAAGTGAAAAGGCTTAAATGAGTTAAAGGGACGATAAGACCCTATAAAACTTAACAAGTT---------------ATGTAAGTAGT-TATT-----AAATTA-T--GT-GTTTAA-----AATGACTAAAGG-TGTA----------------------ATTGTTTCGT-------------TGGGGCGATTGAGATAAAA--------TTAAT--TAACTGTCTAAAAGTTAT-------------------------------------------------------------------AATAAA-A---------ATAATTAG-TGAGTATGATCCTTTATTAAGGATTAAAAGATTAAGTTACTTTAGGGATAACAGCGTAATTTCTCTTGAGAGTCCTAATCGACAGAGTTAGTTGCGACCTCGATGTTGAATTAAGGTGTTATTCGGGCGCAGAAGCTCGAAAAGTAGGTCTGTTCGACCTTTAAAACCTT

**28S Figure 2**

The 28S alignment is identical to the one used for the 28S gene in the concatenated data set below.

**28S/16S Figure 3**

Lysmata_cf._anchisteus ACCTGGCGGCTTTGTTGGCCGTCCAGGGGTGTTGCGTTCCGGTGCGTCCTTGCCGGCCGAGTTGCTCACCGCCTAAGTCATTGCTTGAAAGCAGCCCCATGGAGGGTGATAGGCCCGTGTGGCGGCGCCTGTAAAAAGGGTGAAGGCGGCTCGGTCGGGCGGACGTCCACCGTAGAGTCGGGTTGCTTAGTACTGCAGCCCTAAGCAGGTGGTAAACTCCATCTAAGGCTAAATATTACCACGAGTCCGATAGAGAACAAGTACCGTGAGGGAAAGCTGAAAAGAACTTTGAAGAGAGAGTTCAATAGGACGTGAAACCGTTAGAAGCCTAAACGGGTGGACCCGCGAAGGTTGAGCGAGGGGATTCAGGTCGCCGATGGGCGGT------------------AGGTT-----CGAGACCCGGCGCGGCGGCCGGGCCCGGCCTCTGCCTCCGTTTCCGGCGGGCTTATTTCTCCCTTGCG-AATCGCCGCGACCCGCTCTGGGGAGCCCAAGGGCCGTGCCGCACTGGTAGTCCGCGCCGCGGTATTTGACCGTGCAAAGGTAGCATAATGAAATGTCTCTTAATTGGAGACTGGAATGAACGGTCGGACAAGAAGTAAGCTGTCTCTTTAATAAAACTGAAATTTTACTTTTAAGTGAAAAGGCTTAAATAATATAAGAAGACGATAAGACCCTATAAAACTTTACAAATCATAGATATACTTTAGTAAGTTGA-TGTAAAAGGGTGTGTATATTTGTTTTGCTGGGGCGGCAGAGATATAAACAACTGTTTTTGTCAAAATACCGATATGTAGTAAACATGGTCCTTTAATAAGGATTAAAAGATCAAGTTACTTTAGGGATAACAGCGTAATTTCTCTTGAGAGTTCTAATCGACAGAGCAAGTTGCGACCTCGATGTTGAATTAAGGTGTTATTCAGGTG

Lysmata_lipkei ACCTGGCGGTCTTGTCGGCCGTCCAGGGGTGTTGCGTTCCGGTGCGTCCTTGCCGCCCGAGTTGCTAACCGCCTAAGTCATTGCTTGAAAGCAGCCCCATGGAGGGTGATAGGCCCGTGTGGCGGCGCCTCTAAAAAGGGTGAAGGCGGCTCGGGCGGGCGGACGTCCACCGTAGAGTCGGGTTGCTTAGTACTGCAGCCCTAAGCAGGTGGTAAACTCCATCTAAGGCTAAATACTACCACGAGTCCGATAGAGCACAAGTACCGTGAGGGAAAGCTGAAAAGAACTCTGAAGAGAGAGTTCAATAGGACGTGAAACCGTTAGAAGCTTAAACGGGTGGACCCGCGAAGGTTGAGCCAGGGGATTCAGCTCGCCGGCTGGCGGAGCGGGAGGAAGG-GTCCGAGACG-AGGACGAGACCCGGCGCGGCTGCCGGGCCCTGCCTCCGCCTCCGGCATCGGCGGGCTTACTTCTCCCTGGCG-AGTCGCCGCGACCCGCTCTGGGGAGCCCAAGGGCCGTGTCGCACTGGTAGTCCGGGCCGCGGTATTTGACCGTGCAAAGGTAGCATAATCAATAGTTTTTTAATTGAAAACTGGAATGAAAGGTCGGACAAGAAGTAGACTGTCTCTTTTATAAGACTTGAATTTTACTTTTAAGTGAAAAGGCTTAAATGGTTTAGGGGGACGATAAGACCCTATAAAACTTAACAGATTTTATTCAGAGAGGATAATTAATG-TTTAAATA-GTTTTTGAGTCTGTTTTACTGGGGCGGTACGGATA-AAATAACTGTCTA-ATTATAATA-TTATAAATAGTAAATTTGATCCTTTACTAGGGATTAGGAGATAAAGTTACTTTAGGGATAACAGCGTAATCTCTCTTGAGAGTCCTAATCGACAGAGTAAGTTGCGACCTCGATGTTGAATTAAGGTGTTATCTAGGCG

Lysmata_cf_trisetacea ACCTGGCGGTCTTTTTGGCCGTCCAGGGGTGTTGCGTTTCGGTGCATCCTTGCCGCCCGAGTTGCTCACCGCCTAAGTCATTGCTTGAAAGCAGCCCCAAGGAGGGTGATAGGCCCGTGTGGCGGCGCCTGTAAAAAGGGTGAAGGCGGCTCGGGCGGGTGGGTGTCCACCGTAGAGTCGGGTTGCTTAGTACTGCAGCCCTAAGCAGGTGGTAAACTCCATCTAAGGCTAAATACTACCACGAGTCCGATAGAGAACAAGTACCGTGAGGGAAAGCTGAAAAGGACTTTGAAGAGAGAGTTCAATAGGACGTGAAACCGTTAGAAGCTTAAACGGGTGGAACCGCGAAGGTTGACCGAGGGGATTCAGCTCGCCGGTGCTCGGAGG-CTGCGGGTGTTTCGGAGAGTCACGACCGGACCCGGCACGGTGGCCGGGCTGCGCCCGTGCCTCCGAGTTCGGCGGGCTTACTTCTCCCTCGGGAAGTCGCCGCGACCCGCTTCCGGGAGCCCCAGGGCCGTGCCGCATTGGTAGCCCGGGCCGCGGTATCTGACCGTGCGAAGGTAGCATAATCAATAGTGTTTTAATTGAAGACTGGAATGAAGGGTCGGACAAGGGGTTATCTGTCTCTTAGATAAATCTTGAAGTTTACTTTTAAGTGAAAAGGCTTAAATGAAGTAAGGGGACGATAAGACCCTATAAAACTTAACAAATTATGCCTTTAGAT-GTACGAATTAGTGTAAACTTGATGGGG-GTTTGTTTTGTTGGGGCGACAGGGATA-AAATAACTGTCTTTATTAAAATAGTTATAATTAGAGTATTTGATCCTTTAGTAAGGATTATAAGATTAAGTTACTTTAGGGATAACAGCGTAATTTCTCTTGAGAGTTCTAATCGACAGAGCTAGTTGCGACCTCGATGTTGAATTAAGATGTTATTCAGGCG

Lysmata_ternatensis ACCTGGCGGTCTTTTTGGCCGTCCAGGGGTGTTGCGTTTCGGTGCATCCTTGCCGCCCGAGTTGCTCACCGCCTAAGTCATTGCTTGAAAGCAGCCCCAAGGAGGGTGATAGGCCCGTGTGGCGGCGCCTGTAAAAAGGGTGAAGGCGGCTCGGGCGGGTGGGTGTCCACCGTAGAGTCGGGTTGCTTAGTACTGCAGCCCTAAGCAGGTGGTAAACTCCATCTAAGGCTAAATACTACCACGAGTCCGATAGAGAACAAGTACCGTGAGGGAAAGCTGAAAAGGACTTTGAAGAGAGAGTTCAATAGGACGTGAAACCGTTAGAAGCTTAAACGGGTGGAACCGCGAAGGTTGACCGAGGGGATTCAGCTCGCCGGTGCTCGGAGG-CTGCGGGTGTTTCGGAGAGTCACGACCGGACCCGGCACGGTGGCCGGGCTGCGCCCGTGCCTCCGAGTTCGGCGGGCTTACTTCTCCCTCGGGAAGTCGCCGCGACCCGCTTCCGGGAGCCCCAGGGCCGTGCCGCATTGGTAGCCCGGGCCGCGGTATCTGACCGTGCGAAGGTAGCATAATCAATAGTGTTTTAATTGAAGACTGGAATGAAGGGTCGGACAAGGGGTTAGCTGTCTCTAAATAAAATCTTGAAATTTACTTTTAAGTGAAAAGGCTTAAATAACGTGAGGGGACGATAAGACCCTATAAAACTTAACAAGTTATACTGTTAGAT-TTACGAATTAGTGTAAACTTGATGGTA-GTTTGTTTTGTTGGGGCGACAGGGATA-AAATAACTGTCTTTGTTAAAATAGTAATAATTAGTGAACTTGATCCTTTAATAAGGATTACAAGATTAAGTTACTTTAGGGATAACAGCGTAATTTCTCTTGAGAGTCCAAATCGACAGAGTTAGTTGCGACCTCGATGTTGAATTAAGATGTTATTCAGGCG

Lysmata_galapagensis ACCTGGCGGTCCCTGTGGCCGTCCAGGGGTGTTGCGTTTCGGTGCATCCTTGCCGCTCGAGTTGCTAACCGCCTAAGTCATTGCTTGAAAGCAGCCCCAAGGAGGGTGATAGGCCCGTGTGGCGGCGCCTGTAAAAAGGGTGAAGGCGGCTCGGGCGGGCGGGTGTCCACCGTAGAGTCGGGTTGCTTAGTACTGCAGCCCTAAGCAGGTGGTAAACTCCATCTAAGGCTAAATACTACCACGAGTCCGATAGAGAACAAGTACCGTGAGGGAAAGCTGAAAAGGACTTTGAAGAGAGAGTTCAATAGGACGTGAAACCGTTAGAAGCTTAAACGGGTGGAACCGCGAAGGTTGAACGAGGGGATTCAGCTCGCCGGTGCTCGGAGG-CGTCGGGCGTGTCGGAGAGGTAGGACCGGACCCGGCACGGTGGCCGGGCTGCGCCCGATCCTCCGGGTTCGGCGGGCTTACTTCTCCCTCGGGAAGTCGCCGCGACCCGCTTCCGGGAGCCCCAGGGCCGTGCCGCATTGGTAGCCCGGGCCGCGGTATCTGACCGTGCGAAGGTAGCATAATCAATAGTGTTTTAATTGAAGACTGGAATGAATGGTCGGACAAGGGGTTTGCTGTCTCTAAGGTAAATCTTGAAGTTTACCTTTAAGTGAAAAGGCTTAAATGGCGTAAAGGGACGATAAGACCCTATAAAACTTGACGGATTGTGCAGTTAGAT-GTATGAATTGGTGTAAGCTTGATAGTT-GTCCGTTTTGTTGGGGCGACAGGGATATAAGTAACTGTCTTTTTAAAAATAATAGTAATTAGTTTAATTGATCCTTTATTAGGGATTACGAGATTAAGTTACTTTAGGGATAACAGCGTAATTTCTCTTGAGAGTTCTAATCGACAGAGTTAGTTGCGACCTCGATGTTGAATTAAGATGTTATTCAGGCG

Lysmata_seticaudata ACCTGGCGGTCTTTGTGGCCGTCCAGGGGTGTTGCGTTTCGGTGCATCCTTGCCGCCCGAGTTGCTCACCGCCTAAGTCATTGCTTGAAAGCAGCCCCAAGGAGGGTGATAGGCCCGTGTGGCGGCGCCTGTAAAAAGGGTGAAGGCGGCTCGGGCGGGTGGGTGTTCACCGTAGAGTCGGGTTGCTTAGTACTGCAGCCCTAAGCAGGTGGTAAACTCCATCTAAGGCTAAATACTACCACGAGTCCGATAGAGAACAAGTACCGTGAGGGAAAGCTGAAAAGGACTTTGAAGAGAGAGTTCAATAGGACGTGAAACCGTTAGAAGCTTAAACGGGTGGAACCGCGAAGGTTGAACGAGGGGATTCAGCTCGCCGGTGCTCGGAGG-CTGCGGGTGTTTCGGAGAGTCACGACCGGACCCGGCACGGTGGCCGGGCTGCGCCCGTGCCTCCGAGTTCGGCGGGCTTACTTCTCCCTCGGGAAGTCGCCGCGACCCGCTTCCGGGAGCCCCAGGGCCGTGCCGCATTGGTAGCCCGGGCCGCGGTATCTGACCGTGCGAAGGTAGCATAATCAATAGTGTTTTAATTGAAGACTGGAATGAAGGGTCGGACAAGGGGTTGGCTGTCTCTTAAATAAAATTTGAAGTTTACTTTTAAGTGAAAAGGCTTAAATAAAGTAGAGGGACGATAAGACCCTATAAAACTTTACAAGATGTGTTGTTAGAG-GTATGAATTAGTGTAAACTTGATAAGA-ACTTGTTTTGTTGGGGCGACAGGGATA-AAACAACTGTCTTTATAATAATAGTTATAATTAGTTAAGTTGATCCTTTATTAAGGATTAAGAGATTAAGTTACTTTAGGGATAACAGCGTAATTTCTCTTGAGAGTTCTAATCGACAGAGTTAGTTGCGACCTCGATGTTGAATTAAGATGTTACTCAGGTG

Alope_orientalis ACCTGGCGGTGTTTTGTGCCGTCCAGGGGTGTTGCGTTAAGGTGGGTTCGTTCCGTGCAAAGAGCTGACCGCCTAAGTCATTGCTTAAAAGCAGCACCATGGAGGGTGATAGGCCCGTGTGGCGGCGCCTGTAAAAAGGGTGTAGGCTGGTTGCACGGGTGAACGTNAACCTGTGAGTCGGGTTGCTTAGTACTGCAGCCCTAAGTAGGTGGTAAACTCCATCTAAGGCTAAATATTAACACGAGTCCGATAGATGACAAGTACCGCGAGGGAAAGCTGAAAAGGACTCTGAAGAGAGAGTTCAACAGGACGTGAAACTGCTAAGAGCTTAAACGGGTGGAGCCGCGAAGGTCGAACGAGGGGACTCAGCCCGCGTGGGTCGGGCGGTTGGG--GCGGCAAGATGCTGG-TGATGTGACCCGGCATGATAGCCGTGGCCCGCCCCCACCGCCGTGTGGCGCGGGCTTACTTCTTGCCTCAAATCTGGCCGCGACTGGTGGTGGGGACCCCAGGGGTGGTGAAAGACTGGACACCCGGATCGCGGTATCTGACCGTGCGAAGGTAGCATAATCAATAGTCTTTTAATTGGAGTCTGGAATGAACGATTGGACAAAAAGTAATCTGTCTTGAGAGTAAAAATTGAAATTTACTTTTAAGTGAGAAGGCTTAAATAAGTTAGAGGGACGATAAGACCCTATAAAACTTTATGAATTTTGGGCCTATCTAGTAATTTTAG-TTTATATTAAGATAGGTTTTCGTTTTATTGGGGCGATAAAAATATAAATAACTGTTTGTACTGGAATAAATATATTTAAAATTGTTGATCCTGTATTATAGATTAGTAGATAAAGTTACTTTAGGGATAACAGCGTTATTCTTTCTGAGAGTTCTTATCGACGAAGGTAGTTGCGACCTCGATGTTGAATTAAAATTTCTTTTAGGTG

Lysmatella_prima ACCTGGCGGTCGAGGAGGCCGTCCAGGGGTGTTGCGTTCCGGTGCGTCCTTGCCGCCCGAGTTGCTGACCGCCTAAGTCATTGCTTGAAAGCAGCCCCATGGAGGGTGATAGGCCCGTGTGGCGGCGCCCTTACAAAGGGTGTAGGCGGCTTGGGCGGGCGGGCGTCCACCGTAGAGTCGGGTTGCCTAGTAACGCAGCCCTAAGTAGGTGGTAAACTCCATCTAAGGCTAAATACTCCCACGAGTCCGATAGTGCACAAGTACCGTGAGGGAAAGCTGAAAAGAACTCTGAAGAGAGAGTTCAAGAGGACGTGAAACCGTTAGAAGCTTAAACGGGTGGAACCGCGAAGGTTGAGCCAGGGGATTCAGCTCGCCGGGGCGAGCGGTGGTATGGCAG-GTCAGAGATT-ATGACCGGACCCGTCGCGGCGAGCGGGCACTGCCGTCGCCCGCG-CGTCGGCGGGCTTACTTCTCCCTGGCG-AGTCGCCGCGACCCGTTCCGGGGGGCCCAAGGGCCGTGCCGCACTGGTAGTCTGGGCCGCGGTATATGACCGTGCAAAGGTAGCATAGTCACTAGTTCTTTAATTGGGTTCTGGAATGAATGGTTGGACAAGAAGAAAACTGTCTAGAATATAAAATTAGAATTTTACTTTTAAGTGAGAAGGCTTAAATAAAATAAGGGGACGATAAGACCCTATAAAACTTGACAAATAAAGTATTACTCCTCGAATTATGAGTTTAAACAGGGGGATACTTTTGTTCAACTGGGGCGGTGCTTATATAAATAACTATAAAAGTTACAATAGTTATAATTAGATTATTAGACCCTTGTATAAGGATTTTGAGATAAAGTTACTTTAGGGATAACAGCGTTATTTCTCTTGAGAGTCCAAATCGACAGAGTAAGTTGCGACCTCGATGTTGAATCAAGATGTTATTCAGGTG

Parhippolyte_mistica ACCTGGCGGTCTTGTTGGCCGTCCAGGGGTGTTGCGTTTCGGTGGATCCTTGCCGCCCGAGCTGCTGACCGCCTAAGTCATTGCTTGAAAGCAGCCCCATGGAGGGTGATAGGCCCGTGTGGCGGCGCCTGTAAAAAGGGTGAAGGCGGCCCGGGCGGACGGATGTTGACCGTAGAGTCGGGTTGCTTAGTAGTGCAGCCCTAAGTAGGTGGTAAACTCCATCTAAGGCTAAATACTACCACGAGTCCGATAGAGAACAAGTACCGTGAGGGAAAGCTGAAAAGGACTTTGAAGAGAGAGTTCAAGAGGACGTGAAACCGTTAGAAGCTTAAACGGGTGGAACGGCGAAGGTGGAGTGAGGGGATTCAGGTCGTGGGC-TTTGGGAGGTGGTGGGTGTCTGTGAGATGTATGAGAGGACCTGGCATA-TGCCTGGGCCGCACCCACCCCTCT---CTCGGCGGGCTTACTTCTCCCTC-TG-TGTCGCCGCCAGCCGCTGGGGGGACCCCTAGGGCCGTCTGCGATTGGTAGCCCGGACCGCGGTATATAACCGTGCGAAGGTAGCATAATCATTTGTCTTTTAATTGAAGGCTGGAATGAATGGTCGAACGAGAAGTAAGCTGTCTCTTTAATGTATACTGAATTTTACCTTTAAGTGAAAAGGCTTAAATGTCACGGGGGGACGATAAGACCCTATAAAGCTTTACGATTACTTGAACTGTGTC-CGAATTTGAGTATAAGGGAGGCA-GGTATTCGTTTCGTTGGGGCGACGAGAATATAACAAACTGTTCTTAATAAAACGGTTGTGATCGGTTGAGTGGACCCTTTATTAAGGATCATTAGATTAAGTTACTTTAGGGATAACAGCGTTATTTCCCTTGAGAGTTCATATCGACAGGGTAAGTTGCGACCTCGATGTTGAATTAAGGTTTCATTCAGGTG

Exhippolysmata_oplophoroides

ACCCGGCGGCCGAG-AGGCCGACCGGGGGTGTTGCGTTCCGGTGCGTCCTTGCCGGCCGAGTTGCTGACCGCCTAAGTCATTGCTTGAAAGCAGCCCCGAGGAGGGTGATAGGCCCGTGTGGCGGCGCCCATAAGAAGGGTGTAGGCGGCTTGGTCGGGCGGACGTCCACCGTAGAGTCGGGTTGCCTAGTAACGCAGCCCTAAGCAGGTGGTAAACTCCATCTAAGGCTAAATACTCCCACGAGTCCGATAGTGCACAAGTACCGTGAGGGAAAGCTGAAAAGAACTCTGAAGAGAGAGTTCAAGAGGACGTGAAACCGTTAGAAGCTTAAACGGGTGGAACCGCGAAGGTTGAGCCAGGGGATTCAGCTCGCCGGTGGGCGGGGCGGGATGGCGGCGTTGGAGATTTACGACCGGACCCGGCGCGGCGACCGGGCCCCGCCGCCGCCCCCG-ATCGGGCGGGCTTACTTCTCCCTGGCT-AGTCGCCGCGACCCGTTCTGGGGAGCCCAAGGGCCGTGCCGCACTGGTAGTCCGGGCCGCGGTACTTGACCGTGCAAAGGTAGCATAATCAGTAGTTCTTTAATTGGGAACTTGTATGAAGGGCTGGACAAGAAGGAACCTGTCTCTAAAATAAAATTTGAATTTCACTTTTAAGTGAAAAGGCTTAAATAAAATAAGGGGACGATAAGACCCTATAAAACTTGACAAGAAATATACTAACTCTT-AGTTCTAC-TTTAAAGGAGGGGGTATGTTTGTTTTACTGGGGCGGTAGTTATATAGGAAACTGTAAAGTATA-AATAATTATAATTAGAGAATATGATCCCTTTTTGGGGATTAAAAGATAAAGTTACTTTAGGGATAACAGCGTTATTTCTCTTGAGAGTCCAAATCGACAGAGTAAGTTGCGACCTCGATGTTGAATCAAGATGTTATTCAGGTG

Merguia_oligodon ACCTGGCAGCCTGATGGGCTGTCCAGGGGTGTTGCGTTCTGGTGCGTCCTTGCTGCCCCGCCTGCTTACCGCCTAAGTCATTGCTTGAAAGCAGCCCCGAGGAGGGTGATAGGCCCGTGTGGCGGCGCCTGTAAAAAGGGTGAAGGCAGACGGGGTGGGCGGACGTCCACCGTAGAGTCGGGTTGCTTAGTAGTGCAGCCCTAAGTAGGTGGTAAACTCCATCTAAGGCTAAATATGACCACGAGTCCGATAGAGAACAAGTACCGTGAGGGAAAGCTGAAAAGGACTTTGAAGAGAGAGTTCAAGAGGACGTGAAACCGTTAGAAGCTCAAACGGGTGGAACCGCGAAGGTTGAATGAGGGGATTCAGCTCTCCGGTGCTCAGGGTGTGGGCGGCGGGCCGGAGGCGTTTCATGCGACCCGGCA-GCTGCCCGGGCTCCGCCCCAGCCTCTGGGTT-GGTGGGCTTACTTCTCCCTCAGT-AATTGCCGCGACCCGTTCTGGGGAGCCCCAGGGCCGTGCCGGACTGGTAACTCTGGCCGCGGTATTTGACCGTGCGAAGGTAGCATAATCAATAGTCTTTTAATTGAGGGCTGGAATGAATGGTTGGACGAGATGGAGGCTGTCTCTAGCGTAAATCTTGAATTTTACTTTTAAGTGAAAAGGCTTAAATGATTTAGTGGGACGATAAGACCCTGTAAAGCTT--CATAATTTTATTTTAGTCAGCAAAATTAGACATAAGTAAACTGTTGGGTTTATTAGGTTGGGGTGACTTAGATATAACAAACTGTCTTAAATATAATAACTATAATTAGCTTAGGTGATCCTTAATTTAGGATTAAAAGATAAAGTTACTTCAGGGATAACAGCGTAATTTCTCTTGAGAGCACATATCGACAGAGTTAGTTGCGACCTCGATGTTGAATTAAGGTAATTTTTAGGCG

Hippolyte_acuta ACCTCGCGGTCTACGTGGCCGTCGAGGGGTGTTGCGTGAAGGTGGATCCGTTCCGTCCGGGCCGCTCACCGCCTAAGTCATTGCTTGAATGCAGCCCCAAGGAGGGTGATAGGCCCGTGTGGCGGCGCCTGTCCCAAGGGTGTAGGCGGCTCGGACGGGTGGGTCTTCACCGTAGAGTCGGGTTGCTTAGTACTGCAGCCCTAAGCAGGTGGTAAACTCCATCTAAGTCTAAATACAACCACGAGTCCGATAGGCAACAAGTACCGTGAGGGAAAGCTGAAAAGGACTTTGAAGAGAGAGTTCAAAAGGACGTGAAACTGCTAGAGGCCTAAACGGGTGGAACCGCGAAGGACGAACGAGGGGATTCAGCCCGCCGGCGGCCGGCGTGCTCAGGACGGCCGTCCCGCGCGCCAAGAGACCCGGCGTGATTGCCGTGGTCCGGCCTGGGTCGTCCGGCGGGCGGGCTTACTTCTCCCTCCATAGG-CGCCGCGACTCGTTGC--GGGCCCCCAGGGCCGCGACGGACTGGTAGCCCGGGCTGCGGTAATTGACCGTGCTAAGGTAGCATAATCAGTAGTCTTTTAATTGATGACTGGAATGAATGGCGTTACGAGAAATAAGCTGTCTTAAAAATAAAAATTGAATTTTACCTTCAAGTGAAAAGGCTTGAATACTACTAAGGGACGATAAGACCCTATAAAACTTAATGATTGTCTTTTCTGAGGGTTAAAGTTGA--TTAAGAATGGGTGTATTT-TGTTTGGTTGGGGCGACCTAGATATAATTAACTGTCTGAATAAAAATAATTATAATTTGGTT-CATGAACCTTTAATAAGGATTAGAAGAAAAAGTTACTTTAGGGATAACAGCGTAATTTTTTCAGAGAGTTCTTATCGAAGAAAGTAGTTGCGACCTCGATGTTGAATTAAAGTTTCTCTTAAGCG

Heptacarpus_futilirostris

ACCTCGCGGCCC-CAGGGCCGTCGAGGGGTGTTGCGTACAGGTGGGTCCGCACCGCCCGTGCCACCTACCGCCTAAGTCGTTGCTTAAAAGCAGCCCACAGCAGGGTGAAAGGCCCGTGTGGCGGCGCCTGTGAAAAGGGTTTAGGCGGCGCGGGCGGAGGGACCTCCACCGGAGAGTCGGGTTGCTTAGTACTGCAGCCCAAAGCAGGTGGTAAACTCCATCTAAGGCTAAATACGACCACGAGTCCGATAGCAGACAAGTACCGTGAGGGAAAGTTGAAAAGCACTCTGAAGAGAGAGT-CAAGAGGACGTGAAACCACTAGAAGCGTAAACGGGTGGAGCCGTGAAGGTCGAACGAGGGGATTCAGCCCGCGGGCGGGCGGCGG-CGTGGGGCGGCGCATACGAATG--AGAGGACCCGGCATAAAGGCCGGTGCCGGCCCCCG-CCGCACGCCCCGCGGGTTTACTTCTCCCGAATCGCA-CGCCGCGAACCCCCGGCTCCTCCCCGAAGGCCGTCCCGGACTGGTAGGCCCGACCGCGGTATCTGACCGTGCGAAGGTAGCATAATCAGTAGTCTCTTAATTGGAGGCTGGAATGAATGGTTGCACGAGAAGAAAGCTGTTTCTTCTTCAAGAATTGAATTTTACTTTTAAGTGAAAAGGCTTAAATAAATTGAAGGGACGATAAGACCCTATAAAACTTTAC-AATTTATTATTGCTTCATAAATTTTTA-TATAACTTGGGTTAGTTGTTTGTTGAGTTGGGGCGACTATTATAAAA-TAACTGTAT-TGAATTAATAATTCTGTTTAGT- TAATTGATCCTTTTTTAAAGATTAAAAGATTAAGTTACTTTAGGGATAACAGCGTGATTTTTTTAAAGAGTTCTTATCGATGAAATTAGTTGCGACCTCGATGTTGAATTAAAATTTCTATAAAGTG

Heptacarpus_geniculatus

ACCTCGCGGCCT-CACGGCCGTCGAGGGGTGTTGCGTACAGGTGGGTCCGCACCGCCCGTGCCACCTACCGCCTAAGTCGTTGCTTAAAAGCAGCCCACAGCAGGGTGAAAGGCCCGTGTGGCGGCGCCTGTGAAAAGGGTTTAGGCGGCGCGGGCGGAGGGACCTTCACCGGAGAGTCGGGTTGCTTAGTACTGCAGCCCAAAGCAGGTGGTAAACTCCATCTAAGGCTAAATACGACCACGAGTCCGATAGCAGACAAGTACCGTGAGGGAAAGTTGAAAAGCACTCTGAAGAGAGAGT-CAAGAGGACGTGAAACCACTAGAAGCGCAAACGGGTGGAGCCGTGAAGGTCGAACGAGGGGATTCAGCCCGCGGGCGGGCGGCGG-AGCGGGGCGGCGCATACGAATG--AGAGGACCCGGCATAATGGCCGGTGCCGGCCCCCG-CCGCACGCCCCGCGGGTTTACTTCTCCCGAATCGCA-CGCCGCGAGCCCCCGGCTCCTCCCCGAAGGCCGTCCCGGATTGGTAGGCCCGACCGCGGTATTTGACCGTGCGAAGGTAGCATAATCAATAGTCTCTTAATTAGAGGCTGGAATGAATGGTTGCACGAGAAAGAAGCTGTCTCTTCTTTAAGAATTGAATTTTACTTTTAAGTGAAAAGGCTTAAATAATCTAAGGGGACGATAAGACCCTATAAAACTTTAC-AATTTATGTTTGCTTCATGAATTTTC--TCTAAATTAAGTGAAATATTTGTTAGGTTGGGGCGACTATTATATAG-TAACTGTAA-TAAAGAAATAAGTTTATTTAGT-TAATTGATCCTTTAATAAAGATTATAAGATTAAGTTACTTTAGGGATAACAGCGTAATTTTTTCTGAGAGTTCTTATCGATGAAATTAGTTGCGACCTCGATGTTGAATTAAAATATCTTTTAAGTG

Lysmata_grabhami ACCTGGCGGTCTGTGTGGCCGTCCAGGGGTGTTGCGTTCCGGTGCGTCCTTGCCGCCCGAGTTGCTCACCGCCTAAGTCATTGCTTGAAAGCAGCCCCGAGGAGGGTGATAGGCCCGTGTGGTGGCGCCTGTAAAAAGGGTGAAGGTGGCTTGGGCGGGCGGACGTCCACCGTAGAGTCGGGTTGCTTAGTACTGCAGCCCTAAGCAGGTGGTAAACTCCATCTAAGGCTAAATACTACCACGAGTCCGATAGAGAACAAGTACCGTGAGGGAAAGCTGAAAAGGACTTTGAAGAGAGAGTTCAAGAGGACGTGAAACCGTTAGAAGCCTAAACGGGTGGAACCGCGAAGGTTGAACGAGGGGATTCAGGTCGCCGGTGGCCGGAGTGCGGAGGGCGGGTCAGACATGTACGACGTGACCCGGCGCGGCGGCCGGGCCCCGCCGCCGCCTCCGGGCCCGGCGGGCTTACTTCTCCCTCGGGAAGTCGCCGCGACCCGTTCCTGGGAGCCCCAGGGCCGCATCGCATTGGTATGCCGGGCCGCGGTATCTGACCGTGCGAAGGTAGCATAATCAATAGTTCTTTAATTGAGGACTGGAATGAAGGGTCGGACGAGAAGTAAGCTGTCTCCAAGGCAAATCTTGAAGTTTACTTTTAAGTGAGAAGGCTTAAATAAGATAAAGGGACGATAAGACCCTATAAAACTTTACAGTATAGGCTTGGAATTTGTTTAAATTTGTGTAAATCTGTT-TTA-GCCTGTTTTATTGGGGCGATAGGGATATAATCAACTGTTTTTGTGAAAATAAAAATAATTAGTTAACTTGATCCTTTAATAAGGATTAGAAGATTAAGTTACTTTAGGGATAACAGCGTAATTTCTCTTGAGAGTTCTAATCGACAGAGTTAGTTGCGACCTCGATGTTGAATTAAGGTGTTAGCTAGGCG

Lysmata amboinensis

ACCTGGCGGTCTGTGTGGCCGTCCAGGGGTGTTGCGTTCCGGTGCGTCCTTGCCGCCCGAGTTGCTCACCGCCTAAGTCATTGCTTGAAAGCAGCCCCGAGGAGGGTGATAGGCCCGTGTGGCGGCGCCTGTAAAAAGGGTGAAGGTGGCTTGGGCGGGCGGACGTCCACCGTAGAGTCGGGTTGCTTAGTACTGCAGCCCTAAGCAGGTGGTAAACTCCATCTAAGGCTAAATACTACCACGAGTCCGATAGAGAACAAGTACCGTGAGGGAAAGCTGAAAAGGACTTTGAAGAGAGAGTTCAAGAGGACGTGAAACCGTTAGAAGCCTAAACGGGTGGAACCGCGAAGGTTGAACGAGGGGATTCAGGTCGCCGGTGGCCGGAGTGCGGAGGGCGGGTCAGACATGTAGGACGTGACCCGGCGCGGCGGCCGGGCCCCGCCGCCTCCTCCGGGCCCGGCGGGCTTACTTCTCCCTCGGGAAGTCGCCGCGACCCGTTCCTGGGAGCCCCAGGGCCGCATCGCATTGGTAGGCCGGGCCGCGGTATCTGACCGTGCGAAGGTAGCATAATCAATAGTTCTTTAATTGAGGACTGGAATGAAGGGTCGGACGAGAAGTTAGCTGTCTCCAAGACAAGTCTTGAAGTTTACTTTTAAGTGAAAAGGCTTAAATGAGATAAAGGGACGATAAGACCCTATAAAACTTTACAGTTTATGTCTAGAATTTGTTTAAATTTGTGTAAATCTGTT-TTG-GTCTGTTTTATTGGGGCGATAAGAATATAATCAACTGTTTTTATGAAAATAGGTATAATTAGTTAATTTGATCCTTTAATAAGGATTAGAAGATTAAGTTACTTTAGGGATAACAGCGTAATTTCTCTTGAGAGTTCTAATCGACAGAGTTAGTTGCGACCTCGATGTTGAATTAAGGTGTTAGCTAGGCG

Lysmata_debelius ACCTGGCGGTCTGTGTGGCCGTCCAGGGGTGTTGCGTTCCGGTGCGTGCTTGCCGCCCGAGTTGCTCACCGCCTAAGTCATTGCTTGAAAGCAGCCCCGAGGAGGGTGATAGGCCCGTGTGGCGGCGCCTGTAAAAAGGGTGAAGGCGGCTCGGGCGGGCGGACGTCCACCGTAGAGTCGGGTTGCTTAGTACTGCAGCCCTAAGCAGGTGGTAAACTCCATCTAAGGCTAAATACTACCACGAGTCCGATAGAGAACAAGTACCGTGAGGGAAAGCTGAAAAGGACTTTGAAGAGAGAGTTCAAGAGGACGTGAAACCGTTAGAAGCTTAAACGGGTGGAACCGCGAAGGTTGAACGAGGGGATTCAGCTCGCCGGTGCCCGGAGTGCGGAGGGCGGGTCAGACATGTAGGATGGGACCCGGCGCGGGTGCCGGGCCCCGCCGCCGCCTCCGGGCCCGGCGGGCTTACTTCTCCCTCGGGAAGTCGCCGCGACCCGTTCCCGGGGGCCCCAGGGCCGCCTCGCATTGGTAGGCCGGGCCGCGGTATTTGACCGTGCGAAGGTAGCATAATCAATAGTTCTTTAATTGAGGACTGGAATGAAGGGTCGGACGAGAAGTAAGCTGTCTCTAAAATAAATCTTGAAGTTTACTTTTAAGTGAAAAGGCTTAAATAAGATAAAGGGACGATAAGACCCTATAAAACTTTACAGATTACATCTAAAATTTGTTTGAAATTGTGTAAATTCGTT-TTA-GTCTGTTTTGTTGGGGCGACAGAGATATAATTAACTGTTTTTATTAAAATAATACTAATTAGTTAATTTGATCCATTAATAAGGATTAAAAGATTAAGTTACTTTAGGGATAACAGCGTAATTTCTCTTGAGAGTTCTAATCGACAGAGTTAGTTGCGACCTCGATGTTGAATTAAGGTGTTAGCTAGGCG

Lysmata_wurdemanni ACCTGGCGGTCTGCGCGGCCGTCCAGGGGTGTTGCGTTCCGGTGCGTGCTTGCCGCCCGAGTTGCTCACCGCCTAAGTCATTGCTTGAAAGCAGCCCCGAGGAGGGTGATAGGCCCGTGTGGCGGCGCCTGTAAAAAGGGTGAAGGCGGCTCGGGCGGGCGGACGTCCACCGTAGAGTCGGGTTGCTTAGTACTGCAGCCCTAAGCAGGTGGTAAACTCCATCTAAGGCTAAATACTACCACGAGTCCGATAGAGAACAAGTACCGTGAGGGAAAGCTGAAAAGGACTTTGAAGAGAGAGTTCAATAGGACGTGAAACCGTTAGAAGCTTAAACGGGTGGAACCGCGAAGGTTGAACGAGGGGATTCAGCTCGCCGGTGCCCGGAGTGCGGAGGGCGGGTCAGACATGTACGACGGGACCCGGCGCGGCTGCCGGGCCCCGCCGCCGCCTCCGGGCCGGGCGGGCTTACTTCTCCCTCGGGAGGTCGCCGCGACCCGTTGTCGGGGGCCCCAGGGCCGTGCCGCATTGGTAGCCCGGGCCGCGGTATCTGACCGTGCGAAGGTAGCATAATAATTAGTTCTTTAATTGAGGACTGGGATGAACGGTTGGACGAGAAGTTATCTGTCTCTAAGGTAGGTCTTGAATTTTACCTTTAAGTGAAAAGGCTTAAATAACATAAAGGGACGATAAGACCCTATAAAACTTTACTTTCTATTTTTATAATT--TTTAAATTAGTGTAAATTTGTTATTG-GGATGTTTTGTTGGGGCGGCAGGAATATAAAAAACTGTTCTTTATGAAATAATAATAATTAGATTAATTGATCCTATACTATGGATTACAAGATTAAGTTACTTTAGGGATAACAGCGTAATTTCTCTTGAGAGACCCTATCGACAGAGTTAGTTGCGACCTCGATGTTGAATTAAGATACCGTTTAGATG

Lysmata_boggessi ACCTGGCGGTCGCCGCGGCCGTCCAGGGGTGTTGCGTTCCGGTGCGTGCTTGCCGCCCGAGTTGCTCACCGCCTAAGTCATTGCTTGAAAGCAGCCCCGAGGAGGGTGATAGGCCCGTGTGGCGGCGCCTGTAAAAAGGGTGAAGGCGGCTCGGGCGGGCGGACGTCCACCGTAGAGTCGGGTTGCTTAGTACTGCAGCCCTAAGCAGGTGGTAAACTCCATCTAAGGCTAAATACTACCACGAGTCCGATAGAGAACAAGTACCGTGAGGGAAAGCTGAAAAGGACTTTGAAGAGAGAGTTCAATAGGACGTGAAACCGTTAGAAGCTTAAACGGGTGGAACCGCGAAGGTTGAACGAGGGGATTCAGCTCGCCGGTGCCCGGAGTGCGGATGGCGGGTCAGACATGTACGACGGGACCCGGCGCGGCTGCCGGGCCCCGCCGCCGCCTCCGGGCCGGGCGGGCTTACTTCTCCCTCGGGAGGTCGCCGCGACCCGTTGTCGGGGGCCCCAGGGCCGTGTCGCATTGGTAGCCCGGGCCGCGGTACCTGACCGTGCAAAGGTAGCATAGTAATTAGTTCTTTAATTGAGGACTGGAATGAACGGTTGGACGAGAAGTTAGCTGTCTCTAATGTAAATCTTGAAGTTTACTTTTAAGTGAAAAGGCTTAAATAATATAAAGGGACGATAAGACCCTATAAAACTTTACTTCTTGTTTAAAAAGTA--GTTGAATTAGTATAAATTTGCTTTTG-GAGTGTTTTGTTGGGGCGATAGAAATATAAATAACTGTTTTTCA-AAAATAATGATAATTAGTTTAAATGATCCTTTAGTATGGATTATAAGATTAAGTTACTTTAGGGATAACAGCGTAATTTCTCTTGAGAGACCTAATCGACAGAGTTAGTTGCGACCTCGATGTTGAATTAAGATATCATTTAGGTG

Synalpheus_brevicarpus

ACCTCGCAGTCC-AACGGCTGTCGAGGGGTGTTGCGTTTCGGTGGGTCCGTCCCGCTCGCGCTGCTAACCGCCTAAGTCATTGCTTGAAAGTAGCCCCGTGGAGGGTGATAGGCCCGTGTGGCGGCGCCTATAAAAAGGTAGGAGGCGGCGTGGGCGGAGGGATCTTCACCGCAGAGTCGGGTTGCTTAGTACTGCAGCCCTAAGAAGGTGGTAAACTCCATCTAAGGCTAAATACTACCACGAGTCCGATAGATAACAAGTACCGTGAGGGAAAGCTGAAAAGAACTTTGAAGAGAGAGTTCAATAGGACGTGAAACCATTAGAAGCATAAACGGGTGGAACCACGATGGTCGAACGAGGGGATTCAGCTCACGGGCCCCCGGC---CGGAGGGTGGC---CAGAAAGG--AGAGGACCGGGCGTCGAGGCCTCGGTCCGACCCT--CTGTAAGGCCCGTGGGCTTACTTCTCCC---TCGCGGCGCCACGACCCGCTGCC-CGGCTCCTAGGGCCTGTCTGGACTGGTAGCCGGGACTGCGGTAATTGACCGTGCAAAGGTAGCATAATCAATAGTCTTTTAATTGGAGGCTTGGATGAAAGGTTTGACGGATGAGGAGCTGTCTTTTAGTTGTG--TTGAACTTAACGTTTGTGTGAAAAGGCATTAATGGTTTAGGGGGACGATAAGACCCTATAAAACTTAACATGTGTGGTGTTAGCTTTTGGAGTTGTT-TGT-AATCATAAGGTGACT-TGTTTTGCTGGGGCGGCACGAATATAATTAACTGTTTTAAAAAAAATATTGATTAATAGATT--TTGGTCCTTTATTAGGGAGT-TAAGAGTAAGTTACTTTAGGGATAACAGCGTAATTTTTCTTGAGAGTTCTTATCGAAGGAAGTAGTTGTGACCTCGATGTTGAATTAAATTTTCCCTTTGGTG

Barbouria_cubensis ACCTGGCGGTCTGGCAGGCCGTCCAGGGGTGTTGCGTTTCGGTGGATCCTTGCCGCCCGAGCTGCTGACCGCCTAAGTCATTGCTTGAAAGCAGCCCCGTGGAGGGTGATAGGCCCGTGTGGCGGCCCCTGTAAAAAGGGTGAAGGCGGCCCGGGCGGACGGATGTTGACCGTAAAGTCGGGTTGCTTAGTAGTGCACCCCTAAGTAGGTGGTAAACTCCATCTAAGGCTAATTACTACCACGAGTCCGATAGAGAACAAGTACCGTGAGGGAAAGCTGAAAAGGACTTTGAAGAGAGAGTTCAAGAGGACGTGAAACCGTTAGAAGCTTAAACGGGTGGAACSGCGAAGGTGGAGCGAGGGGATTCAGGTTGTCTGCGTCCGGGGTGGGGTYGGTGCGCA-GAGATGTATGAGAGGACCCGGCATA-TGCCTGGGCAGCACCCCCGTCCCCGGGTTCGGCCGGCTTACTTCTCCCTCGTGATGTTGCCGCCAGCCGCTTGGGGGACCCCCAGGGCCGGTCGCGATTGGTAGCCCGGGCCGCGGTATCTGACCGTGCGAAGGTAGCATAATAATTTGTCTTTTAATTGAAGGCTCGTATGAATGGTCGGACAAGAAGAAGGCTGTCTTATTAATGAAAGCTGAATTTTACTTTTAAGTGAGAAGGCTTAAATAAACCAAGGGGACGATGAGACCCTATAAAACTTTACAAGATCTTAGGTTGTCCTGTGAATTAGAGTATAAAACAGGTATAGCTTTTGTTTTGTTGGGGCGACAGGAATA-AAAAAACTGTTCTTAATGAAATGGTTATGACCAGGTAGATG-ATCTTTTTTTAAGGATTAAAAGAGTAAGTTACTTTAGGGATAACAGCGTTATTTCCCCTGAGAGGTCTTATCGACGGGGTAAGTTGCGACCTCGATGTTGAATTAAGGTTTCACCCAGACG
