# Supplementary figures and images for: The evolution of euhermaphroditism in caridean shrimps: a molecular perspective of sexual systems and systematics
Source: BMC Evol Biol. 2010 Sep 29;10:297. doi: 10.1186/1471-2148-10-297 (PMC2958167; doi:10.1186/1471-2148-10-297)

**A**

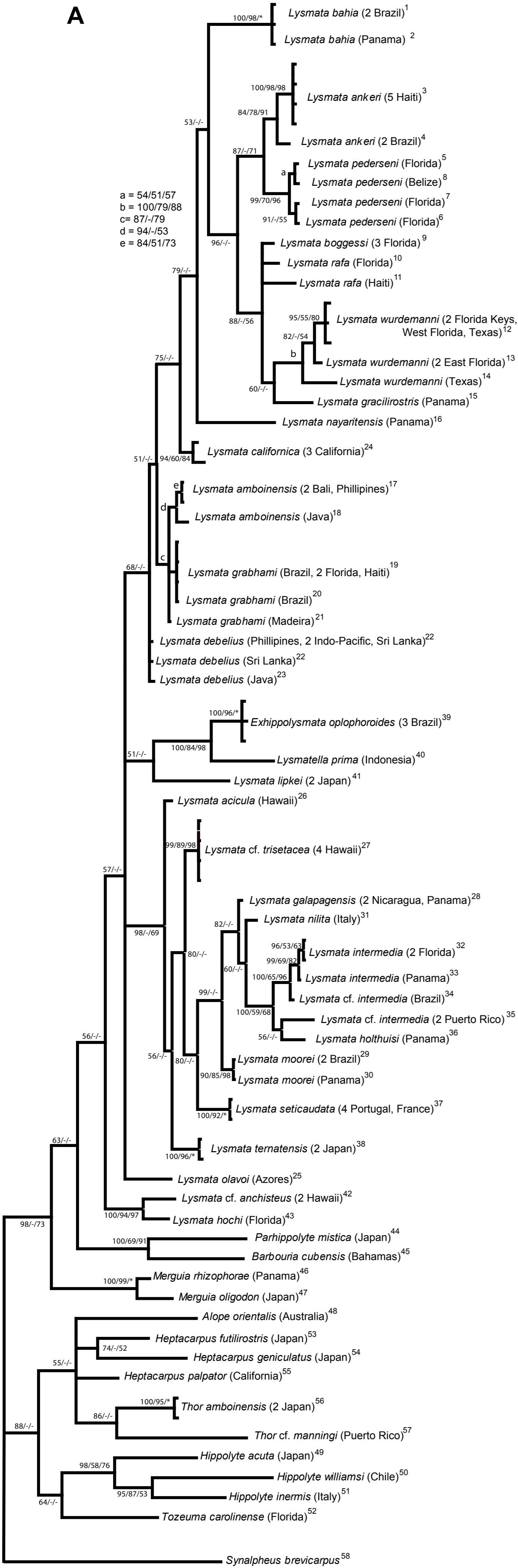

**B**

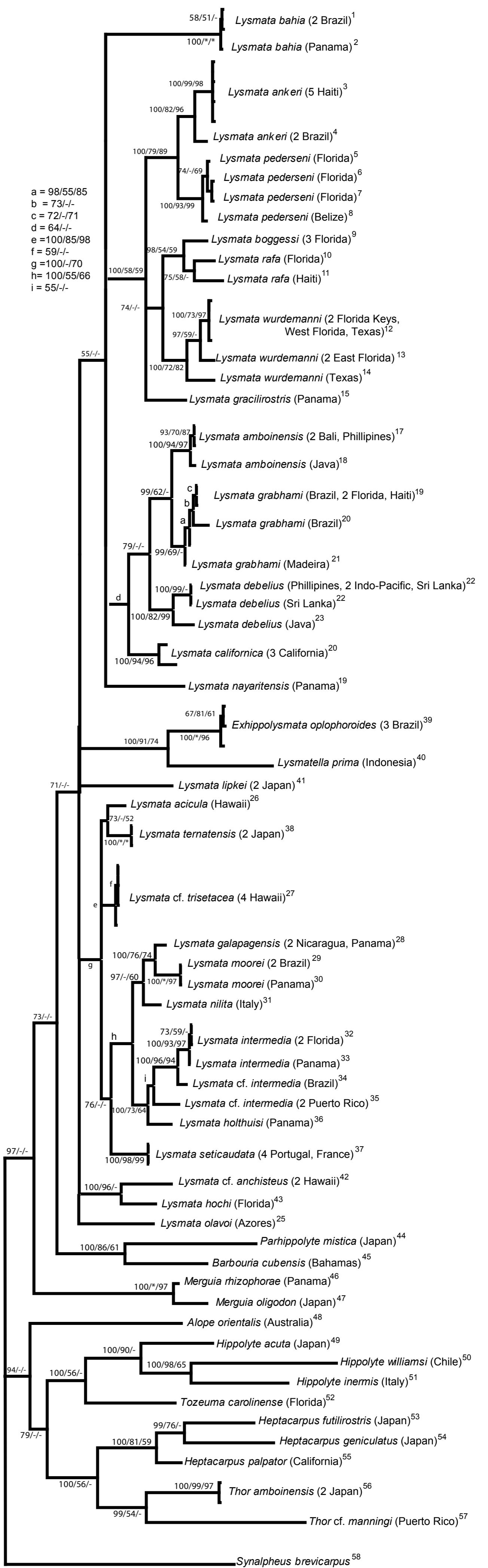

Supplement: Additional file 2 — Figure S1: Bayesian phylogenies of Lysmata and other related genera based on alternative alignment strategies of mitochondrial 16S sequences. Tree A was constructed after the removal of highly variable alignment regions via GBlocks using the most stringent criteria. Tree B was constructed using the alignment resulting from the default settings in ClustalX. Clade support values are shown along the corresponding branches (Bayesian Inference/Maximum Likelihood/Maximum Parsimony). Numbers before sample locations represent the number of specimens sequenced. Superscript numbers indicate which sequences/taxa are represented on the tree (see Tree Identifier in Table 1). [file 1471-2148-10-297-S2.PDF]
